# Supplementary material for: Comparison of compensatory shoulder movements, functionality and satisfaction in transradial amputees fitted with two prosthetic myoelectric hooks
Source: PLoS One. 2023 Feb 2;18(2):e0272855. doi: 10.1371/journal.pone.0272855 (PMC9894487; doi:10.1371/journal.pone.0272855)

**CLINICAL TRIAL**

**PROTOCOL**

Randomized crossover trial comparing shoulder abduction, manual dexterity and satisfaction of upper limb amputees using the Axon-Hook and the Greifer.

| PROMOTOR : | OTTO BOCK FRANCE  4 rue de la Réunion CS 90011 - 91978 COURTABOEUF Cedex  Tel : 01 68 18 88 30 / Fax : 01 69 07 18 02 |
| --- | --- |
| **CLINICAL TRIAL PROTOCOL**  **Short Title**  Assessment of handicap compensation with the Axon-Hook | |
| CLINICAL TRIAL CODE | ESSAI-2016-AXON-HOOK |
| N° ID RCB (ANSM) | 2016-A00897-44 |
| MEDICAL DEVICE | AXON HOOK Otto Bock  Myoelectric hook for external upper limb prosthesis – Medical device class I  Product reference : 8E600 |
| FULL TITLE | Randomized crossover trial comparing shoulder abduction, manual dexterity and satisfaction of upper limb amputees using the Axon-Hook and the Greifer. |
| CLINICAL PHASE | Axon-Hook device has the CE marking and will be used within the framework defined by the CE marking |
| INDICATION(S) (TARGET) | - persons with upper limb amputation at the trans radial-ulnar level - persons with acquired or congenital amputation - persons using a myoelectric prosthesis and having a good control of it - persons whose residual limb is stabilized and who are at least six months away from the amputation - persons whose professional activity or life project justifies or could justify the use of a myoelectric hook - persons who have given their free and informed written consent |
| PRINCIPAL INVESTIGATOR | Dr. Amélie TOUILLET – Rehabilitation doctor (Physical and Rehabilitation Medicine)  Physical and Rehabilitation Medicine Institut - 75 boulevard Lobau 54042 Nancy – France |
|  |  |
| PROTOCOL VERSION | Version 2 |
| DATE OF PROTOCOL | July, 18 2016 |
| ETHICS COMMITEE | Approved on August, 29^th^ 2016  By the ethics committee : Comité de Protection des Personnes EST III |
| ANSM | Approved on July, 19^th^ 2016 |
|  | |

**Abbreviations list**

| ANSM | Agence Nationale de Sécurité du Médicament et des produits de santé des Produits de Santé - French Health Authorities |
| --- | --- |
| B&B | Box and Blocks |
| CNEDiMTS | Commission Nationale d’Evaluation des Dispositifs Médicaux et des Technologies de Santé - National Commission for the Evaluation of Medical devices and Health Technologies |
| CNIL | Commission Nationale de l’Informatique et des Libertés en France - Data protection |
| CPP | Comité de Protection des Personnes en France - Ethics Comittee in France |
| MD | Medical Device |
| AEv | Adverse Event |
| SAEv | Severe Adverse Event |
| AEf | Adverse Effect |
| SAEf | Severe Adverse Effect |
| ESAT | Evaluation de la Satisfaction envers une Aide Technique (french version of the QUEST) |
| QUEST  HAS | Quebec User Evaluation of Satisfaction with assistive Technology  Haute Autorité de Santé - French Health Authorities |
| MPR | Médecine Physique et de Réadaptation - Rehabilitation medicine |
| PMSI  LPPR | Programme de médicalisation des systèmes d’information - Medicalization program of information systems  Liste des Produits et Prestations Remboursables – List of products and allowances reimbursable |
|  |  |

**SUMMARY**

[1 General 7](#_Toc108617979)

[1.1 Identification of the clinical investigation plan 7](#_Toc108617980)

[1.2 Promoter 7](#_Toc108617981)

[1.3 Investigator 7](#_Toc108617982)

[1.4 Supervisory committee 7](#_Toc108617983)

[1.5 General synopsis of the clinical trial 8](#_Toc108617984)

[2 Medical device under investigation 11](#_Toc108617985)

[2.1 General description of the device 11](#_Toc108617986)

[2.2 Intended destination of the device 11](#_Toc108617987)

[2.3 Intended objective of clinical investigation 11](#_Toc108617988)

[2.4 Population and indications 11](#_Toc108617989)

[2.5 Instructions for setting-up and using the device 11](#_Toc108617990)

[2.6 Contraindications 12](#_Toc108617991)

[2.7 Manufacturer and distribution 12](#_Toc108617992)

[2.8 Identification et traceability 12](#_Toc108617993)

[3 Justification of the design of the clinical trial 12](#_Toc108617994)

[3.1 Description of the existing care and its limits 12](#_Toc108617995)

[3.2 Results of available trials relevant to the study 12](#_Toc108617996)

[4 Risks and benefits of the medical device under investigation and of the clinical trial 12](#_Toc108617997)

[4.1 Expected clinical benefits 12](#_Toc108617998)

[4.2 Risks 12](#_Toc108617999)

[4.3 Steps taken to control or mitigate risks 12](#_Toc108618000)

[4.4 Justification of the benefit/risk ratio 12](#_Toc108618001)

[5 Objectives and hypotheses of the clinical trial 13](#_Toc108618002)

[5.1 Primary and secondary objectives 13](#_Toc108618003)

[5.1.1 Primary objective 13](#_Toc108618004)

[5.1.2 Secondary objectives 13](#_Toc108618005)

[5.2 Primary and secondary hypothesis 13](#_Toc108618006)

[5.2.1 Primary hypothesis 13](#_Toc108618007)

[5.2.2 Secondary hypothesis 13](#_Toc108618008)

[6.5 Practical flow of the clinical investigation 15](#_Toc108618009)

[6.5.1 Recruitment methods 15](#_Toc108618010)

[6.5.2 Information and consent collection procedures 16](#_Toc108618011)

[6.5.3 Randomization methods 16](#_Toc108618012)

[6.5.4 Prosthetic devices and rehabilitation program 16](#_Toc108618013)

[6.5.5 Equipment required for the clinical investigation 16](#_Toc108618014)

[6.5.6 Discontinuation of study or premature termination of follow-up 17](#_Toc108618015)

[6.5.7 Subjects replacement procedures, if applicable 18](#_Toc108618016)

[6.5.8 Chronology of visits 18](#_Toc108618017)

[6.5.9 Duration of participation for each subject and total duration of the clinical investigation 18](#_Toc108618018)

[6.6 Management of adverse events 18](#_Toc108618019)

[6.6.1 Definitions 18](#_Toc108618020)

[6.6.2 List of foreseeable adverse events and adverse effects expected from the device, their probable incidence, means of mitigation or treatment 19](#_Toc108618021)

[6.6.3 Role of the investigator 19](#_Toc108618022)

[6.6.4 Role of the promoter 19](#_Toc108618023)

[6.7 Monitoring plan 20](#_Toc108618024)

[7 Statistics 20](#_Toc108618025)

[7.1 Sample size 20](#_Toc108618026)

[7.2 Treatment of missing, unused or erroneous data 21](#_Toc108618027)

[7.3 Analysis populations 21](#_Toc108618028)

[7.4 Statistical analyzes 21](#_Toc108618029)

[8 Quality control and assurance 21](#_Toc108618030)

[8.1 Promoter 21](#_Toc108618031)

[8.2 Data collection 21](#_Toc108618032)

[9 Data processing 22](#_Toc108618033)

[9.1 CNIL 22](#_Toc108618034)

[9.2 Data collected 22](#_Toc108618035)

[9.3 Data recording 23](#_Toc108618036)

[9.4 Data archiving 23](#_Toc108618037)

[10 Ethical considerations 23](#_Toc108618038)

[10.1 Committee for the Protection of Persons 23](#_Toc108618039)

[10.2 Patient information and consent 23](#_Toc108618040)

[10.3 Financial compensation for subjects 24](#_Toc108618041)

[11 Financing and assurance 24](#_Toc108618042)

[11.1 Financing 24](#_Toc108618043)

[11.2 Assurance 24](#_Toc108618044)

[11.3 Amendments to the clinical investigation plan 24](#_Toc108618045)

[12 Devices count 24](#_Toc108618046)

[13 Premature termination or suspension of the clinical investigation 24](#_Toc108618047)

[14 Provisional study calendar 24](#_Toc108618048)

[15 Bibliography 26](#_Toc108618049)

[16 Annexes 26](#_Toc108618050)

[16.1 Patient information letter 26](#_Toc108618051)

[16.2 Patient consent form 29](#_Toc108618052)

[16.3 Observation notebook 30](#_Toc108618053)

[16.4 QUEST Questionnaire 35](#_Toc108618054)

[16.5 Declaration of conformity with the CNIL 38](#_Toc108618055)

# General

## Identification of the clinical investigation plan

Study title: Randomized crossover trial comparing shoulder abduction, manual dexterity and satisfaction of upper limb amputees using the Axon-Hook and the Greifer.

Short title: Assessment of handicap compensation with the Axon-Hook.

Reference code: ESSAI-2016-AXON-HOOK

Version and date of the clinical investigation plan: Version 2 of July, 18th 2016

## Promoter

Promoter’s identity

OTTO BOCK France

4 rue de la Réunion, CS 90011, 91978 COURTABOEUF Cedex, Tel : 01 68 18 88 30 / Fax : 01 69 07 18 02

Responsible of the study at the promoter’s level

Dr. Philippe Henry Medical Director Otto Bock France

Coordination and monitoring of the study at the promoter's level

Ing. Benoît Ponsan Market Access Responsible Otto Bock France

Ing. Aurélie Lacroix Certified Prosthetist Responsible for Quality Insurance Otto Bock France

## Investigator

Investigator

Dr. Amélie Touillet Rehabilitation Doctor

Physical and Rehabilitation Medicine Institut

75 boulevard Lobau 54042 Nancy – France

## Supervisory committee

The trial does not require any supervisory committee since it is not performed in a double-blind fashion. Any serious and unexpected adverse event likely to be due to the device under investigation or any serious adverse event that may be related to the act of fitting the device will be communicated to the competent authorities.

## General synopsis of the clinical trial

| Title | Randomized crossover trial comparing shoulder abduction, manual dexterity and satisfaction of upper limb amputees using the Axon-Hook and the Greifer. |
| --- | --- |
| Short titLe | Assessment of handicap compensation with the Axon-Hook |
| Promotor | Otto Bock France |
| Investigator | Dr. Amélie TOUILLET – Rehabilitation Doctor (Physical and Rehabilitation Medecine)  Regional Institute of Physical and Rehabilitation Medicine - 75 boulevard Lobau 54042 Nancy – France |
| Protocol version | Version 2 of July, 18^th^ 2016 |
| Justification / context | For certain professional or leisure activities, myoelectric prostheses users will find a better functional response with a non-morphological terminal device. Currently, the Greifer, listed under the LPPR (List of reimbursable products and services) under the brand name in Otto Bock's Myobock system, is a widely used hook. It has two movable parallel jaws for gripping objects and a wrist that is orientable and lockable in medio-lateral tilt. The absence of flexion / extension wrist movements associated with the volume of the device which limits visibility, induce compensatory movements such as increased shoulder abduction.  According to INRS data (1), work involving movements or the maintenance of the shoulder without abduction support with an angle greater than or equal to 60 ° for at least two hours per day cumulatively may be at the origin of musculoskeletal disorders of the shoulder. Thus, the more a subject is exposed to large and frequent abduction movements of the shoulder, the more likely he is to develop periarticular conditions of the shoulder. It is important to note that the shoulder of the amputee is under additional stress compared to the healthy subject, due to the weight of the prosthesis.  In 2015, Otto Bock launched the Axon-Hook, differentiating it selves from the Greifer by the possible flexion / extension of the wrist and by thinner jaws, one of which is fixed to allow the gripping of objects with more precision and ease.  This study aims to demonstrate the reduction in abduction movements of the shoulder with Axon-Hook compared to Greifer and will be used to request reimbursement of this device from Health Authorities (HAS).   1. <http://www.inrs-mp.fr/mp/cgi-bin/mppage.pl?tabkey=TAB_RG57> |
| Primary objective | To compare shoulder abduction movements during object movements with the Axon-Hook and the Greifer hooks in subjects with trans radial-ulnar amputations using a myoelectric prosthesis. |
| Secondary objectives | To compare the percentage of time spent with a shoulder abduction greater than 60 ° during the Box and Blocks test, manual dexterity and satisfaction with the Axon-Hook and the Greifer in subjects with trans radial-ulnar amputations using a myoelectric prosthesis. |
| Primary outcome measurement | - Measurement of the shoulder abduction angle during the Box and Blocks test. A reduction in the mean abduction angle when handling objects is an improvement in the service provided by the device because the person decreases the compensatory movements when using the prosthesis. |
| Secondary outcome measurement | - Percentage of time spent with an average shoulder abduction greater than or equal to 60 ° during the Box and Blocks test - Manual dexterity : Box and Blocks test score - Satisfaction : score obtained at the QUEST self-questionnaire (Quebec User Evaluation of Satisfaction with assistive Technology) - Patient preference between the Greifer and the AxonHook |
| Experimental plan | Monocentric, comparative, randomized, crossover, non-blinded study |
| Inclusion criteria | - persons with upper limb amputation at the trans radial-ulnar level - persons with acquired or congenital amputation - persons who regularly use a myoelectric prosthesis and who have a good control of it - persons whose residual limb is stabilized and who are at least six months away from the amputation - persons whose professional activity or life project justifies or could justify the use of a myoelectric hook - persons who have given their free and informed written consent |
| Non-inclusion criteria | - persons under 18 - pregnant women - persons in emergency situations - persons unable to personally give their consent - persons who are psychically or linguistically unable to understand the instructions for taking the research tests - persons not available to comply with the entire study protocol |
| Devices | - AXON-HOOK: Axon-Hook is a myoelectric hook of the Axon-Bus system for external prosthesis of the upper limb. It has a fixed lateral fingertip and a movable middle fingertip as well as a flexible wrist that can be orientated and locked in flexion / extension. The grips are made according to an angular movement. The Axon-Hook is a non-morphological terminal device for myoelectric prosthesis of the upper limb. Its technical characteristics make it a tool that combines strength, precision and resistance to environmental constraints for carrying out manual activities for professional or leisure. The Axon-Hook is an alternative to the Greifer myoelectric hook. - GREIFER: Greifer is a myoelectric hook from the myobock system for external prosthesis of the upper limb. It has two movable fingertips and a wrist adjustable and lockable in medio-lateral tilt. The holds are made in a parallel movement. This device manufactured and distributed by Otto Bock appears in the LPPR under the brand name. This is the study comparator. |
| Practical test procedure | During the inclusion period, the investigator offers all his patients followed by the center and meeting the inclusion criteria to participate to the study. The order of evaluation of the devices is randomized. This is done through sealed envelopes, prepared by the sponsor and given to the investigator before the beginning of the study. Each envelope contains an order of evaluation of the devices: 4 envelopes mention the test order "Axon-Hook then Greifer" and 4 other envelopes mention the test order "Greifer then Axon-Hook". During the inclusion visit and after obtaining the patient's consent to participate, the investigator draws lots for an envelope in order to define the order of testing the devices.  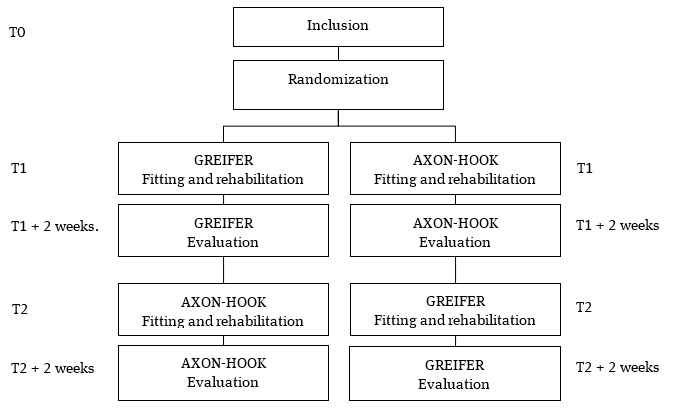  At the beginning of each trial period, the patient benefits from a minimum of one rehabilitation session to ensure that he or she has a good control of the hook. After two weeks, the patient is evaluated with the device (tolerance of + 2 weeks). |
| Number of patients | The calculation of the number of patients to be included is based on the expected difference on the primary outcome measurement, the average shoulder abduction when doing the Box and Blocks test. With the assumption of an average abduction difference of 30 ± 25 degrees, it is necessary to include 8 patients for a randomized cross-over trial. |
| Provisional calendar | Authorization CPP and ANSM : July, 31^st^ 2016  Beginning of the inclusions and of the study : August, 1^st^ 2016  End of the inclusions : September, 30^th^ 2016  End of the study : November, 30^th^ 2016 |
| Statistical method | The study being in crossover, the carry-over effect will be tested in order to test the interaction between the two prostheses using a paired Student's test. If the effect is significant, only the first period will be analyzed using a Student test or the nonparametric Wilcoxon test if the assumptions of normality and equality of variances are not verified. If the effect is not significant, the period effect and the prosthesis effect can then be tested using a paired Student's test. |
| Expected benefits | The results should show a decrease in shoulder abduction, a decrease in the time spent with a shoulder abduction greater than 60 ° and an increase in the satisfaction of people fitted with the Axon-Hook, in comparison with the Greifer.  No significant difference is expected regarding manual dexterity. |

# Medical device under investigation

## General description of the device

Axon-Hook is a myoelectric hook consisting of a fixed lateral fingertip and a movable middle fingertip, both hook-shaped to ensure the gripping of objects in an angular motion. The fact that only one of the two fingertips is movable brings precision to the gripping since the user will position the fixed fingertip against the object to be grasped and then simply perform the closing control movement so that the hook closes. The fingertips are made of titanium and are covered with a polyurethane coating to ensure good grip of the holds. Their small shape allows the user to see his catch clearly. Axon-Hook also has a flexible wrist that can be orientated and locked in flexion / extension in order to position the hook optimally in relation to the object to be gripped. It is covered with a protective cuff. Positioning and locking / unlocking is provided by the contralateral limb. Depending on the patient's wishes and control abilities, wrist rotation can be passive or motorized. The control of the Axon-Hook is myoelectric and the product is supplied with energy by the battery integrated into the socket. This hook is part of Otto Bock's Axon-Bus modular prosthetic system.

## Intended destination of the device

Axon-hook is exclusively intended for the exo-prosthetic devices of the upper limbs.

## Intended objective of clinical investigation

Axon-hook should allow its user to limit shoulder abduction movements when moving objects, improve manual dexterity and increase sa satisfaction.

## Population and indications

**Population**

According to the PMSI data for 2014, there were 147 cases of upper limb amputation for the levels between trans-radial amputation and shoulder disarticulation.

A hook is an additional device that the patient can fix to his prosthesis in place of his prosthetic hand to carry out professional or leisure activities that are difficult or impossible to carry out with a morphological hand. Axon-Hook uses an Axon-Bus electronic system, compatible with the electronics of the Michelangelo morphological hand. According to the CNEDiMTS publication of January 12, 2016, a maximum of 260 Michelangelo myoelectric prostheses could be adapted per year in France, for the first time, second time or renewal. The target population for the Axon-Hook can be estimated at 20% of the target population for Michelangelo, which is to say approximately 50 cases per year.

**Indications**

Axon-hook Axon-hook can be used for unilateral or bilateral amputations, from trans-radial or trans-humeral amputation level or in case of dysmelia for forearm or arm fittings.

## Instructions for setting-up and using the device

Instructions for setting-up

The settings of the Axon-Hook are customized using a computer and the Axon-Soft II software according to the patient's profile and his control capabilities. Only a prosthetist who has received training from Otto Bock on this device is authorized to do the fitting and to adjust the prosthesis. The attribution of an Axon-hook must be done within the framework of a follow-up done by a rehabilitation doctor who has technical and human resources that can guarantee an appropriate training and a medical and technical follow-up for this type of device.

Instructions for use

The prosthesis equipped with the Axon-Hook was designed for activities of daily living. It is indicated for carrying out manual activities for professional or leisure purposes. It must be recharged daily. The limits of use of the prosthesis are described in the user manual.

## Contraindications

No contraindication.

## Manufacturer and distribution

Axon-hook is manufactured by the Otto Bock Healthcare and commercialized in France by Otto Bock France. This device is sold exclusively to prosthetists and rehabilitation centers specialized in orthopedic devices.

## Identification et traceability

Identification et traceability of the device

Each Axon-hook is identified by its reference « 8E600 » as well as with a unique serial number. Settings require the use of the software Axon-Soft 560X500.

Identification and traceability of the device during the clinical trial

Each Axon-hook used for the clinical trial will be labelled « Axon-Hook Study – Device under investigation ». Its traceability will be assured by its serial number.

# Justification of the design of the clinical trial

## Description of the existing care and its limits

The Axon-Hook device is not currently supported by the national health system.

## Results of available trials relevant to the study

There are no studies specific to the Axon-Hook.

# Risks and benefits of the medical device under investigation and of the clinical trial

## Expected clinical benefits

Thanks to the technical specificities of Axon-Hook and more particularly to its adjustable wrist and lockable in flexion / extension and to the small size of the device, it is expected that shoulder abduction is limited when gripping objects and that it be facilitated. Decreasing compensatory movements when handling objects should decrease fatigue and encourage daily use of the prosthesis.

## Risks

Axon-Hook is a CE marked Class I medical device. Its use in the context of the investigation will comply with the conditions defined for CE marking. Axon-Hook does not present any particular risk.

## Steps taken to control or mitigate risks

Participants to the study will be informed of the conditions of use of the device orally and in writing when the device will be fitted. The participant will benefit from at least one rehabilitation session by a member of the therapeutic team in order to integrate the functionalities of this device and to have a good control of it.

## Justification of the benefit/risk ratio

With regards to the low risks mentioned above, the study could highlight the improvement of the service provided by the Axon-Hook and justify its reimbursement for patients.

# Objectives and hypotheses of the clinical trial

## Primary and secondary objectives

### Primary objective

The primary objective is to compare shoulder abduction when moving objects with the Axon-Hook and the Greifer in trans radial-ulnar amputees using a myoelectric prosthesis.

### Secondary objectives

Secondary objectives are to compare the percentage of time spent with shoulder abduction greater than 60° during the Box and Blocks test, manual dexterity and satisfaction with the Axon-Hook and the Greifer in trans radial-ulnar amputees using a myoelectric prosthesis.

## Primary and secondary hypothesis

### Primary hypothesis

The primary hypothesis is that the Axon-Hook reduces compensatory movements when moving objects, in particular because of its flexible and orientable wrist. These compensatory movements are measurable in a motion analysis laboratory through the average shoulder abduction during cubes displacements at the Box and Blocks test, when compared to the Greifer.

### Secondary hypothesis

Secondary hypotheses are that the Axon-Hook reduces the time spent with shoulder abduction greater than 60 ° during the Box and Blocks test and increases satisfaction, compared to the Greifer. However, no significant difference is expected in terms of manual dexterity with the Axon-hook and the Greifer.

1. **Design of the clinical investigation**
   1. **Primary and secondary outcome measurements**
      1. **Primary outcome measurement**

Average shoulder abduction during the Box and Blocks test

The primary outcome measurement is the average shoulder abduction when doing the Bock and Blocks manual dexterity test, over a period of 60 seconds. This measurement is obtained from the analysis of data acquired in a movement laboratory.

The Box and Blocks test was chosen because it is widely cited in the literature for the evaluation of subjects with upper limb amputations. For this test, the subject is seated at a table and faces a rectangular box which is divided into two compartments of equal dimensions by means of a partition. The test involves moving cubes one at a time from one compartment to the other one in the box. The greater the number of cubes moved, the better the manual dexterity. This repetition of displacing objects is consistent with the tasks that can be performed with a myoelectric hook and is compatible with movements acquisitions in a laboratory.

During the first movement acquisition in the laboratory, the subject is first assessed on the contralateral side to the side under investigation (i.e. the healthy side in the case of unilateral amputation). Acquisitions are then made with the Axon-Hook and the Greifer, in the order defined by the randomization.

- - 1. **Secondary outcome measurements**

Percentage of time spent with shoulder abduction greater than or equal to 60°

One of the secondary outcome measurement is the percentage of time spent with a shoulder abduction greater than or equal to 60 ° when doing the Bock and Blocks manual dexterity test, that is to say over a period of 60 seconds. This measurement is obtained from the analysis of data acquired in a movement laboratory.

Box and Blocks test

The criterion for evaluating manual dexterity is the number of cubes the subject displaced in one minute when doing the Box and Blocks test.

ESAT satisfaction questionnaire

The criterion for evaluating the user satisfaction is the score obtained at the ESAT self-questionnaire, the French version of the QUEST validated for amputees. The ESAT questionnaire contains a part for the evaluation of the technology of the device on 8 items and a part for the evaluation of services around the device on 4 items. It allows the patient to express himself on the criteria that are most important to him. It also makes it possible to calculate an average satisfaction value on a scale going for 0 to 5, and a distribution of patients "Not satisfied at all ", "Not very satisfied", "more or less satisfied", "Quite satisfied" or "Very satisfied" ".

The final ESAT score is the average obtained over the 12 items.

- 1. **Experimental plan**

This is a non-blinded, randomized, crossover, controlled superiority trial where each patient is his own control.

Study design

Inclusion

GREIFER

Fitting and rehabilitation

Randomization

AXON-HOOK

Fitting and rehabilitation

GREIFER

Fitting and rehabilitation

GREIFER

Evaluation

GREIFER

Evaluation

AXON-HOOK

Evaluation

T1 + 2 weeks.

T0

T1

T1

AXON-HOOK

Fitting and rehabilitation

AXON-HOOK

Evaluation

T1 + 2 weeks

T2

T2 + 2 weeks

T2

T2 + 2 weeks

Non-blinded trial

The study cannot be done in double blind or single blind. Since the device is fitted at the distal end of the prosthesis and has a gripping role, it is not possible to hide it from the patient or the investigator throughout the study.

Follow-up duration

Due to the ease of use of the hooks, the follow-up duration is a minimum of two weeks with each device, with a tolerance of two additional weeks to be able to set the date of the assessment visit. When changing the device, the rehabilitation session(s) will also act as “wash out”.

Fitting

In order to assess only the hook effect, for the same patient, the choice of passive or motorized pronosupination should be the same for both devices.

Rehabilitation

Subjects included in the study are regular users of and have a good control of their myoelectric prosthesis. The objective of the rehabilitation is therefore to familiarize the subject with the technical specificities and the use of the device. Thus, the person follows at least one rehabilitation session with each device. The number of sessions can however be adapted according to the individual needs of each person.

Adverse events

In the event of a technical failure of the prosthesis or of an event preventing from the use of the device for several days, the date of the end of the monitoring period will be postponed for as many days in order to respect the two weeks of monitoring planned.

In the event of technical failure of the evaluated device, it will be replaced by a strictly identical device with strictly the same operating settings.

- 1. **Medical device under investigation and comparator**

Medical device under investigation: Axon-Hook Otto Bock

Comparator: Greifer Otto Bock

- - 1. **Description of the exposure to the medical device under investigation and to the comparator**

Axon-Hook and Greifer are non-morphological tools used as an alternative to the myoelectric hand to perform certain activities. The frequency and duration of use of the devices therefore vary from one subject to another, depending on the subject's professional or life project.

- - 1. **Justification of the choice of the comparator.**

The comparator is the Greifer, which has been registered to the LPPR under brand name in Otto Bock's Myobock system since 2007. This system, widely prescribed in France, logically induces the Greifer as the study comparator.

- 1. **Study population**
     1. **Inclusion criteria**
- persons with upper limb amputation at the trans radio-ulnar level
- persons with acquired or congenital amputation
- persons who regularly use a myoelectric prosthesis and who have a good control of it
- persons whose residual limb is stabilized and who are at least six months away from the amputation
- persons whose professional activity or life project justifies or could justify the use of a myoelectric hook
- persons who have given their free and informed written consent
  - 1. **Exclusion criteria**
- persons under 18
- pregnant women
- persons in emergency situations
- persons unable to personally give their consent
- persons who are psychically or linguistically unable to understand the instructions for taking the research tests
- persons not available to comply with the entire study protocol

## Practical flow of the clinical investigation

### Recruitment methods

Patient recruitment will take place over an inclusion period during which all patients followed by the center and meeting the inclusion criteria will be offered to participate in the study.

### Information and consent collection procedures

During the inclusion visit, the investigating doctor provides the patient with oral and written information (appendix 1) explaining the purpose, progress and risks associated with the study. He makes sure that the person meets the inclusion criteria and he also makes sure that this information is properly understood by his patient.

The patient can benefit from a cooling-off period before giving his consent. In the event that he wishes to benefit from this cooling-off period, the doctor will schedule a new inclusion visit to obtain the patient's consent in writing using the form (appendix 2). He keeps an original in the patient record and gives a copy to the patient.

Anyone who made the inclusion visit, including any topics not included, will be described.

### Randomization methods

Randomization is done by means of sealed envelopes each containing an order of evaluation of the devices: 4 envelopes mention the test order "Axon-Hook then Greifer" and 4 other envelopes mention the test order " Greifer then Axon-Hook ”.

During the inclusion visit and after obtaining the patient's consent to participate, the investigator draws lots an envelope in order to define the order of testing of the devices.

Randomization is done by means of sealed envelopes each containing an order of evaluation of the devices: 4 envelopes mention the test order "Axon-Hook then Greifer" and 4 other envelopes mention the test order "La randomisation se fait au moyen d'enveloppes scellées contenant chacune un ordre d'évaluation des appareils: 4 enveloppes mentionnent l'ordre de test "Axon-Hook puis Greifer" et 4 autres enveloppes mentionnent l'ordre de test "Randomization is made by means of sealed envelopes each containing one of the devices evaluation order: 4 envelopes mention the test order "Axon Hook then Greifer" and four other envelopes mention the test command "La randomisation se fait au moyen d'enveloppes scellées contenant chacune l'un des ordres d'évaluation des dispositifs: 4 enveloppes mentionnent l'ordre de test "Axon Hook puis Greifer" et quatre autres enveloppes mentionnent la commande de test "

Impossible de charger les résultats complets

Réessayer

Nouvel essai…

Nouvel essai…

Greifer then Axon-Hook ”.

Greifer puis Axon-Hook ».

Greifer then Axon-Hook ".

Greifer puis Axon-Hook ".

Impossible de charger les résultats complets

Réessayer

Nouvel essai…

Nouvel essai…

During the inclusion visit and after obtaining the patient's consent to participate, the investigator draws lots an envelope in order to define the order of testing of the devices.

Lors de la visite d'inclusion et après avoir obtenu le consentement du patient à participer, l'investigateur tire au sort une enveloppe afin de définir l'ordre de test des dispositifs.

At the inclusion visit and after obtaining the patient's participation agreement, the fire investigator draw an envelope to define the order of testing the devices.

Lors de la visite d'inclusion et après avoir obtenu l'accord de participation du patient, l'enquêteur incendie dessine une enveloppe pour définir l'ordre de test des dispositifs.

Impossible de charger les résultats complets

Réessayer

Nouvel essai…

Nouvel essai…

### Prosthetic devices and rehabilitation program

Prosthetic devices

The fitting is performed by an orthoprosthetist authorized by Otto Bock to place the Axon-Hook device.

This ensures the comfort and proper adaptation of the prostheses during the trial and makes personalized adjustments for the patient.

This ensures the comfort and proper adaptation of the prostheses during the trial and makes personalized adjustments for the patient.

Cela garantit le confort et la bonne adaptation des prothèses pendant l'essai et permet des ajustements personnalisés pour le patient.

This ensures comfort and the suitability of implants tested and makes the custom settings for the patient.

Cela garantit le confort et l'adéquation des implants testés et effectue les réglages personnalisés pour le patient.

Impossible de charger les résultats complets

Réessayer

Nouvel essai…

Nouvel essai…

In order to assess only the clamping effect, for the same patient, the choice of passive or motorized wrist rotation is the same for both devices.

In order to assess only the clamping effect, for the same patient, the choice of passive or motorized pronosupination is the same for both devices.

Afin d'évaluer uniquement l'effet clampage, pour un même patient, le choix de la pronosupination passive ou motorisée est le même pour les deux dispositifs.

In order to evaluate only the effect clamp for the same patient, the choice of a passive or motorized pronosupination is the same for both devices.

Afin d'évaluer uniquement l'effet clamp pour un même patient, le choix d'une pronosupination passive ou motorisée est le même pour les deux appareils.

Impossible de charger les résultats complets

Réessayer

Nouvel essai…

Nouvel essai…

The sockets are identical for each tested prosthesis.

Rehabilitation program

In the week following each fitting, the patient follows at least one rehabilitation session.

In the week following each fitting, the patient follows at least one rehabilitation session.

Dans la semaine suivant chaque appareillage, le patient suit au moins une séance de rééducation.

In the week following each apparatus, the patient is at least one rehabilitation session.

Dans la semaine suivant chaque appareil, le patient est au moins une séance de rééducation.

Impossible de charger les résultats complets

Réessayer

Nouvel essai…

Nouvel essai…

The purpose of this is to make sure that he is familiar with the functionalities of each device and that he has perfect control over them.

The purpose of this is to make sure that he is familiar with the functionalities of each device and that he has perfect control over them.

Le but est de s'assurer qu'il est familiarisé avec les fonctionnalités de chaque appareil et qu'il a une parfaite maîtrise de celles-ci.

It aims to ensure that it knows the capabilities of each device and that he has complete control.

Il vise à s'assurer qu'il connaît les capacités de chaque appareil et qu'il a un contrôle total.

Impossible de charger les résultats complets

Réessayer

Nouvel essai…

Nouvel essai…

If necessary, additional sessions are offered.

If necessary, additional sessions are offered.

Si nécessaire, des sessions supplémentaires sont proposées.

Impossible de charger les résultats complets

Réessayer

Nouvel essai…

Nouvel essai…

The total number of sessions performed is recorded by the investigator in the observation notebook.

The total number of sessions performed is recorded by the investigator in the observation book.

Le nombre total de séances effectuées est enregistré par l'enquêteur dans le livre d'observation.

The total number of sessions is carried forward by the investigator on the case report.

Le nombre total de séances est reporté par l'enquêteur sur le rapport de cas.

Impossible de charger les résultats complets

Réessayer

Nouvel essai…

Nouvel essai…

### Equipment required for the clinical investigation

Prosthetic devices

The orthoprosthetist must have the Paula and AxonSoft 2 software available to adjust the prostheses with the Greifer and Axon-Hook.

Rehabilitation program

No specific equipment other than that usually used by rehabilitation teams is necessary as part of this clinical investigation.

Motion analysis

Shoulder abduction amplitudes are analyzed in a motion analysis laboratory. The system chosen for this functional exploration is the VICON® 3-dimensional motion analysis system (© 2007 Vicon Motion Systems Limited) which includes 2 Basler cameras for capturing videos from 2 different planes and 9 opto-electronic cameras which use infrared light to record the trajectories of markers positioned on the following points:

use infrared light to record the trajectories of markers positioned on the following points:

utiliser la lumière infrarouge pour enregistrer les trajectoires des marqueurs positionnés sur les points suivants:

use light infrared markers to record the trajectories positioned on the following:

utilisez des marqueurs infrarouges légers pour enregistrer les trajectoires positionnées sur les éléments suivants:

Impossible de charger les résultats complets

Réessayer

Nouvel essai…

Nouvel essai…

| Label | Marker Name | Position |
| --- | --- | --- |
| Trunk markers | | |
| LFHD / RFHD | Front of the head | Right and left front of the head at the temples |
| LBHD / RBHD | Back of the head | Right and left back of the head  Symmetrical in relation to the markers of the forward head |
| C7 | C7 | Spinous process of the 7th cervical vertebra |
| RBAK | Right Back | Right homoplate |
| T10 | T10 | Spinous process of the 10th thoracic vertebra |
| CLAV | Clavicle | Jugular notch: meeting point between the clavicle and the sternum |
| STRN | Sternum | Xyphoid appendix |
| LASI / RASI | EIAS | Iliac Antero-Superior Spines |
| SACR | Sacrum | Regarding the dimples in the sacrum |
| Markers on each upper limb (L=Left ; R=Right) | | |
| LSHO / RSHO | Shoulder | Acromioclavicular junction |
| LUPA / RUPA | Arm marker | Lateral part of the arm |
| LELB / RELB | Elbow | Lateral epicondyle of the elbow, near the articular axis |
| LFRM / RFRM | Forearm | Lateral part of the forearm |
| LWRA / RWRA | Wrist A Marker | Radial styloid, near the articular axis |
| LWRB / RWRB | Wrist B Marker | Ulnar styloid, near the articular axis |
| LFIN / RFIN | Finger | Below the third metacarpal |
| Markers on furniture | | |
| TABLE 1 / TABLE 2 | Table | 4 markers : one at each corner of the table |
| FR 1 / FR 2 | Stool | 2 markers at the back of the stool, at seat level |
| BOITE 1 / BOITE 2 / BOITE 3 | Box and Blocks | 3 markers : two on the front right and left corners of the Box and Blocks (BOITE 1 & 3), one on the front edge of the separation of the two compartments (BOITE 2) |

NEXUS® software, developed by the VICON® Company, analyzes data from infrared cameras and reconstructs the position of each marker in three dimensions of space.

NEXUS® software, developed by the VICON® company, analyzes data from infrared cameras and reconstructs the position of each marker in three dimensions of space.

Le logiciel NEXUS®, développé par la société VICON®, analyse les données des caméras infrarouges et reconstitue la position de chaque marqueur dans trois dimensions de l'espace.

NEXUS® software developed by the Vicon® company, analysis data of infrared cameras and reconstructs the position of each marker in three-dimensional space.

Le logiciel NEXUS® développé par la société Vicon®, analyse les données des caméras infrarouges et reconstitue la position de chaque marqueur dans un espace tridimensionnel.

Impossible de charger les résultats complets

Réessayer

Nouvel essai…

Nouvel essai…

It extracts the data recorded in spreadsheet form at a rate of 100 images per second (100Hz).

It extracts the data recorded in spreadsheet form at a rate of 100 images per second (100Hz).

Il extrait les données enregistrées sous forme de tableur à un taux de 100 images par seconde (100 Hz).

It allows to extract the data stored in spreadsheet format at 100 frames per second (100Hz).

Il permet d'extraire les données stockées au format tableur à 100 images par seconde (100Hz).

Impossible de charger les résultats complets

Réessayer

Nouvel essai…

Nouvel essai…

The shoulder abduction values correspond to anatomical measurements calculated according to the Plug-In Gait® model.

The shoulder abduction values correspond to anatomical measurements calculated according to the Plug-In Gait® model.

Les valeurs d'abduction d'épaule correspondent à des mesures anatomiques calculées selon le modèle Plug-In Gait®.

The values abduction of the shoulder correspond to the anatomical measures calculated according to the Plug-In Gait® model.

Les valeurs d'abduction de l'épaule correspondent aux mesures anatomiques calculées selon le modèle Plug-In Gait®.

Impossible de charger les résultats complets

Réessayer

Nouvel essai…

Nouvel essai…

Box and blocks Test

The motion analysis and the evaluation of manual dexterity require having the Box and Blocks tool which is a standardized test.

The analysis of movement and the evaluation of manual dexterity require having the Box and Blocks tool which is a standardized test.

L'analyse du mouvement et l'évaluation de la dextérité manuelle nécessitent de disposer de l'outil Box and Blocks qui est un test standardisé.

The motion analysis and evaluation of manual dexterity required to have the Box and Blocks tool is a standardized test.

L'analyse du mouvement et l'évaluation de la dextérité manuelle requise pour disposer de l'outil Boîte et blocs est un test standardisé.

Impossible de charger les résultats complets

Réessayer

Nouvel essai…

Nouvel essai…

This test consists of moving a maximum of blocks in one minute from one compartment to another of a box positioned on the edge of a table of standard height, the subject being seated on a chair of standard height, facing the box. The assessment conditions are similar regardless of the device assessed.

The assessment conditions are similar regardless of the device assessed.

Les conditions d'évaluation sont similaires quel que soit l'appareil évalué.

The evaluation conditions are similar regardless of the evaluated device.

Les conditions d'évaluation sont similaires quel que soit l'appareil évalué.

Impossible de charger les résultats complets

Réessayer

Nouvel essai…

Nouvel essai…

This test consists of moving a maximum of cubes in one minute from one compartment to another of a box positioned on the edge of a table of standard height, the subject being seated on a chair of standard height, facing the

Ce test consiste à déplacer un maximum de cubes en une minute d'un compartiment à l'autre d'une boîte positionnée sur le bord d'une table de hauteur standard, le sujet étant assis sur une chaise de hauteur standard, face au

This test involves moving a maximum of cubes in a minute from one compartment to the other of a box positioned on the edge of a standard height table with the subject sitting on a standard chair height, facing the

Ce test consiste à déplacer un maximum de cubes en une minute d'un compartiment à l'autre d'une boîte positionnée sur le bord d'une table de hauteur standard avec le sujet assis sur une hauteur de chaise standard, face au

Impossible de charger les résultats complets

Réessayer

Nouvel essai…

Nouvel essai…

box.

...

Impossible de charger les résultats complets

Réessayer

Nouvel essai…

Nouvel essai…

### Discontinuation of study or premature termination of follow-up

Les sujets peuvent retirer leur consentement et demander à sortir de l’étude a n’importe quel moment et quelle qu’en soit la raison. En cas de sortie prématurée, l’investigateur documente les raisons de façon aussi complète que possible. L’investigateur peut interrompre temporairement ou définitivement la participation d’un sujet à l’étude pour toute raison qui servirait au mieux les intérêts du sujet, en particulier en cas d’évènements indésirables graves. En cas de sujet perdu de vue, l’investigateur doit mettre tout en œuvre pour reprendre contact avec la personne. Apres trois relances téléphoniques hebdomadaires, le sujet est enregistré comme perdu de vue. L’investigateur renseigne dans le cahier d’observation électronique la raison de sortie d’étude ou d’arrêt prématuré du suivi.

### Subjects replacement procedures, if applicable

In the event of withdrawal from the study or premature discontinuation of the follow-up of a large number of subjects, the sponsor may request that new subjects meeting the inclusion criteria be included in the study.

In the event of withdrawal from the study or premature discontinuation of the follow-up of a large number of subjects, the sponsor may request that new subjects meeting the inclusion criteria be included in the study.

En cas de retrait de l'étude ou d'arrêt prématuré du suivi d'un grand nombre de sujets, le promoteur peut demander que de nouveaux sujets répondant aux critères d'inclusion soient inclus dans l'étude.

In case of study or early termination of output followed by a large number of subjects, the sponsor may request that new subjects meeting the inclusion criteria were included in the study.

En cas d'étude ou d'interruption prématurée de la production suivie d'un grand nombre de sujets, le promoteur peut demander que de nouveaux sujets répondant aux critères d'inclusion soient inclus dans l'étude.

Impossible de charger les résultats complets

Réessayer

Nouvel essai…

Nouvel essai…

### Chronology of visits

**Visit 1**

Patient Inclusion

- Patient Information

- Collect of consent

- Treatment Allocation:

Axon-Hook / Greifer Group

OR

Greifer / Axon-Hook Group

**Visit 2**

Axon-Hook fitting

Rehabilitation program

AXON-HOOK

(2 weeks)

GREIFER

(2 weeks)

GREIFER

(2 weeks)

AXON-HOOK

(2 weeks)

**Visit 3**

**(2 weeks after placement of the medical device)**

Axon-Hook Evaluation

- Test Box and Blocks (Movement Laboratory)

- auto-questionnaire ESAT

**Visit 4**

Greifer fitting

Rehabilitation program

**Visit 5**

**(2 weeks after placement of the medical device)**

Greifer Evaluation,

- Box and Blocks test (Movement Laboratory)

- auto-questionnaire ESAT

- patient preference

**Visit 2**

Greifer fitting

Rehabilitation program

**Visit 3**

**(2 weeks after placement of the medical device)**

Evaluation Greifer

- Test Box and Blocks (Movement Laboratory)

- auto-questionnaire ESAT

**Visit 4**

Axon-Hook fitting

Rehabilitation program

**Visit 5**

**(2 weeks after placement of the medical device)**

Axon-Hook Evaluation,

- Box and Blocks test (Movement Laboratory)

- auto-questionnaire ESAT

- patient preference

### Duration of participation for each subject and total duration of the clinical investigation

Considering approximately two weeks of testing for each device, the participation time for each subject is approximately 4 weeks. The total duration of the clinical investigation is estimated at five months.

Considering approximately two weeks of testing for each device, the participation time for each subject is approximately 4 weeks.

Compte tenu d'environ deux semaines de test pour chaque appareil, le temps de participation pour chaque sujet est d'environ 4 semaines.

Considering about two weeks to test each device, participation time for each subject is about 4 weeks.

En considérant environ deux semaines pour tester chaque appareil, le temps de participation pour chaque sujet est d'environ 4 semaines.

Impossible de charger les résultats complets

Réessayer

Nouvel essai…

Nouvel essai…

The total duration of the clinical investigation is estimated at five months.

La durée totale de l'investigation clinique est estimée à cinq mois.

Impossible de charger les résultats complets

Réessayer

Nouvel essai…

Nouvel essai…

## Management of adverse events

### Definitions

- **Adverse Events (AEv)**: any harmful manifestation occurring in a person who lends itself to biomedical research, whether or not this manifestation is linked to the research or to the experimental device to which this research relates.
- any harmful manifestation occurring in a person who lends itself to biomedical research, whether or not this manifestation is linked to the research or to the experimental device to which this research relates.
- toute manifestation néfaste survenant chez une personne qui se prête à la recherche biomédicale, que cette manifestation soit ou non liée à la recherche ou au dispositif expérimental auquel cette recherche porte.
- any untoward medical occurrence that occurs in a person who is suitable for biomedical research that this event is related or not to research or experimental device as subject of the research.
- tout événement médical fâcheux qui survient chez une personne apte à la recherche biomédicale que cet événement est lié ou non à la recherche ou au dispositif expérimental en tant que sujet de la recherche.
- Impossible de charger les résultats complets
- Réessayer
- Nouvel essai…
- Nouvel essai…
- **Serious Adverse Event (SAEv)** : severity is defined by one of the following findings:
  - Death
  - Involvement of the vital prognosis (immediate threat to life, at the time of the event, regardless of the consequences of corrective or palliative therapy)
  - Incapacity or significant/lasting handicap
  - Hospitalization
  - Extension of hospitalization
  - Congenital malformation / abnormality
  - o Congenital malformation / abnormality
  - o Malformation / anomalie congénitale
  - o malformation / birth defect
  - o malformation / anomalie congénitale
  - Impossible de charger les résultats complets
  - Réessayer
  - Nouvel essai…
  - Nouvel essai…
  - Potentially serious event (adverse clinical event or laboratory result of a serious nature or considered as such by the investigator)
- **Adverse Effect (AEf)**: any harmful and unwanted effect linked to an experimental device or any incident which could have caused this effect if an appropriate action had not been taken, in a person who is suitable for research or in the patient/user of the medical device.
- **Serious Adverse Effect (SAEf)**: serious adverse effect attributable to an experimental device.
- **Unexpected side effect**: effect of which the nature, severity, intensity or evolution does not agree with the information given in the instruction leaflet or the instructions for use when it is the subject of a CE marking, and in the protocol or the investigator brochure when it is not the subject of such a marking.
- **Accountability**: relationship between the AEv and the study device. The AEv linked to the experimental device will become an AE. The factors to be taken into account in determining accountability are:
  - the chronology of events,
  - the AEv disappearance when the device is removed and / or the reappearance in the event of a new installation,
  - the notion of antecedent of a similar event when using the device or a device of the same class,
  - the existence of another etiology.
- **Intensity**: the AEv intensity by the investigator according to the following classification:
  - mild grade 1 : AEv generally transient and without impact on normal activities,
  - medium grade 2 : AEv embarrassing enough to affect normal activities,
  - severe grade 3 : AEv significantly altering the normal course of the patient's activities, or disabling, or posing a threat to the patient's life.

Note: the intensity criterion should not be confused with the severity criterion which serves as a guide to define the reporting obligations.

### List of foreseeable adverse events and adverse effects expected from the device, their probable incidence, means of mitigation or treatment

Foreseeable adverse events:

Foreseeable adverse events:

Événements indésirables prévisibles:

predictable Adverse Events:

Événements indésirables prévisibles:

Impossible de charger les résultats complets

Réessayer

Nouvel essai…

Nouvel essai…

Impossible de charger les résultats complets

Réessayer

Nouvel essai…

Nouvel essai…

- Injury
- Disease
- Hospitalization
- Problem of prosthesis adaptation or adjustment
- Dysfunction of the prosthesis
- Failure of the prosthesis
- - Dysfunction of the prosthesis
- - Dysfonctionnement de la prothèse
- - Malfunction of the prosthesis
- - Dysfonctionnement de la prothèse
- Impossible de charger les résultats complets
- Réessayer
- Nouvel essai…
- Nouvel essai…
- Breakage of the prosthesis

Side effects expected from the device:

No adverse effects expected from the device

No adverse effects expected from the device

Aucun effet indésirable attendu de l'appareil

No adverse effects expected of the device

Aucun effet indésirable attendu de l'appareil

Impossible de charger les résultats complets

Réessayer

Nouvel essai…

Nouvel essai…

### Role of the investigator

Throughout the patient follow-up period, any AEv that occurs to a person in the trial should be followed until resolution, or until it is considered permanent. The occurrence of AEv should be sought by the investigator at each follow-up visit. Any SAEv, whether or not related to the test, must be documented in the form provided for this purpose in the observation book. Any change in the severity, the relationship with the medical device, the interventions required to treat the event or the course should be monitored and documented. Any SAEv , regardless of its causal relationship with the trial device must be declared to the sponsor in the form provided for this purpose in the observation book within 24 hours of its occurrence (or as soon as the investigating physician is aware of it). The initial declaration may be followed by additional relevant information within 8 days in the event of a fatal or life-threatening event and within 15 days in other cases.

Impossible de charger les résultats complets

Réessayer

Nouvel essai…

Nouvel essai…

### Role of the promoter

The promoter assesses the causality of AEv (all adverse events, for which the investigator or the sponsor considers that a causal relationship with the experimental device can be reasonably envisaged, are considered to be suspicions of adverse effects.

The sponsor assesses the causality of EvI (all adverse events, for which the investigator or the sponsor considers that a causal relationship with the experimental device can be reasonably envisaged, are considered to be suspicions of adverse effects.

Le promoteur évalue la causalité d'EvI (tous les événements indésirables, pour lesquels l'investigateur ou le promoteur considère qu'une relation causale avec le dispositif expérimental peut être raisonnablement envisagée, sont considérés comme des soupçons d'effets indésirables.

The proponent estimates causality Evi (all adverse events for which the investigator or the sponsor believes that a causal relationship with the experimental device can be reasonably expected are considered suspected adverse effects. If

Le promoteur estime la causalité Evi (tous les événements indésirables pour lesquels l'investigateur ou le promoteur estime qu'une relation causale avec le dispositif expérimental peut être raisonnablement prévisible sont considérés comme des effets indésirables suspectés.

Impossible de charger les résultats complets

Réessayer

Nouvel essai…

Nouvel essai…

(different evaluations of the sponsor and the investigator, the two opinions are mentioned on the declaration sent to the competent authority if this declaration is necessary),

(different evaluation of the sponsor and the investigator, the two opinions are mentioned on the declaration sent to the competent authority if this declaration is necessary),

(évaluation différente du promoteur et de l'investigateur, les deux avis sont mentionnés sur la déclaration adressée à l'autorité compétente si cette déclaration est nécessaire),

'different assessment of the sponsor and investigator, the two opinions are mentioned in the statement addressed to the competent authority that declaration is necessary)

`` évaluation différente du promoteur et de l'investigateur, les deux avis sont mentionnés dans la déclaration adressée à l'autorité compétente selon laquelle une déclaration est nécessaire)

Impossible de charger les résultats complets

Réessayer

Nouvel essai…

Nouvel essai…

Impossible de charger les résultats complets

Réessayer

Nouvel essai…

Nouvel essai…

The promoter declares to the ANSM and the CPP:

The sponsor declares to the ANSM and the CPP:

Le promoteur déclare à l'ANSM et au CPP:

The promoter said to ANSM and PPC:

Le promoteur a déclaré à l'ANSM et au PPC:

Impossible de charger les résultats complets

Réessayer

Nouvel essai…

Nouvel essai…

- all SAEv and unexpected events likely to be due to an medical device being investigated
- all SAEs and unexpected events likely to be due to an MD being investigated
- tous les EIG et événements inattendus susceptibles d'être dus à un DM faisant l'objet d'une enquête
- All SAEs and unexpected likely to be due to a DM subject of research
- Tous les EIG et imprévus susceptibles d'être dus à un sujet de recherche DM
- Impossible de charger les résultats complets
- Réessayer
- Nouvel essai…
- Nouvel essai…
- all SAEv that can be linked to the act of implementing the medical device

The regulatory declaration is made within a maximum period of:

- 7 calendar days in the case of unexpected serious events and SAEv that may be linked to the act of implementation and resulting in death or endangerment. In these cases, relevant additional information must be sought and transmitted within a further 8 days.

- 15 calendar days for all other unexpected and unexpected serious events and SAEv that may be linked to the act of implementing the medical device. Likewise, relevant additional information must be sought and transmitted within a further 15 days.

15 calendar days for all other unexpected and unexpected serious events and EvIG that may be linked to the act of implementing the medical device.

15 jours calendaires pour tous les autres événements graves imprévus et imprévus et EvIG pouvant être liés à l'acte de mise en œuvre du dispositif médical.

15 calendar days for all other unexpected serious unexpected événemenys evig and may be linked to implementing gesture of the medical device.

15 jours calendaires pour tous les autres événements imprévus graves et imprévus et pouvant être liés à la mise en œuvre du geste du dispositif médical.

Impossible de charger les résultats complets

Réessayer

Nouvel essai…

Nouvel essai…

Likewise, relevant additional information must be sought and transmitted within a further 15 days.

De même, des informations complémentaires pertinentes doivent être recherchées et transmises dans un délai de 15 jours supplémentaires.

Similarly additional relevant information must be sought and transmitted within a further period of 15 days.

De même, des informations complémentaires pertinentes doivent être recherchées et transmises dans un délai supplémentaire de 15 jours.

Impossible de charger les résultats complets

Réessayer

Nouvel essai…

Nouvel essai…

7 jours calendaires en cas de SAE et EVIG imprévus pouvant être liés à l'acte de mise en œuvre et entraînant la mort ou la mise en danger.

7 calendar days in the case of unexpected SAEs and evig can be linked to implementation of gesture and resulting in death or endangerment.

7 jours calendaires en cas d'EIG imprévus et evig peuvent être liés à la mise en œuvre du geste et entraînant la mort ou la mise en danger.

Impossible de charger les résultats complets

Réessayer

Nouvel essai…

Nouvel essai…

In these cases, relevant additional information must be sought and transmitted within a further 8 days.

Dans ces cas, des informations complémentaires pertinentes doivent être recherchées et transmises dans un délai supplémentaire de 8 jours.

In these cases, additional relevant information must be sought and transmitted within a further period of 8 days.

Dans ces cas, des informations complémentaires pertinentes doivent être recherchées et transmises dans un délai supplémentaire de 8 jours.

Impossible de charger les résultats complets

Réessayer

Nouvel essai…

Nouvel essai…

The adverse event observation notebooks for all AEv notified by the investigators may be sent to the ANSM, at its request.

## Monitoring plan

# Statistics

## Sample size

The calculation of the number of patients to be included is based on the expected difference on the primary endpoint, the average shoulder abduction when taking the Box and Blocks test. With the assumption of an average abduction difference of 30 ± 25 degrees, it is necessary to include 8 patients for a randomized cross-over trial. An improvement in the secondary endpoints is expected but not a significant difference. The number of patients is therefore only calculated on the basis of the primary endpoint.

The calculation of the number of patients to be included is based on the expected difference on the primary endpoint, the average shoulder abduction when taking the Box and Blocks test.

Le calcul du nombre de patients à inclure est basé sur la différence attendue sur le critère principal, l'abduction moyenne de l'épaule lors du test Box and Blocks.

The calculation of the number of patients to be included is based on the expected difference on the primary endpoint, the mean shoulder abduction when placing test Box and Blocks.

Le calcul du nombre de patients à inclure est basé sur la différence attendue sur le critère d'évaluation principal, l'abduction moyenne de l'épaule lors de la mise en place de la boîte et des blocs de test.

Impossible de charger les résultats complets

Réessayer

Nouvel essai…

Nouvel essai…

With the assumption of an average abduction difference of 30 ± 25 degrees, it is necessary to include 8 patients for a randomized cross-over trial.

En supposant une différence d'abduction moyenne de 30 ± 25 degrés, il est nécessaire d'inclure 8 patients pour un essai croisé randomisé.

With the assumption of an average difference of abduction of 30 ± 25 degrees it is necessary to include 8 patients for a randomized trial crossover.

En supposant une différence moyenne d'abduction de 30 ± 25 degrés, il est nécessaire d'inclure 8 patients pour un essai croisé randomisé.

Impossible de charger les résultats complets

Réessayer

Nouvel essai…

Nouvel essai…

An improvement in the secondary endpoints is expected but not a significant difference.

Une amélioration des paramètres secondaires est attendue mais pas de différence significative.

It is expected an improvement in secondary endpoints, but not a significant difference.

On s'attend à une amélioration des paramètres secondaires, mais pas à une différence significative.

Impossible de charger les résultats complets

Réessayer

Nouvel essai…

Nouvel essai…

The number of patients is therefore only calculated on the basis of the primary endpoint.

Le nombre de patients n'est donc calculé que sur la base du critère principal.

The number of patients is only calculated on the basis of the primary endpoint.

Le nombre de patients n'est calculé que sur la base du critère principal.

Impossible de charger les résultats complets

Réessayer

Nouvel essai…

Nouvel essai…

The calculation formula used in the case of a randomized cross-over trial is as follows :

**n = (1-ρ) * 2 * (σ²/Δ²) * (z(α/2)-z(1-β))²**

α, = 0.05

1-β = desired power

σ = standard deviation

Δ = expected difference

ρ = correlation coefficient between the responses of each patient to the two prostheses (if not known, generally estimated at 0.5)

The number of patients required is based on different hypotheses of reduction of the compensatory movements expected with the Axon-Hook forceps in comparison with the Greifer:

| Expected difference assumptions (reduction angle of mean shoulder abduction) | Common Standard deviation | Number of patients required |
| --- | --- | --- |
| 35 | 25 | 6 |
| 30 | 25 | 8 |
| 25 | 20 | 7 |

Given the very low prevalence of the target population, it is not possible to include more than 8 patients in this single-center study. It will be possible to include 8 patients.

Given the very low prevalence of the target population, it is not possible to include more than 8 patients in this single-center study.

Compte tenu de la très faible prévalence de la population cible, il n'est pas possible d'inclure plus de 8 patients dans cette étude monocentrique.

Impossible de charger les résultats complets

Réessayer

Nouvel essai…

Nouvel essai…

Impossible de charger les résultats complets

Réessayer

Nouvel essai…

Nouvel essai…

## Treatment of missing, unused or erroneous data

No replacement of missing data will be made. In the event of missing, illegible or inconsistent data, requests for additional information will be sent to the investigator concerned. The modifications will then be incorporated into the observation book by the investigating doctor, with mention of the date of amendment of the information and the signature of the investigator in the margin.

the modifications will then be incorporated into the observation book by the investigating doctor, with mention of the date of amendment of the information and the signature of the investigator in the margin.

les modifications seront ensuite incorporées dans le carnet d'observation par le médecin investigateur, avec mention de la date de modification des informations et de la signature de l'investigateur en marge.

the changes will be incorporated in the report form by the investigating physician, mentioning the date of amendment of the information and signature of the investigator in the margin.

les modifications seront incorporées dans le formulaire de rapport par le médecin investigateur, en mentionnant la date de modification des informations et la signature de l'investigateur dans la marge.

Impossible de charger les résultats complets

Réessayer

Nouvel essai…

Nouvel essai…

Impossible de charger les résultats complets

Réessayer

Nouvel essai…

Nouvel essai…

If, however, data is missing, the subject concerned will be removed from the analysis of the parameter concerned.

No replacement of missing data will be made.

Aucun remplacement des données manquantes ne sera effectué.

Impossible de charger les résultats complets

Réessayer

Nouvel essai…

Nouvel essai…

In the event of missing, illegible or inconsistent data, requests for additional information will be sent to the investigator concerned.

En cas de données manquantes, illisibles ou incohérentes, des demandes d'informations complémentaires seront adressées à l'enquêteur concerné.

If missing, illegible or inconsistent requests for additional information will be sent to the investigator concerned.

En cas d'absence, de demandes d'informations complémentaires illisibles ou incohérentes, seront adressées à l'enquêteur concerné.

Impossible de charger les résultats complets

Réessayer

Nouvel essai…

Nouvel essai…

;

...

Impossible de charger les résultats complets

Réessayer

Nouvel essai…

Nouvel essai…

If, however, data is missing, the subject concerned will be removed from the analysis of the parameter concerned.

Si toutefois des données manquent, le sujet concerné sera retiré de l'analyse du paramètre concerné.

If, however, data is missing, the subject concerned will be removed from the analysis parameter.

Si toutefois des données manquent, le sujet concerné sera supprimé du paramètre d'analyse.

Impossible de charger les résultats complets

Réessayer

Nouvel essai…

Nouvel essai…

## Analysis populations

An analysis on the intention-to-treat (ITT) population will be performed, then on the per-protocol (PP) population. The ITT population is defined by all the patients randomized to their randomization group, whatever treatment they actually received or whatever their fate in the study.

The ITT population is defined by all the patients randomized to their randomization group, whatever treatment they actually received or whatever their fate in the study.

La population ITT est définie par l'ensemble des patients randomisés dans leur groupe de randomisation, quel que soit le traitement qu'ils ont réellement reçu ou quel que soit leur sort dans l'étude.

The ITT population is defined as all randomized patients in their randomization group, regardless of the treatment they actually received or whatever their fate in the study.

La population ITT est définie comme l'ensemble des patients randomisés dans leur groupe de randomisation, quel que soit le traitement qu'ils ont réellement reçu ou quel que soit leur sort dans l'étude.

Impossible de charger les résultats complets

Réessayer

Nouvel essai…

Nouvel essai…

Impossible de charger les résultats complets

Réessayer

Nouvel essai…

Nouvel essai…

The PP population is defined by all the patients of the ITT population without deviation defined as major.

An analysis on the intention-to-treat (ITT) population will be performed, then on the per-protocol (PP) population.

Une analyse sur la population en intention de traiter (ITT) sera réalisée, puis sur la population per protocole (PP).

An analysis of the intent-to-treat (ITT) will be performed, then the per-protocol (PP).

Une analyse de l'intention de traiter (ITT) sera réalisée, puis le per protocole (PP).

Impossible de charger les résultats complets

Réessayer

Nouvel essai…

Nouvel essai…

The PP population is defined by all the patients of the ITT population without deviation defined as major.

La population PP est définie par tous les patients de la population ITT sans écart défini comme majeur.

The PP population is defined as all patients in the ITT population defined as no deviations.

La population PP est définie comme l'ensemble des patients de la population ITT définie comme aucun écart.

Impossible de charger les résultats complets

Réessayer

Nouvel essai…

Nouvel essai…

## Statistical analyzes

Patient characteristics

The quantitative variables will be summarized by the usual descriptive statistics (mean, standard deviation, median, minimum and maximum, 1st and 3rd quartile). Qualitative variables will be described by numbers and frequencies. 95% confidence intervals will be presented where relevant. The characteristics of the patients at inclusion will be presented and compared in each group in order to check the initial comparability of the groups. The characteristics of patients not included and those of those lost to follow-up will be studied.

The quantitative variables will be summarized by the usual descriptive statistics (mean, standard deviation, median, minimum and maximum, 1st and 3rd quartile).

Les variables quantitatives seront résumées par les statistiques descriptives habituelles (moyenne, écart-type, médiane, minimum et maximum, 1er et 3ème quartile).

Quantitative variables will be summarized by the usual descriptive statistics (mean, standard deviation, median, minimum and maximum, 1st and 3rd quartile).

Les variables quantitatives seront résumées par les statistiques descriptives habituelles (moyenne, écart-type, médiane, minimum et maximum, 1er et 3ème quartile).

Impossible de charger les résultats complets

Réessayer

Nouvel essai…

Nouvel essai…

Qualitative variables will be described by numbers and frequencies.

Les variables qualitatives seront décrites par des nombres et des fréquences.

The variables are described by the numbers and frequencies.

Les variables sont décrites par les nombres et les fréquences.

Impossible de charger les résultats complets

Réessayer

Nouvel essai…

Nouvel essai…

95% confidence intervals will be presented where relevant.

Des intervalles de confiance à 95% seront présentés le cas échéant.

The 95% confidence intervals will be presented when this is relevant.

Les intervalles de confiance à 95% seront présentés lorsque cela est pertinent.

Impossible de charger les résultats complets

Réessayer

Nouvel essai…

Nouvel essai…

The characteristics of the patients at inclusion will be presented and compared in each group in order to check the initial comparability of the groups.

Les caractéristiques des patients à l'inclusion seront présentées et comparées dans chaque groupe afin de vérifier la comparabilité initiale des groupes.

Patient characteristics at baseline are presented and compared in each group to verify the initial group comparability.

Les caractéristiques des patients au départ sont présentées et comparées dans chaque groupe pour vérifier la comparabilité initiale du groupe.

Impossible de charger les résultats complets

Réessayer

Nouvel essai…

Nouvel essai…

The characteristics of patients not included and those of those lost to follow-up will be studied.

Les caractéristiques des patients non inclus et celles des perdus de vue seront étudiées.

The characteristics of patients not included and those of view will be considered lost.

Les caractéristiques des patients non inclus et ceux de vue seront considérés comme perdus.

Impossible de charger les résultats complets

Réessayer

Nouvel essai…

Nouvel essai…

Analysis of primary and secondary endpoints

The study being crossed, the carry-over effect will be tested in order to test the interaction between the two prostheses using a paired Student's test.

The study being crossed, the carry-over effect will be tested in order to test the interaction between the two prostheses using a paired Student's test.

L'étude étant croisée, l'effet de report sera testé afin de tester l'interaction entre les deux prothèses à l'aide d'un test de Student apparié.

The study is cross, the carry-over effect will be tested to test the interaction between the two prostheses using a paired t test.

L'étude est croisée, l'effet de report sera testé pour tester l'interaction entre les deux prothèses à l'aide d'un test t apparié.

Impossible de charger les résultats complets

Réessayer

Nouvel essai…

Nouvel essai…

If the effect is significant, only the first period will be analyzed using a Student test or the nonparametric Wilcoxon test if the assumptions of normality and equality of variances are not verified.

If the effect is significant, only the first period will be analyzed using a Student test or the nonparametric Wilcoxon test if the assumptions of normality and equality of variances are not verified.

Si l'effet est significatif, seule la première période sera analysée à l'aide d'un test de Student ou du test non paramétrique de Wilcoxon si les hypothèses de normalité et d'égalité des variances ne sont pas vérifiées.

If the effect is significant, only the first period will be analyzed using Student's t test or the nonparametric Wilcoxon test if the assumptions of normality and equality of variances are not checked.

Si l'effet est significatif, seule la première période sera analysée à l'aide du test t de Student ou du test non paramétrique de Wilcoxon si les hypothèses de normalité et d'égalité des variances ne sont pas vérifiées.

Impossible de charger les résultats complets

Réessayer

Nouvel essai…

Nouvel essai…

Impossible de charger les résultats complets

Réessayer

Nouvel essai…

Nouvel essai…

If the effect is not significant, the period effect and the prosthesis effect can then be tested using a paired Student's test.

If the effect is not significant, the period effect and the prosthesis effect can then be tested using a paired Student's test.

Si l'effet n'est pas significatif, l'effet de période et l'effet de prothèse peuvent alors être testés à l'aide d'un test de Student apparié.

If the effect is not significant, the period effect and the effect prosthesis can then be tested using a paired t test.

Si l'effet n'est pas significatif, l'effet de période et l'effet prothèse peuvent alors être testés en utilisant un test t apparié.

Impossible de charger les résultats complets

Réessayer

Nouvel essai…

Nouvel essai…

Tolerance analysis

All the adverse events will be described in the analysis populations and for each of the two Axon-Hook and Greifer prostheses. Likewise, a summary of adverse events possibly related to the prosthesis and a summary of serious adverse events will be presented

All the adverse events will be described in the analysis populations and for each of the two Axon-Hook and Greifer prostheses.

Tous les événements indésirables seront décrits dans les populations d'analyse et pour chacune des deux prothèses Axon-Hook et Greifer.

All adverse events will be described in populations and analysis for both Axon Hook prostheses Greifer.

Tous les événements indésirables seront décrits dans les populations et l'analyse des deux prothèses Axon Hook Greifer.

Impossible de charger les résultats complets

Réessayer

Nouvel essai…

Nouvel essai…

# Quality control and assurance

## Promoter

Otto Bock is ISO 9001 and 13485 certified. The documents attached to the study are part of the company's quality system.

## Data collection

Data collection in the observation book is carried out by the investigating doctor. At the end of the follow-up of each patient, the investigating doctor dates, signs and affixes the center's stamp on the observation notebook to certify the accuracy of the information mentioned therein. He then sends a copy of the observation notebook to the promoter for analysis of the results and keeps the original in the study investigation file.

Data collection in the observation book is carried out by the investigating doctor.

La collecte des données dans le livre d'observation est effectuée par le médecin investigateur.

The collection of data on the case report is provided by the investigating physician.

La collecte des données sur le rapport de cas est assurée par le médecin investigateur.

Impossible de charger les résultats complets

Réessayer

Nouvel essai…

Nouvel essai…

At the end of the follow-up of each patient, the investigating doctor dates, signs and affixes the center's stamp on the observation booklet to certify the accuracy of the information mentioned therein.

A l'issue du suivi de chaque patient, le médecin investigateur date, signe et appose le cachet du centre sur le livret d'observation pour certifier l'exactitude des informations qui y sont mentionnées.

At the end of follow-up of each patient, the investigator time doctor, sign and affix the seal of the center of the case report to attest the accuracy of the information mentioned therein.

A l'issue du suivi de chaque patient, l'investigateur chronomètre le médecin, signe et appose le sceau du centre du rapport de cas pour attester de l'exactitude des informations qui y sont mentionnées.

Impossible de charger les résultats complets

Réessayer

Nouvel essai…

Nouvel essai…

He then sends a copy of the observation booklet to the sponsor for analysis of the results and keeps the original in the study investigation file.

Il envoie ensuite une copie du livret d'observation au promoteur pour analyse des résultats et conserve l'original dans le dossier d'investigation de l'étude.

It then transmits a copy of report form the promoter for analysis of the results and stores the original in the investigation of the study folder.

Il transmet ensuite une copie du rapport du promoteur pour analyse des résultats et stocke l'original dans le dossier d'investigation du dossier d'étude.

Impossible de charger les résultats complets

Réessayer

Nouvel essai…

Nouvel essai…

# Data processing

## CNIL

Data processing will be carried out in accordance with Reference Methodology No. MR-001, the request form of which will be sent to the « Commission Nationale de l’Informatique et des libertés » (CNIL) in France for approval.

## Data collected

The data is collected through a paper observation notebook.

| Data collected by the investigator | Inclusion Visit | Axon-Hook Evaluation Visit | Greifer Evaluation Visit |
| --- | --- | --- | --- |
| Patient characteristics | X |  |  |
| Informed patient consent | X |  |  |
| Mean shoulder abduction on the Box and Blocks test session |  | X | X |
| Percentage of time spent with a shoulder abduction greater than or equal to 60 ° on the Box and blocks test session |  | X | X |
| Box and Blocks Test |  | X | X |
| ESAT Questionnaire |  | X | X |
| Patient preference (after evaluation of the last device)Patient preference (after evaluation of the last device)Préférence du patient (après évaluation du dernier appareil)  Impossible de charger les résultats complets  Réessayer  Nouvel essai…  Nouvel essai… |  | X | X |

Data collected by the investigator during the inclusion visit:

- Patient initials
- Date of inclusion visit
- Sex : man / woman
- Age
- Height (cm)
- Weight (Kg)
- Professional activity: student / retired / without professional activity / in professional activity (specify)
- Professional activity: student / retired / without professional activity / in professional activity (specify)
- Activité professionnelle: étudiant / retraité / sans activité professionnelle / en activité professionnelle (préciser)
- Occupation: Student / Retired / without professional / vocational activity activity (specify)
- Profession: étudiant / retraité / sans activité professionnelle / professionnelle (préciser)
- Impossible de charger les résultats complets
- Réessayer
- Nouvel essai…
- Nouvel essai…
- Hobbies: none / yes (specify)
- Amputee side assessed: right / left
- Amputee side assessed: right / left
- Côté amputé évalué: droit / gauche
- Hand amputated evaluated: right / left
- Main amputée évaluée: droite / gauche
- Impossible de charger les résultats complets
- Réessayer
- Nouvel essai…
- Nouvel essai…
- Amputation level : Upper 1/3 forearm / Middle 1/3 forearm / Lower 1/3 forearm
- Laterality: right-handed / left-handed
- Amputated since (date)
- Etiology : traumatic / congenital / vascular / tumor / other (specify)
- Associated disabilities: none / amputation of the contralateral upper limb (specify level) / other (specify)
- Myoelectric device since
- Current myoelectric hand : Sensor speed / Digital twin / DMC Plus / Vari plus / Other (specify)
- The patient has Greifer hook: yes / noAssociated disabilities: none / amputation of the contralateral upper limb (specify level) / other (specify)
- Handicaps associés: aucun / amputation du membre supérieur controlatéral (préciser le niveau) / autre (préciser)
- Handicaps associates: none / amputation of the contralateral upper extremity (state level) / other (specify)
- Handicaps associés: aucun / amputation du membre supérieur controlatéral (niveau de l'état) / autre (préciser)
- Impossible de charger les résultats complets
- Réessayer
- Nouvel essai…
- Nouvel essai…

Data collected by the investigator during evaluation visits and entered in the observation notebook:

- Hook assessed : Greifer / Axon-Hook
- Start date of the trial period
- Date of the assessment visit
- Number of rehabilitation sessions carried out (minimum 1 session: 1 /2 / other (specify)
- Wrist rotation : passive / motorized
- Hook settings chosen by the patient (degrees: radial tilt (Greifer) / ulnar tilt (Greifer) / flexion (Axon-Hook) / extension (Axon-hook)
- Number of blocks moved in one minute in the Box and Blocks test (assessed side and opposite side)
- Mean shoulder abduction on the Box and Blocks test session on the opposite side (evaluated side and opposite side)
- Percentage of time spent with a shoulder abduction greater than or equal to 60 ° on the Box and Blocks test session (assessed side and opposite side)
- Percentage of time spent with a shoulder abduction greater than or equal to 60 ° on the Box and Blocks test sequence (assessed side and opposite side)
- Pourcentage de temps passé avec une abduction d'épaule supérieure ou égale à 60 ° sur la séquence de test Box and Blocks (côté évalué et côté opposé)
- percentage of time spent with an upper shoulder abduction or equal to 60 ° on the sequence of test Box and Blocks (evaluated side and opposite side)
- pourcentage de temps passé avec un abduction de l'épaule supérieure ou égal à 60 ° sur la séquence de test Box et Blocs (côté évalué et côté opposé)
- Impossible de charger les résultats complets
- Réessayer
- Nouvel essai…
- Nouvel essai…
- ESAT Questionnaire
- Patient préférence: Greifer / Axon-Hook (during the last evaluation only).

Data collected by the investigator at the end of the study:

- Closing date
- Reason for closure: term evaluation / patient lost to follow-up / other (specify)

## Data recording

The data collected during the inclusion visit and the evaluation visits are entered by the investigator in the observation notebook.

The data collected during the inclusion visit and the evaluation visits are entered by the investigator in the observation book.

Les données collectées lors de la visite d'inclusion et des visites d'évaluation sont saisies par l'investigateur dans le livre d'observation.

Data collected during the visit of inclusion and assessment visits are entered by the investigator in the case report.

Les données collectées lors de la visite d'inclusion et des visites d'évaluation sont saisies par l'investigateur dans le rapport de cas.

Impossible de charger les résultats complets

Réessayer

Nouvel essai…

Nouvel essai…

Impossible de charger les résultats complets

Réessayer

Nouvel essai…

Nouvel essai…

For the ESAT questionnaire, the investigator enters his name, the patient's initials and whether the assessment concerns the Axon-Hook or the Greifer on the questionnaire. He then gives the patient the questionnaire asking him to answer all the questions. The investigator then attaches the questionnaire to the patient observation notebook.For the ESAT questionnaire, the investigator enters his name, the patient's initials and whether the assessment concerns the Axon-Hook forceps or the Greifer forceps on the questionnaire.

1. Pour le questionnaire ESAT, l'investigateur inscrit son nom, les initiales du patient et si l'évaluation concerne la pince Axon-Hook ou la pince Greifer sur le questionnaire.

For ESAT questionnaire investigator postpones the questionnaire on his behalf, the patient's initials and says if the evaluation concerns the clip Axon Hook or Greifer gripper.

Pour le questionnaire ESAT, l'investigateur reporte le questionnaire en son nom, les initiales du patient et indique si l'évaluation concerne le clip Axon Hook ou la pince Greifer.

Impossible de charger les résultats complets

Réessayer

Nouvel essai…

Nouvel essai…

He then gives the patient the questionnaire asking him to answer all the questions.

Il remet ensuite au patient le questionnaire lui demandant de répondre à toutes les questions.

He then gives the patient the questionnaire asking him to answer all questions.

Il remet ensuite au patient le questionnaire lui demandant de répondre à toutes les questions.

Impossible de charger les résultats complets

Réessayer

Nouvel essai…

Nouvel essai…

The investigator then attaches the questionnaire to the patient observation booklet.

L'enquêteur joint ensuite le questionnaire au livret d'observation du patient.

The investigator then attached the questionnaire to the patient's observation notebook.

L'enquêteur a ensuite joint le questionnaire au cahier d'observation du patient.

Impossible de charger les résultats complets

Réessayer

Nouvel essai…

Nouvel essai…

## Data archiving

At the end of the study, a copy of the data collected by the investigating physician is sent to the sponsor for analysis and then archiving for 15 years. The investigating doctor keeps the original of all observation notebooks.

At the end of the study, a copy of the data collected by the investigating physician is sent to the sponsor for analysis and then archiving for 15 years.

A l'issue de l'étude, une copie des données collectées par le médecin investigateur est envoyée au promoteur pour analyse puis archivage pendant 15 ans.

At the end of the study, a copy of the data collected by the investigating doctor are sent to the developer for analysis and archiving for 15 years.

A l'issue de l'étude, une copie des données collectées par le médecin investigateur est envoyée au développeur pour analyse et archivage pendant 15 ans.

Impossible de charger les résultats complets

Réessayer

Nouvel essai…

Nouvel essai…

The investigating doctor keeps the original of all observation notebooks.

Le médecin enquêteur conserve l'original de tous les cahiers d'observation.

The investigating physician retain the original of all notebooks observation.

Le médecin investigateur conserve l'original de tous les cahiers d'observation.

Impossible de charger les résultats complets

Réessayer

Nouvel essai…

Nouvel essai…

# Ethical considerations

## Committee for the Protection of Persons

In accordance with the regulations in force in France, the protocol will be submitted for an opinion to the Committee for the Protection of Persons (CPP) and the clinical trial will be the subject of an authorization request from the “Agence Nationale de Sécurité du Médicament et des produits de santé” (ANSM). Any substantial modification of the protocol will also be the subject of a request for a binding opinion from the Committee for the Protection of Persons (CPP) and / or an authorization request from the ANSM. No inclusion will take place before obtaining the ANSM's authorization and the favorable opinion of the CPP, as well as the study setting-up visit by the promoter.

## Patient information and consent

Patients will be informed in a complete and fair manner, in understandable terms, of the objectives and constraints of the study, of the possible risks incurred, of the necessary surveillance and safety measures, of their rights to refuse to participate in the study or the possibility to withdraw at any time. All of this information is contained in an information note and a consent form given to the patient.

All of this information is contained in an information note and a consent form given to the patient.

Toutes ces informations sont contenues dans une note d'information et un formulaire de consentement remis au patient.

All information included in an information note and a form of consent given to the patient.

Toutes les informations sont incluses dans une note d'information et un formulaire de consentement donné au patient.

Impossible de charger les résultats complets

Réessayer

Nouvel essai…

Nouvel essai…

The patient's free, informed and written consent will be obtained by the investigator before inclusion in the study. A copy of the information and consent form signed by both parties will be given to the patient, the investigator will keep the original.

A copy of the information and consent form signed by both parties will be given to the patient, the investigator will keep the original.

Une copie du formulaire d'information et de consentement signé par les deux parties sera remise au patient, l'investigateur gardera l'original.

A copy of the information sheet and consent signed by both parties will be given to the patient, the investigator shall retain the original.

Une copie de la fiche d'information et du consentement signé par les deux parties sera remise au patient, l'investigateur conservera l'original.

Impossible de charger les résultats complets

Réessayer

Nouvel essai…

Nouvel essai…

A copy will be placed at the end of the study in a sealed tamper-evident envelope containing all the consent forms, which will be archived by the promoter.

Patients will be informed in a complete and fair manner, in understandable terms, of the objectives and constraints of the study, of the possible risks incurred, of the necessary surveillance and safety measures, of their rights to refuse to participate in the study.

Les patients seront informés de manière complète et équitable, en termes compréhensibles, des objectifs et des contraintes de l'étude, des risques éventuels encourus, des mesures de surveillance et de sécurité nécessaires, de leurs droits de refuser de participer à l'étude.

Patients should be informed fully and faithfully, in understandable terms, objectives and constraints of the study, potential risks, the necessary surveillance and security measures of their rights to refuse to participate in the study

Les patients doivent être informés pleinement et fidèlement, en termes compréhensibles, des objectifs et des contraintes de l'étude, des risques potentiels, des mesures de surveillance et de sécurité nécessaires de leurs droits de refuser de participer à l'étude.

Impossible de charger les résultats complets

Réessayer

Nouvel essai…

Nouvel essai…

or the possibility to withdraw at any time.

ou la possibilité de se retirer à tout moment.

or the possibility of withdrawal at any time.

ou la possibilité de rétractation à tout moment.

Impossible de charger les résultats complets

Réessayer

Nouvel essai…

Nouvel essai…

Impossible de charger les résultats complets

Réessayer

Nouvel essai…

Nouvel essai…

The patient's free, informed and written consent will be obtained by the investigator before inclusion in the study.

Le consentement libre, éclairé et écrit du patient sera obtenu par l'investigateur avant d'être inclus dans l'étude.

The free and informed consent in writing of the patient will be collected by the investigator prior to enrollment in the study.

Le consentement libre et éclairé par écrit du patient sera recueilli par l'investigateur avant son inscription à l'étude.

Impossible de charger les résultats complets

Réessayer

Nouvel essai…

Nouvel essai…

A copy will be placed at the end of the study in a sealed tamper-evident envelope containing all the consent forms, which will be archived by the promoter.

Une copie sera placée à la fin de l'étude dans une enveloppe scellée inviolable contenant tous les formulaires de consentement, qui sera archivée par le promoteur.

A copy will be placed at the end of study in a sealed envelope inviolable grouping all consent forms, it will be archived by the promoter.

Une copie sera placée en fin d'étude dans une enveloppe scellée inviolable regroupant tous les formulaires de consentement, elle sera archivée par le promoteur.

Impossible de charger les résultats complets

Réessayer

Nouvel essai…

Nouvel essai…

## Financial compensation for subjects

The participation of subjects in the study will not give rise to any compensation. On the other hand, the travel costs incurred by the subject's participation in the study will be covered by the promoter. It is then necessary to use the “travel expenses reimbursement form” which is available from the investigating doctor.

The participation of subjects in the study will not give rise to any compensation.

La participation des sujets à l'étude ne donnera lieu à aucune compensation.

The participation of the subjects in the study will not result in any compensation.

La participation des sujets à l'étude ne donnera lieu à aucune compensation.

Impossible de charger les résultats complets

Réessayer

Nouvel essai…

Nouvel essai…

On the other hand, the travel costs incurred by the subject's participation in the study will be covered by the promoter.

En revanche, les frais de déplacement occasionnés par la participation du sujet à l'étude seront pris en charge par le promoteur.

However, travel expenses incurred by the subject's participation in the study will be covered by the sponsor.

Cependant, les frais de déplacement occasionnés par la participation du sujet à l'étude seront pris en charge par le promoteur.

Impossible de charger les résultats complets

Réessayer

Nouvel essai…

Nouvel essai…

# Financing and assurance

## Financing

The clinical trial is funded by the promoter. A tripartite agreement between Otto Bock France, the investigator and the health establishment defines the conditions for covering the services of the investigation. The Axon-Hook and Greifer hooks required to perform this study will be made available by the promoter for the duration of the trial.

The clinical trial is funded by the sponsor.

L'essai clinique est financé par le promoteur.

The clinical trial is funded by the promoter.

L'essai clinique est financé par le promoteur.

Impossible de charger les résultats complets

Réessayer

Nouvel essai…

Nouvel essai…

A tripartite agreement between Otto Bock France, the investigator and the health establishment defines the conditions for covering the services of the investigation.

Un accord tripartite entre Otto Bock France, l'enquêteur et l'établissement de santé définit les conditions de couverture des services de l'enquête.

A tripartite agreement between Otto Bock France, the investigator and the health institution defines the conditions for support for the investigation of benefits.

Un accord tripartite entre Otto Bock France, l'investigateur et l'établissement de santé définit les conditions de prise en charge de l'enquête sur les prestations.

Impossible de charger les résultats complets

Réessayer

Nouvel essai…

Nouvel essai…

Impossible de charger les résultats complets

Réessayer

Nouvel essai…

Nouvel essai…

## Assurance

For the duration of the study, Otto Bock will take out insurance guaranteeing its own civil liability as well as that of any doctor involved in carrying out the study.

For the duration of the study, Otto Bock will take out insurance guaranteeing its own civil liability as well as that of any doctor involved in carrying out the study.

Pendant toute la durée de l'étude, Otto Bock souscrira une assurance garantissant sa propre responsabilité civile ainsi que celle de tout médecin impliqué dans la réalisation de l'étude.

The Otto Bock company will subscribe for the duration of the study has insurance covering his own liability and that of any doctor involved in the realization of the study.

La société Otto Bock souscrira pour la durée de l'étude a une assurance couvrant sa propre responsabilité et celle de tout médecin impliqué dans la réalisation de l'étude.

Impossible de charger les résultats complets

Réessayer

Nouvel essai…

Nouvel essai…

It will also ensure full compensation for the harmful consequences of research for the person who lends itself to it and his beneficiaries, unless it can be proved that the damage is not attributable to his fault or that of any intervening party, without that may be opposed the act of a third party or the voluntary withdrawal of the person who had initially consented to participate in the research.

It will also ensure full compensation for the harmful consequences of research for the person who lends itself to it and his beneficiaries, unless it can be proved that the damage is not attributable to his fault or that of any intervening party, without

Il assurera également l'indemnisation intégrale des conséquences néfastes de la recherche pour la personne qui s'y prête et ses ayants droit, sauf s'il peut être prouvé que le dommage n'est pas imputable à sa faute ou à celle d'un intervenant, sans

It will also ensure full compensation for the harmful consequences to search for the person undergoing it and assigns, unless evidence against him that the damage is not attributable to its fault or that of any speaker, without

Il assurera également l'indemnisation intégrale des conséquences préjudiciables à la recherche de la personne qui la subit et cède, sauf preuve contre lui que le dommage n'est pas imputable à sa faute ou à celle de tout locuteur, sans

Impossible de charger les résultats complets

Réessayer

Nouvel essai…

Nouvel essai…

that may be opposed the act of a third party or the voluntary withdrawal of the person who had initially consented to participate in the research.

qui peut s'opposer à l'acte d'un tiers ou au retrait volontaire de la personne qui avait initialement consenti à participer à la recherche.

that can be opposite the act of a third party or the voluntary withdrawal of the person who had originally agreed to lend to the research.

qui peut être contraire à l'acte d'un tiers ou au retrait volontaire de la personne qui avait initialement accepté de prêter à la recherche.

Impossible de charger les résultats complets

Réessayer

Nouvel essai…

Nouvel essai…

## Amendments to the clinical investigation plan

The clinical investigation plan may be amended if necessary.

The clinical investigation plan may be amended if necessary.

Le plan d'investigation clinique peut être modifié si nécessaire.

The clinical investigation plan may be subject to amendment if necessary.

Le plan d'investigation clinique peut être sujet à modification si nécessaire.

Impossible de charger les résultats complets

Réessayer

Nouvel essai…

Nouvel essai…

Impossible de charger les résultats complets

Réessayer

Nouvel essai…

Nouvel essai…

The sponsor must inform the investigating physician that the protocol will be amended.

The sponsor must inform the investigating physician that the protocol will be amended.

Le promoteur doit informer le médecin investigateur que le protocole sera modifié.

The proponent must inform the investigating doctor that the protocol will be amended.

Le promoteur doit informer le médecin investigateur que le protocole sera modifié.

Impossible de charger les résultats complets

Réessayer

Nouvel essai…

Nouvel essai…

If deemed necessary, the sponsor may suspend the clinical investigation while the protocol has been amended. This amendment can only be effective after approval by the CPP and the ANSM.

If deemed necessary, the sponsor may suspend the clinical investigation while the protocol has been amended.

S'il le juge nécessaire, le promoteur peut suspendre l'investigation clinique pendant que le protocole a été modifié.

If deemed necessary, the developer may suspend the clinical investigation the time the protocol was amended.

S'il le juge nécessaire, le développeur peut suspendre l'investigation clinique au moment où le protocole a été modifié.

Impossible de charger les résultats complets

Réessayer

Nouvel essai…

Nouvel essai…

This amendment can only be effective after approval by the CPP and the ANSM.

Cette modification ne peut être effective qu'après approbation par le CPP et l'ANSM.

This amendment shall be effective only after approval of the CPP and ANSM.

Cette modification ne sera effective qu'après approbation du CPP et de l'ANSM.

Impossible de charger les résultats complets

Réessayer

Nouvel essai…

Nouvel essai…

# Devices count

The promoter will ensure the availability of the devices under investigation, their identification and their allocation to the investigating center. At the end of the clinical investigation, he will ensure that all devices under investigation have been returned to him.

The sponsor will ensure the availability of the devices under investigation, their identification and their allocation to the investigating center.

Le promoteur s'assurera de la disponibilité des dispositifs faisant l'objet de l'enquête, de leur identification et de leur attribution au centre d'investigation.

The promoter will ensure the provision of devices under investigation, their identification and allocation to the study center.

Le promoteur assurera la mise à disposition des dispositifs sous investigation, leur identification et leur attribution au centre d'étude.

Impossible de charger les résultats complets

Réessayer

Nouvel essai…

Nouvel essai…

At the end of the clinical investigation, he will ensure that all devices under investigation have been returned to him.

À la fin de l'investigation clinique, il s'assurera que tous les dispositifs sous investigation lui ont été retournés.

At the end of the clinical investigation, it will ensure that all devices under investigation were returned.

À la fin de l'investigation clinique, il s'assurera que tous les dispositifs sous investigation ont été retournés.

Impossible de charger les résultats complets

Réessayer

Nouvel essai…

Nouvel essai…

# Premature termination or suspension of the clinical investigation

The clinical investigation may be suspended or terminated prematurely for the entire investigation at the express request of the promoter.

The clinical investigation may be suspended or terminated prematurely for the entire investigation at the express request of the sponsor.

L'investigation clinique peut être suspendue ou interrompue prématurément pour toute l'investigation à la demande expresse du promoteur.

The clinical investigation may be suspended or stopped prematurely for the entire investigation on express request of the developer.

L'investigation clinique peut être suspendue ou arrêtée prématurément pour toute l'investigation sur demande expresse du développeur.

Impossible de charger les résultats complets

Réessayer

Nouvel essai…

Nouvel essai…

Impossible de charger les résultats complets

Réessayer

Nouvel essai…

Nouvel essai…

The criteria justifying this decision are as follows:

The criteria justifying this decision are as follows:

Les critères justifiant cette décision sont les suivants:

The criteria for this decision are as follows:

Les critères de cette décision sont les suivants:

Impossible de charger les résultats complets

Réessayer

Nouvel essai…

Nouvel essai…

- The promoter noted major deviations from the protocol on the part of the investigator
- - The sponsor noted major deviations from the protocol on the part of the investigator
- - Le promoteur a noté des écarts importants par rapport au protocole de la part de l'investigateur
- - The promoter found major protocol deviations from the investigator
- - Le promoteur a trouvé des écarts de protocole majeurs par rapport à l'investigateur
- Impossible de charger les résultats complets
- Réessayer
- Nouvel essai…
- Nouvel essai…
- The promoter has identified a potentially serious risk for the user, for his environment or for the investigation team
- - The promoter has identified a potentially serious risk for the user, for his environment or for the investigation team
- - Le promoteur a identifié un risque potentiellement sérieux pour l'utilisateur, pour son environnement ou pour l'équipe d'enquête
- - The proponent has identified a potentially serious risk to the user, to the environment or to the investigation team
- - Le promoteur a identifié un risque potentiellement grave pour l'utilisateur, l'environnement ou l'équipe d'enquête
- Impossible de charger les résultats complets
- Réessayer
- Nouvel essai…
- Nouvel essai…

In the event of suspension or premature termination of the clinical investigation, the promoter will inform the investigator as soon as possible and notify him of the arrangements to be made.

In the event of suspension or premature termination of the clinical investigation, the sponsor will inform the investigator as soon as possible and notify him of the arrangements to be made.

En cas de suspension ou d'arrêt prématuré de l'investigation clinique, le promoteur informera l'investigateur dans les plus brefs délais et l'informera des dispositions à prendre.

In case of suspension or early termination of the clinical investigation, the promoter will inform the investigator in the shortest possible time and will pass the arrangements.

En cas de suspension ou d'arrêt anticipé de l'investigation clinique, le promoteur informera l'investigateur dans les plus brefs délais et passera les arrangements.

Impossible de charger les résultats complets

Réessayer

Nouvel essai…

Nouvel essai…

He will inform the competent authorities - CPP and ANSM - of the situation of the clinical investigation.

He will inform the competent authorities - CPP and ANSM - of the situation of the clinical investigation.

Il informera les autorités compétentes - CPP et ANSM - de la situation de l'investigation clinique.

He will inform the competent authorities -cpp ANSM- and the situation of the clinical investigation.

Il informera les autorités compétentes -cpp ANSM- et la situation de l'investigation clinique.

Impossible de charger les résultats complets

Réessayer

Nouvel essai…

Nouvel essai…

The scientific communications and reports corresponding to this study must be subject to the prior agreement of the promoter.

Les communications et rapports scientifiques correspondant à cette étude doivent être soumis à l'accord préalable du promoteur.

The papers and scientific reports for this study will necessarily be subject to prior agreement of the promoter.

Les articles et rapports scientifiques de cette étude seront nécessairement soumis à l'accord préalable du promoteur.

Impossible de charger les résultats complets

Réessayer

Nouvel essai…

Nouvel essai…

# Provisional study calendar

CPP and ANSM authorizations: July, 31st 2016

Start of inclusions and study: August, 1st 2016

End of inclusions: September, 30th 2016

End of the study: November, 30th 2016

# Bibliography

*[1] The Psychosocial and Biomechanical Assessment of Amputees Fitted with Commercial Multi-grip Prosthetic Hands. A.G. Cutti, I. Parel, M. Luchetti, E. Gruppioni, N.C. Rossi c and G. Verni. Grasping the Future: Advances in Powered Upper Limb Prosthetics, 2012, 59-77.*

*[2]* [*Major MJ*](http://www.ncbi.nlm.nih.gov/pubmed/?term=Major%20MJ%5BAuthor%5D&cauthor=true&cauthor_uid=25192744)*1,* [*Stine RL*](http://www.ncbi.nlm.nih.gov/pubmed/?term=Stine%20RL%5BAuthor%5D&cauthor=true&cauthor_uid=25192744)*,* [*Heckathorne CW*](http://www.ncbi.nlm.nih.gov/pubmed/?term=Heckathorne%20CW%5BAuthor%5D&cauthor=true&cauthor_uid=25192744)*,* [*Fatone S*](http://www.ncbi.nlm.nih.gov/pubmed/?term=Fatone%20S%5BAuthor%5D&cauthor=true&cauthor_uid=25192744)*,* [*Gard SA*](http://www.ncbi.nlm.nih.gov/pubmed/?term=Gard%20SA%5BAuthor%5D&cauthor=true&cauthor_uid=25192744)*: Comparison of range-of-motion and variability in upper body movements between transradial prosthesis users and able-bodied controls when executing goal-oriented tasks.*[*J Neuroeng Rehabil.*](http://www.ncbi.nlm.nih.gov/pubmed/25192744) *2014 Sep 6;11:132.*

*[3]* *Hebert JS, Lewicke JJ. Case report of modified Box and Blocks test with motion capture to measure prosthetic function. J Rehabil Res Dev. 2012; 49(8):1163–74.*

*[4] Hebert JS, Lewicke JJ. Normative data for modified Box and Blocks test measuring upper-limb function via motion capture. J Rehabil Res Dev. 2014; 51(6):919-932*

*[5] Ostlie K, Franklin RJ, Skjeldal OH, Skrondal A, Magnus P: Musculoskeletal pain and overuse syndromes in adult acquired major upper-limb amputees. Arch Phys Med Rehabil 2011, 92:1967–1973 e1961.*

*[6] Marisol A. Hanley, PhD, Dawn M. Ehde, PhD, Mark Jensen, PhD, Joseph Czerniecki, MD,Douglas G. Smith, MD, and Lawrence R. Robinson, MD. Chronic Pain Associated with Upper-Limb Loss. Am J Phys Med Rehabil. 2009 September; 88(9): 742–779. doi:10.1097/PHM.0b013e3181b306ec*

*[7] Kontaxis A, Cutti AG, Johnson GR, Veeger HE (2009). A framework for the definition of standardized protocols for measuring upper-extremity kinematics. Clin Biomech (Bristol, Avon) 24(3):246-253.*

*[8] Wu G, van der Helm FC, Veeger HE, Makhsous M, Van Roy P, Anglin C, Nagels J, Karduna AR, McQuade K, Wang X, Werner FW, Buchholz B (2005) ISB recommendation on definitions of joint coordinate systems of various joints for the reporting of human joint motion–Part II: shoulder, elbow, wrist and hand. J Biomech 38(5):981–992*

*[9] Parel I, Cutti AG, Kraszewski, Verni G, Hillstrom H, Kontaxis A. Intra-protocol repeatability and inter-protocol agreement for the analysis of scapula-humeral coordination. Med Biol Eng Comput (2014) 52:271-282*

*[10] Demers, L., Weiss-Lambrou, R., & Ska, B. (1996). Development of the Quebec User Evaluation of Satisfaction with Assistive Technology (QUEST). Assistive Technology, 8, 3-13.*

*[11] Mathiowetz V, Volland G, Kashman N, Weber K. Adult norms for the Box and Block Test of manual dexterity. Am J Occup Ther. 1985; 39(6):386–91.*

# Annexes

## Patient information letter

**Information letter**

20PT003-FR-01-0516

Version 2 of July 18, 2016

Note presented and given to the patient by the study investigator

"Randomized crossover trial comparing shoulder abduction movements, manual dexterity and satisfaction of upper limb amputees using Axon-Hook and Greifer."

Promoter: Otto Bock France

4 rue de la Réunion CS90011

91978 Courtaboeuf Cedex

Phone: 01 69 18 88 30

Dear,

Your doctor, Dr Amélie Touillet, suggested that you take part in a study, the title of which appears above. The purpose of this document is to inform you, about this research, of the potential benefits and risks for you if you decide to participate. Indeed, the law requires that all persons who are asked to participate in a clinical study be informed about this study and give their informed consent before being included in the study. You can, on request, have a period of reflection before giving your answer.

If you decide to participate, you will be asked to sign the last page of this document. Your doctor will inform you of any changes occurring during the study which may affect your agreement to continue your participation. At any time, you are absolutely free to ask your doctor any questions you may have regarding this study or your rights.

Introduction

Axon-Hook and Greifer are myoelectric hooks developed by the company Otto Bock which can be used as an alternative to the Michelangelo hand for Axon-Hook (Axon-Bus system) and to a Myobock hand for the Greifer (myobock system) . These are devices that combine strength, precision and resistance to environmental constraints in order to facilitate manual activities for professional or leisure.

The characteristics of these hooks are different:

- Axon-Hook has a fixed lateral fingertip and a movable fingertip as well as a flexible wrist that can be swiveled and lockable in flexion / extension. The grips are made according to an angular movement.

- Greifer has two movable fingertips and a swivel and lockable wrist in medio-lateral tilt. The holds are made in a parallel movement.

The shape/design of the Axon-Hook makes it easy to visualize the objects to be grasped and the passive movements of the wrist make it possible to orient the hook and thus limit stress on the shoulder.

Goal of the study

The objective of this study is to show to what extent the Axon-Hook allows to reduce shoulder abduction movements and increase satisfaction while maintaining the same manual dexterity in comparison with the Greifer (myobock system). The results of this study organized by Otto Bock France will be used to request the support of the Axon-Hook in France. The results may also be used to request Axon-Hook support in other countries.

Benefits

The expected benefits of the Axon-Hook are the reduction in the average abduction of the shoulder during the Box and blocks test, the reduction in the time spent with shoulder abduction greater than or equal to 60 ° and the improvement in satisfaction, in comparison with the Greifer. No significant difference in terms of manual dexterity between the two hooks is expected.

Foreseeable constraints and risks

Axon-Hook and Greifer are myoelectrical hooks that you can use as an alternative to the prosthetic hand. Like all myoelectric prostheses, this one must be recharged daily with intense use. At the end of the Axon-Hook and Greifer trial, or in the event of premature discontinuation of the study, due to your decision or that of your doctor, you must return the devices that were given to you. made available as part of this study.

Course of the study

This study will take place over a total of 4 weeks during which you will use the Axon-Hook for 2 weeks and the Greifer for 2 weeks. The use of each of the grippers as an alternative to the hand will depend on your needs and activities. You can use the Greifer instead of your myobock hand, on your prosthesis. However, a new prosthesis will be made so that you can use the Axon-Hook.

As part of your participation in the study, you will need to go to the center, to your doctor, at the start of each trial for a rehabilitation session and at the end of each trial for a laboratory evaluation of movement. If necessary, additional rehabilitation sessions can be offered to you so that you have perfect control of each device. The device test order is set at random by drawing lots from an envelope by the doctor, once you have given your consent to participate in the study.

Data collected during the study

The personal information collected will be: the initials your first and last name, your sex, age, height, weight, your professional activity, your hobbies, the assessed amputee side, the level of amputation, your laterality, the number of years since the amputation, the reasons for the amputation, your other associated conditions or disabilities, since when you have been fitted with a myoelectric prosthesis, the type of myoelectric hand you currently have and whether or not you have a Greifer. In order to assess the contribution of the prosthetic forceps, the doctor will take a test that involves moving as many cubes as possible from one compartment to another in a box in one minute. This test will take place in a movement lab to record your body movements during this test. This recording requires being shirtless or in underwear. For each assessment, the doctor will give you a questionnaire which you will complete by taking care to answer all the questions in order

to assess your level of satisfaction. At the end of each assessment visit, you will give the

questionnaire to the doctor.

Confidentiality

The data collected will be identified by your initials. As part of this study in which the doctor offers you to participate, processing of your personal data will be implemented to enable the results of the study to be analyzed with regard to the objective of the latter which has been given to you. This data will be collected on paper by your doctor. He will keep the original in his files and send a copy to the sponsor of this study for analysis of the results. This data may, under conditions ensuring their confidentiality, be transmitted to the French health authorities and to other entities of the Otto Bock group. You have a right to oppose the transmission of data covered by professional secrecy that may be used in the context of this study and that may be processed. You can also access all of your medical data directly or through a doctor of your choice in accordance with the provisions of Article L 1111-7 of the Public Health Code. These rights are exercised with the doctor who follows you as part of this study and who knows your identity.

Restitution of the overall results of the research

In accordance with Article L. 1122-1 of the Public Health Code, at the end of the study, you can, if you wish, be informed of the overall results of this study through your doctor.

Social Security

It is mandatory that you are affiliated with a social security scheme to participate in this study.

Participation in another research

Simultaneous participation in other research is not permitted during the planned study period.

Voluntary participation

Your participation in this study is voluntary. You are free to withdraw from this study at any time. The decision to opt out or not to participate will in no way affect the quality of treatment you will receive.

Your participation in the study will not give rise to any compensation. However, the travel costs incurred will be covered by Otto Bock France. You will then have to use the “travel expenses reimbursement request form” which is available from Dr Touillet.

Favorable opinion of the CPP and authorization of the ANSM

This study received an authorization from the National Agency for Medicines and Health Products Safety on July 19, 2016, an approval from the Committee for the Protection of Persons (CPP Est III) on August 29, 2016 and is subject to insurance. specific policy underwritten by Otto Bock France with the Biomedicinsure Insurance Company, Policy N ° 0100534514058-160048-10005.

Contact

For any information concerning the progress of the study, please contact Dr Amélie Touillet. For any information concerning your device, please contact your orthopedic technician.

## Patient consent form

**Consent form of the person participating in the study**

20PT003-FR-01-0516

Version 1 of May 11, 2016

"Randomized crossover trial comparing shoulder abduction movements, manual dexterity, and satisfaction in upper limb amputees using Axon-Hook and Greifer."

I the undersigned………………………………………………………………….declares to have been informed by Doctor Amélie TOUILLET of the Louis Pierquin center - Regional Rehabilitation Institute of Nancy- of the objectives and constraints of the study: "Randomized crossover trial comparing the abduction movements of the shoulder, manual dexterity and satisfaction of upper limb amputees using Axon-Hook and Greifer."

Dr Touillet gave me an information letter about the study that I learned about.

I agree to participate in this study and authorize the collection of my results. I have been informed that this data will be treated confidentially by paper without revealing my identity. I have noted that the right of access provided for by the law of January 6, 1978 relating to data processing, files and freedoms (article 39) can be exercised at any time with the doctor who follows me within the framework of research and who knows my identity. I can exercise my right of rectification and opposition with the same doctor who will contact the study sponsor.

My possible refusal to all or one of the exams will not change the way I will be treated. In addition, my acceptance to participate in no way releases the responsibility of the doctors who took care of me and those responsible for the study.

I know that my participation in this study is voluntary and that I am free to withdraw from this study at any time, just as the doctor who will follow me can remove me from the study if he deems it necessary. My decision to withdraw from the study will in no way affect the quality of the treatment I will receive.

Any new information concerning me that may modify my consent will be communicated to me.

I know that I have had the opportunity to request a cooling-off period before giving my consent.

I certify that all my questions have been answered and that I may ask more at any time.

I accept that the data recorded during this study may be subject to computer processing by the promoter or on his behalf.

This document will be drawn up in two originals, one of which will be kept for 15 years by the investigator.

Name of the person participating in the study (in capital letters): ……………………..........................................................................

Date: ............................................................ (MM/DD/YYYY)

Signature of the person participating in the study:

Name of the Doctor (in capital letters):

..............................................................................................

Date: ............................................................. (MM/DD/YYYY)

Doctor’s signature:

## Observation notebook

**Observation notebook**

"Randomized crossover trial comparing shoulder compensatory movements, manual dexterity and satisfaction in upper limb amputees using Axon-Hook and Greifer."

Patient initials

………. - ……….

**Inclusion**

Date of inclusion visit: .......... / ………. / ……….

VERIFICATION OF INCLUSION AND EXCLUSION CRITERIA

**Inclusion criteria**

| - people with upper limb amputees at the transradi-ulnar level | ☐ Yes ☐ No |
| --- | --- |
| - people with acquired or congenital amputation | ☐ Yes ☐ No |
| - people who regularly use a myoelectric prosthesis and who control it well | ☐ Yes ☐ No |
| - people whose residual limb is stabilized and who are at least six months away from the amputation | ☐ Yes ☐ No |
| - people whose professional activity or life project justifies or could justify the   use of a myoelectric hook. | ☐ Yes ☐ No |
| - people who have given their free and informed consent in writing | ☐ Yes ☐ No |

**Exclusion criteria**

| - people under 18 | ☐ Yes ☐ No |
| --- | --- |
| - pregnant women | ☐ Yes ☐ No |
| - people in emergency situations | ☐ Yes ☐ No |
| - people unable to give their consent personally | ☐ Yes ☐ No |
| - people with a mental or linguistic inability to understand the instructions for taking the research tests - people not available to comply with the entire study protocol | ☐ Yes ☐ No  ☐ Yes ☐ No |
|  |  |

PATIENT PROFILE DESCRIPTION

**Patient characteristics**

| Sex : | ☐ Man ☐ Woman |
| --- | --- |
| Age : | ………………………………. years |
| Height: | ………………………………. cm |
| Weight : | ………………………………. kg |
| Professional activity : | ☐ student ☐ retired ☐ unemployed  ☐ in professional activity (specify): …………………………………………………… |
| Hobbies: | ☐ no  ☐ yes (specify)……………………………………………………………………………………  …………………………………………………………………………………………………………… |
| **Pathology** |  |
| Amputee side assessed : | ☐ right ☐ left |
| Amputation level : | ☐ 1/3 sup. avant-bras ☐1/3 moyen avant-bras ☐1/3 inf. avant-bras |
| Laterality: | ☐ right handed ☐ left handed |
| Amputated since (date) : | .......... / ………. / ………. |
| Etiology : | ☐ traumatic ☐ congenital ☐vascular ☐ tumoral  ☐ other: …………………………………………………………………………………………… |
| Associated disabilities : | ☐ no |
|  | ☐ amputation of the controlateral upper limb  (specify level) :……………………………………………………………………………… |
|  | ☐ other: …………………………………………………………………………………………… |
| **Usual prosthetic devices** |  |

Fitted with myoelectric since (date): .......... / ………. / ……….

| Current myoelectric hand: | ☐ Sensor Speed ☐ Digital twin ☐ DMC Plus ☐ Vari plus  ☐ Other: ........................................................................................... |
| --- | --- |
| The patient has a Greifer : | ☐ Yes ☐ No |

PATIENT INFORMED INFORAMTION AND CONSENT

After informing my patient of the study, both orally and in writing, he informed me of his decision:

☐ he wishes to participate in the study and has given me the signed consent form

☐ he does not wish to participate in the study for the following reason (s):………………………………………...

………………………………………………………………………………………………………………………………………………………….

RANDOMISATION

The envelope drawn told me the following order of evaluation of the devices:

☐ Axon-hook then Greifer

☐ Greifer then Axon-Hook

**First evaluation**

Hook assessed: ☐ Greifer ☐ Axon-Hook

Start date of the trial period: .......... / ………. / ………

Date of the first assessment visit (after min. 14 days and max. 28 days): .......... / ………. / ……….

Number of rehabilitation sessions performed (minimum 1 session): ☐ 1 ☐ 2 ☐ other: …….

Wrist rotation: ☐ passive ☐ motorized

Hook adjustment chosen by the patient: …………… degrees

Greifer => ☐ radial inclination ☐ cubital inclination

Axon-Hook => ☐ flexion ☐ extension

MANUAL DEXTERITY & BIOMECHANICAL ANALYSIS

|  | **Assessed**  **Side** | **Controlateral Side** |
| --- | --- | --- |
| Number of cubes moved in 1 minute |  |  |
| Mean shoulder abduction | ± | ± |
| Percentage of time with shoulder abduction ≥ 60° | % | % |

SATISFACTION

I entered the patient's initials and mentioned the device on the ESAT questionnaire: v ☐ yes ☐ no

I have verified that the patient has answered all the questions of the ESAT questionnaire: ☐ yes ☐ no

**Second evaluation**

Hook assessed: ☐ Greifer ☐ Axon-Hook

Start date of the trial period: .......... / ………. / ………

Date of the first assessment visit (after min. 14 days and max. 28 days): .......... / ………. /……….

Wrist rotation: similar to the first evaluation

Hook adjustment chosen by the patient: …………… degrees

Greifer => ☐ radial inclination ☐ cubital inclination

Axon-Hook => ☐ flexion ☐ extension

DEXTERITE MANUELLE & ANALYSE BIOMECANIQUE

|  | **Side assessed** |
| --- | --- |
| Number of cubes moved in 1 minute |  |
| Mean shoulder abduction | ± |
| Percentage of time with shoulder abduction ≥ 60° | % |

SATISFACTION

I entered the patient's initials and mentioned the device on the ESAT questionnaire: v ☐ yes ☐ no

I have verified that the patient has answered all the questions of the ESAT questionnaire: ☐ yes ☐ no

PREFERENCE

The patient prefers the following device: ☐ Greifer ☐ Axon-Hook

**Adverse events**

ADVERSE EVENT 1

| Date of onset of adverse event: | .......... / ………. / ………. |
| --- | --- |
| Device evaluated during the onset of the adverse event | ☐ Greifer ☐ Axon-Hook |
| Description of this adverse event : | …………………………………………………………………………………………………… |
|  | …………………………………………………………………………………………………… |
|  | …………………………………………………………………………………………………… |
| Event related to the myoelectric hook: | ☐ yes ☐ no ☐ possible |
| Gravity : | ☐ death ☐ life threatening risk |
|  | ☐ significant or lasting disability or handicap |
|  | ☐ hospitalization ☐ prolongation of hospitalization |
|  | ☐ potentially serious event ☐ non-serious event |
| Adverse event intensity: | ☐ mid grade 1 ☐ moderate grade 2 ☐ severe grade 3 |
| Adverse event evolution: | …………………………………………………………………………………………………… |
|  | …………………………………………………………………………………………………… |
|  | …………………………………………………………………………………………………… |
| Action taken : | ☐ no monitoring ☐ adjustment ☐ permanent shutdown |
| Relevant additional information: | …………………………………………………………………………………………………… |
|  | …………………………………………………………………………………………………… |
| Adverse event still ongoing: | ☐ yes ☐ no |
| Date of resolution of the adverse event: | .......... / ………. / ………. |

ADVERSE EVENT 2

| Date of onset of adverse event: | .......... / ………. / ………. |
| --- | --- |
| Device evaluated during the onset of the adverse event | ☐ Greifer ☐ Axon-Hook |
| Description of this adverse event : | …………………………………………………………………………………………………… |
|  | …………………………………………………………………………………………………… |
|  | …………………………………………………………………………………………………… |
| Event related to the myoelectric hook: | ☐ yes ☐ no ☐ possible |
| Gravity : | ☐ death ☐ life threatening risk |
|  | ☐ significant or lasting disability or handicap |
|  | ☐ hospitalization ☐ prolongation of hospitalization |
|  | ☐ potentially serious event ☐ no serious event |
| Adverse event intensity: | ☐ mid grade 1 ☐ moderate grade 2 ☐ severe grade 3 |
| Adverse event evolution: | …………………………………………………………………………………………………… |
|  | …………………………………………………………………………………………………… |
|  | …………………………………………………………………………………………………… |
| Action taken : | ☐ no monitoring ☐ adjustment ☐ permanent shutdown |
| Relevant additional information: | …………………………………………………………………………………………………… |
|  | …………………………………………………………………………………………………… |
| Adverse event still ongoing: | ☐ yes ☐ no |
| Date of resolution of the adverse event: | .......... / ………. / ………. |

**Study closure**

Study closure date: .......... / ………. / ……….

Reason of closure: ☐ term of evaluation ☐ Patient lost to follow-up ☐ other (specify): ………………………………

…………………………………………………………………………………………………………………………………………………………………………….

First name, Last name:………………………………………………

Doctor’s signature: Center stamp:

## QUEST Questionnaire

***Frame reserved for the investigating doctor***

Stamp Patient initials: ………. - ……….

Device under investigation: 🞏 Greifer 🞏 Axon-Hook

| **1** | **2** | **3** | **4** | **5** |
| --- | --- | --- | --- | --- |
| Not satisfied at all | Not very satisfied | More or less satisfied | Quite satisfied | Very satisfied |

*Please circle or mark the* ***one number*** *that best describes your degree of satisfaction with each of the 12 items.*

***Do not leave any question unanswered.***

*For any item that you were not « very satisfied », please comment in the section comments.*

| **ASSISTIVE DEVICE**  *How satisfied are you with,* | |
| --- | --- |
| 1. The **dimensions** (size, height, length, width) of your assistive device?  *Comments :* | 1 2 3 4 5 |
| 2. The **weight** of you assistive device?  *Comments :* | 1 2 3 4 5 |
| 3. The **ease in adjusting** (fixing, fastening) the parts of your assistive device?  *Comments :* | 1 2 3 4 5 |
| 4. How **safe and secure** your assistive device is?  *Comments :* | 1 2 3 4 5 |
| 5. The **durability** (endurance, resistance to wear) of your assistive device?  *Comments :* | 1 2 3 4 5 |
| 6. How **easy** it is to use your assistive device?  *Comments :* | 1 2 3 4 5 |
| 7. How **comfortable** your assistive device is?  *Comments :* | 1 2 3 4 5 |
| 8. How **effective** your assistive device is (the degree to which your device meets your needs)?  *Comments :* | 1 2 3 4 5 |

QUEST - Page 1/2

| **1** | **2** | **3** | **4** | **5** |
| --- | --- | --- | --- | --- |
| Not satisfied at all | Not very satisfied | More or less satisfied | Quite satisfied | Very satisfied |

*Please circle or mark the* ***one number*** *that best describes your degree of satisfaction with each of the 12 items.*

***Do not leave any question unanswered.***

*For any item that you were not « very satisfied », please comment in the section comments.*

| **SERVICES**  *How satisfied are you with,* | |
| --- | --- |
| 9. The **services delivery** program (procedures, delay of time) in which you obtained your assistive device ?  *Comments :* | 1 2 3 4 5 |
| 10. The **repairs and servicing** provided for your assistive device?  *Comments :* | 1 2 3 4 5 |
| 11. The quality of the **professional services** (information, attention) you receives for using your assistive device?  *Comments :* | 1 2 3 4 5 |
| 12. The **follow-up services** (continuing support services) received for your assistive device?  *Comments :* | 1 2 3 4 5 |

- Below is the list of the same 12 satisfaction items. PLEASE **SELECT THE THREE ITEMS** that you consider to be the most important to you. Please put an X in the **3 boxes** of your choice.

□ 1. Dimensions □ 7. Comfort

□ 2. Weight □ 8. Effectiveness

□ 3. Adjustments □ 9. Service delivery

□ 4. Safety □ 10. Repairs / servicing

□ 5. Solidity □ 11. Professional service

□ 6. Easy to use □ 12. Follow-up services

*© L. Demers, R. Weiss-Lambrou & B. Ska, 2000*

## Declaration of conformity with the CNIL


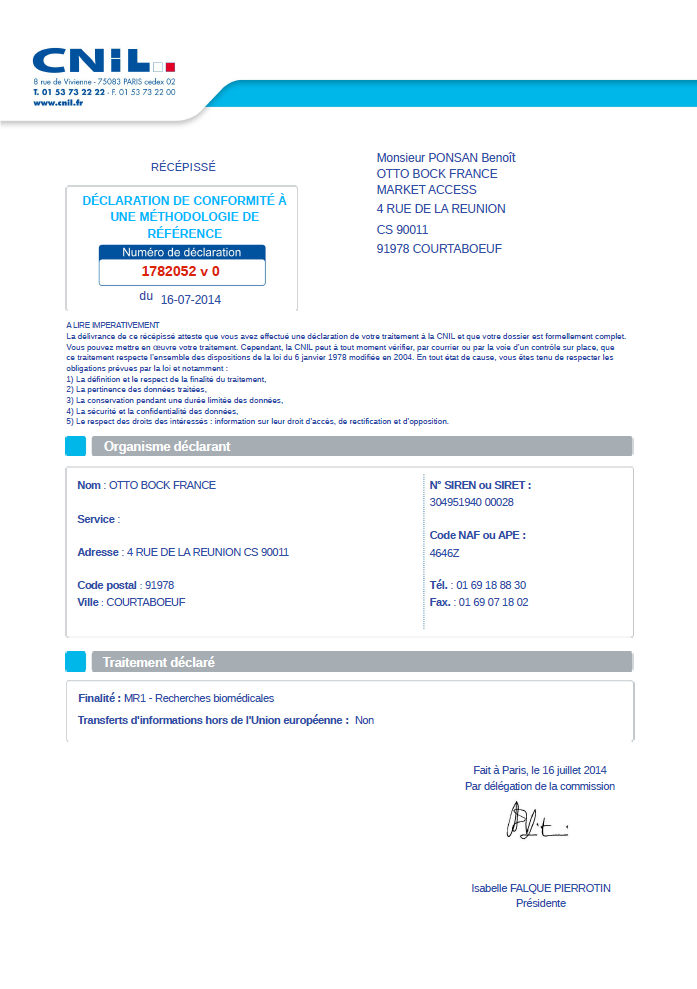

Supplement: S1 Protocol — (DOCX) [file pone.0272855.s002.docx]
