# Supplementary material for: Comparison of compensatory shoulder movements, functionality and satisfaction in transradial amputees fitted with two prosthetic myoelectric hooks
Source: PLoS One. 2023 Feb 2;18(2):e0272855. doi: 10.1371/journal.pone.0272855 (PMC9894487; doi:10.1371/journal.pone.0272855)

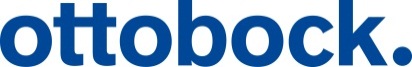


**PROTOCOLE**

**D’ESSAI CLINIQUE**

Essai randomisé croisé comparant les mouvements d’abduction de l’épaule, la dextérité manuelle et la satisfaction de personnes amputées du membre supérieur utilisant la pince Axon-Hook et la pince Greifer.

DOCUMENT CONFIDENTIEL

| PROMOTEUR : | OTTO BOCK FRANCE  4 rue de la Réunion CS 90011 - 91978 COURTABOEUF Cedex  Tel : 01 68 18 88 30 / Fax : 01 69 07 18 02 |
| --- | --- |
| **PROTOCOLE D’ESSAI CLINIQUE**  **Titre abrégé**  Evaluation de la compensation du handicap avec la pince Axon-Hook | |
| CODE ESSAI | ESSAI-2016-AXON-HOOK |
| N° ID RCB (ANSM) | 2016-A00897-44 |
| DISPOSITIF MEDICAL | AXON HOOK Otto Bock  Pince myoélectrique pour prothèse externe du membre supérieur – Dispositif médical de classe 1  Références produit : 8E600 |
| TITRE COMPLET | Essai randomisé croisé comparant les mouvements d’abduction de l’épaule, la dextérité manuelle et la satisfaction de personnes amputées du membre supérieur utilisant la pince Axon-Hook et la pince Greifer. |
| PHASE CLINIQUE | Le dispositif Axon-Hook dispose du marquage CE et sera utilisé dans le cadre défini par le marquage CE |
| INDICATION(S) (CIBLE) | - personnes amputées du membre supérieur au niveau transradio ulnaire - personnes dont l’amputation est acquise ou congénitale - personnes utilisatrices d’une prothèse myoélectrique et en ayant la maîtrise - personnes dont le membre résiduel est stabilisé et qui sont au minimum à six mois de l’amputation - personnes dont l’activité professionnelle ou le projet de vie justifie ou pourrait justifier de l’utilisation d’une pince myoélectrique. - personnes ayant donné par écrit leur consentement libre et éclairé |
| INVESTIGATEUR PRINCIPAL | Dr. Amélie TOUILLET – Médecin MPR (Médecine Physique et Réadaptation)  Institut Régional de Médecine Physique et de Réadaptation - 75 boulevard Lobau 54042 Nancy – France |
|  |  |
| VERSION DU PROTOCOLE | Version 2 |
| DATE DU PROTOCOLE | 18 juillet 2016 |
| CPP | Approuvé le 29 août 2016  Par le Comité de Protection des Personnes EST III |
| ANSM | Approuvé le 19 juillet 2016 |
| **CE DOCUMENT CONFIDENTIEL EST LA PROPRIETE D’OTTO BOCK FRANCE**  **AUCUNE INFORMATION NON PUBLIEE FIGURANT DANS CE DOCUMENT NE PEUT ETRE DIVULGUEE SANS AUTORISATION ECRITE PREALABLE D’OTTO BOCK FRANCE** | |

**Liste des abréviations**

| ANSM | Agence Nationale de Sécurité du Médicament et des produits de santé des Produits de Santé |
| --- | --- |
| B&B | Box and Blocks |
| CNEDiMTS | Commission Nationale d’Evaluation des Dispositifs Médicaux et des Technologies de Santé |
| CNIL | Commission Nationale de l’Informatique et des Libertés en France |
| CPP | Comité de Protection des Personnes en France |
| DM | Dispositif Médical |
| EvI | Evènement Indésirable |
| EvIG | Evènement Indésirable Grave |
| EI | Effet Indésirable |
| EIG | Effet Indésirable Grave |
| ESAT | Evaluation de la Satisfaction envers une Aide Technique (version française du QUEST) |
| HAS | Haute Autorité de Santé en France |
| MPR | Médecine Physique et Réadaptation |

**Sommaire**

[1 Généralités 5](#_Toc451432002)

[1.1 Identification du plan d'investigation clinique 6](#_Toc451432003)

[1.2 Promoteur 6](#_Toc451432004)

[1.3 Investigateur 6](#_Toc451432005)

[1.4 Comité de surveillance 6](#_Toc451432006)

[1.5 Synopsis général de l'investigation clinique 7](#_Toc451432007)

[2 Dispositif médical à l’essai 10](#_Toc451432008)

[2.1 Description générale du dispositif 10](#_Toc451432009)

[2.2 Destination prévue du dispositif 10](#_Toc451432010)

[2.3 Objectif prévu dans l'investigation clinique 10](#_Toc451432011)

[2.4 Population et indications 10](#_Toc451432012)

[2.5 Instructions de mise en place et d’utilisation du dispositif 10](#_Toc451432013)

[2.6 Contre-indications 11](#_Toc451432014)

[2.7 Fabricant et distribution 11](#_Toc451432015)

[2.8 Identification et traçabilité 11](#_Toc451432016)

[3 Justification de la conception de l'investigation clinique 11](#_Toc451432017)

[3.1 Description de la prise en charge existante et de ses limites 11](#_Toc451432018)

[3.2 Résultats des essais disponibles et pertinents au regard de l’étude 11](#_Toc451432019)

[4 Risques et bénéfices du dispositif médical à l’essai et de l'investigation clinique 11](#_Toc451432020)

[4.1 Avantages cliniques attendus 11](#_Toc451432021)

[4.2 Risques 11](#_Toc451432022)

[4.3 Étapes entreprises pour maîtriser ou atténuer les risques. 11](#_Toc451432023)

[4.4 Justification du rapport bénéfice/risque. 11](#_Toc451432024)

[5 Objectifs et hypothèses de l'investigation clinique 12](#_Toc451432025)

[5.1 Objectifs principaux et secondaires 12](#_Toc451432026)

[5.1.1 Objectif principal 12](#_Toc451432027)

[5.1.2 Objectifs secondaires 12](#_Toc451432028)

[5.2 Hypothèses principales et secondaires 12](#_Toc451432029)

[5.2.1 Hypothèse principale 12](#_Toc451432030)

[5.2.2 Hypothèses secondaires 12](#_Toc451432031)

[6 Conception de l'investigation clinique 12](#_Toc451432032)

[6.1 Critères d’évaluation principaux et secondaires 12](#_Toc451432033)

[6.1.1 Critère d’évaluation principal 12](#_Toc451432034)

[6.1.2 Critères d’évaluation secondaires 12](#_Toc451432035)

[6.2 Plan expérimental 13](#_Toc451432036)

[6.3 Dispositif médical à l’essai et comparateur 14](#_Toc451432037)

[6.3.1 Description de l'exposition au dispositif médical d’essai et au comparateur. 14](#_Toc451432038)

[6.3.2 Justification du choix du comparateur. 14](#_Toc451432039)

[6.4 Population étudiée 14](#_Toc451432040)

[6.4.1 Critères d'inclusion 14](#_Toc451432041)

[6.4.2 Critères de non inclusion 14](#_Toc451432042)

[6.5 Déroulement pratique de l’investigation clinique 15](#_Toc451432043)

[6.5.1 Modalités de recrutement 15](#_Toc451432044)

[6.5.2 Modalités d’information et de recueil du consentement 15](#_Toc451432045)

[6.5.3 Méthodes de randomisation 15](#_Toc451432046)

[6.5.4 Actes pratiqués pour l’appareillage et la rééducation 15](#_Toc451432047)

[6.5.5 Équipement nécessaire à l’investigation clinique 15](#_Toc451432048)

[6.5.6 Sortie d’étude ou arrêt prématuré du suivi 16](#_Toc451432049)

[6.5.7 Procédures de remplacement des sujets, le cas échéant 17](#_Toc451432050)

[6.5.8 Chronologie des visites 17](#_Toc451432051)

[6.5.9 Durée de participation pour chaque sujet et durée totale de l'investigation clinique 17](#_Toc451432052)

[6.6 Gestion des évènements indésirables 17](#_Toc451432053)

[6.6.1 Définitions 17](#_Toc451432054)

[6.6.2 Liste des événements indésirables prévisibles et des effets indésirables attendus du dispositif, leur incidence probable, le moyen d'atténuation ou le traitement 18](#_Toc451432055)

[6.6.3 Rôle de l’investigateur 18](#_Toc451432056)

[6.6.4 Rôle du promoteur 19](#_Toc451432057)

[6.7 Plan de surveillance 19](#_Toc451432058)

[7 Statistiques 19](#_Toc451432059)

[7.1 Taille de l'échantillon 19](#_Toc451432060)

[7.2 Traitement des données manquantes, inutilisées ou erronées 1](#_Toc451432061)9

[7.3 Populations d’analyse 20](#_Toc451432062)

[7.4 Analyses statistiques 20](#_Toc451432063)

[8 Contrôle et assurance qualité 20](#_Toc451432064)

[8.1 Promoteur 20](#_Toc451432065)

[8.2 Collecte des données 20](#_Toc451432066)

[9 Gestion des données 20](#_Toc451432067)

[9.1 CNIL 20](#_Toc451432068)

[9.2 Données recueillies 21](#_Toc451432069)

[9.3 Enregistrement des données 22](#_Toc451432070)

[9.4 ARCHIVAGE DES DONNEES 22](#_Toc451432071)

[10 Considérations éthiques 22](#_Toc451432072)

[10.1 Comité de protection des personnes 22](#_Toc451432073)

[10.2 Information et consentement du patient 22](#_Toc451432074)

[10.3 Indemnisation des sujets 22](#_Toc451432075)

[11 Financement et assurances 22](#_Toc451432076)

[11.1 Financement 22](#_Toc451432077)

[11.2 Assurance 23](#_Toc451432078)

[11.3 Amendements du plan d'investigation clinique 23](#_Toc451432079)

[12 Décompte des dispositifs 23](#_Toc451432080)

[13 Arrêt prématuré ou suspension de l'investigation clinique 23](#_Toc451432081)

[14 Confidentialité et publication des résultats 23](#_Toc451432082)

[15 Calendrier prévisionnel de l’étude 23](#_Toc451432083)

[16 Bibliographie 24](#_Toc451432084)

[17 Annexes 26](#_Toc451432085)

[17.1 Lettre d’information au sujet participant à l’étude 26](#_Toc451432086)

[17.2 Formulaire de consentement de la personne 28](#_Toc451432087)

[17.3 Cahier d’observation 29](#_Toc451432088)

[17.4 Questionnaire de satisfaction du patient ESAT 33](#_Toc451432089)

[17.5 Déclaration de conformité à la CNIL 35](#_Toc451432090)

# Généralités

## Identification du plan d'investigation clinique

Titre de l’étude: Essai randomisé croisé comparant les mouvements d’abduction de l’épaule, la dextérité manuelle et la satisfaction de personnes amputées au niveau transradio ulnaire utilisant la pince Axon-Hook et la pince Greifer.

Titre abrégé: Evaluation de la compensation du handicap avec la pince Axon-Hook.

Code de référence: ESSAI-2016-AXON-HOOK

Version et date du plan d’investigation clinique : Version 2 du 18 juillet 2016

## Promoteur

Identité du promoteur

OTTO BOCK France

4 rue de la Réunion, CS 90011, 91978 COURTABOEUF Cedex, Tel : 01 68 18 88 30 / Fax : 01 69 07 18 02

Responsable de l’étude au niveau du promoteur

Dr. Philippe Henry Directeur Médical Otto Bock France

Coordination et suivi de l’étude au niveau du promoteur

Ing. Benoit Ponsan Responsable accès au marché Otto Bock France

Ing. Aurelie Lacroix Orthoprothésiste Responsable assurance qualité Otto Bock France

## Investigateur

Investigateur

Dr. Amélie Touillet Médecin de médecine Physique et de Réadaptation

Institut Régional de Médecine Physique et de Réadaptation

75 boulevard Lobau 54042 Nancy – France

## Comité de surveillance

L’essai ne nécessite pas de comité de surveillance puisqu’il n’est pas réalisé en double-aveugle. Tout évènement indésirable grave et inattendu susceptible d’être dû au dispositif faisant l’objet de l’étude ou tout évènement indésirable grave pouvant être lié au geste de mise en place du dispositif sera communiqué aux autorités compétentes.

## Synopsis général de l'investigation clinique

| Titre | Essai randomisé croisé comparant les mouvements d’abduction de l’épaule, la dextérité manuelle et la satisfaction de personnes amputées du membre supérieur utilisant la pince Axon-Hook et la pince Greifer. |
| --- | --- |
| titre abrégé | Evaluation de la compensation du handicap avec la pince Axon-Hook. |
| Promoteur | Otto Bock France |
| Investigateur | Dr. Amélie TOUILLET – Médecin MPR (Médecine Physique et Réadaptation)  Institut Régional de Médecine Physique et de Réadaptation - 75 boulevard Lobau 54042 Nancy – France |
| Version du protocole | Version 2 du 18 juillet 2016 |
| Justification / contexte | Pour certaines activités professionnelles ou de loisirs, les utilisateurs de prothèses myoélectriques vont trouver une meilleure réponse fonctionnelle avec un dispositif terminal non morphologique. Actuellement, la pince Greifer, inscrite à la LPPR sous nom de marque dans le système Myobock d’Otto Bock est une pince très utilisée. Elle dispose de deux mâchoires parallèles mobiles pour la préhension des objets et d’un poignet orientable et verrouillable en inclinaison médio-latérale. L’absence de mouvements du poignet en flexion / extension associé au volume de la pince qui limite la vision les prises induisent des mouvements de compensation tels que l’augmentation de l’abduction de l’épaule.  Selon les données de l’INRS (1), des travaux comportant des mouvements ou le maintien de l’épaule sans soutien en abduction avec un angle supérieur ou égal à 60° pendant au moins deux heures par jour en cumulé peut être à l’origine de troubles musculosquelettiques de l’épaule. Ainsi, plus un sujet est exposé à des mouvements d’abduction de l’épaule importants et fréquents, plus il risque des développer des affections péri articulaires de l’épaule. Il est important de noter que l’épaule du sujet amputé est soumise à une contrainte supplémentaire par rapport au sujet sain qui est le poids de la prothèse.  En 2015, Otto Bock a mis sur le marché la pince Axon-Hook, se différenciant par rapport au Greifer par la flexion/extension possible du poignet et par des mâchoires plus fines dont une est fixe pour permettre la prise d’objets avec davantage de précision et de facilité.  Cette étude vise à mettre en évidence la diminution des mouvements d’abduction de l’épaule avec Axon-Hook par rapport au Greifer et sera utilisée afin de demander le remboursement de ce dispositif auprès de la Haute Autorité de Santé.   1. <http://www.inrs-mp.fr/mp/cgi-bin/mppage.pl?tabkey=TAB_RG57> |
| Objectif Principal | Comparer les mouvements d’abduction de l’épaule lors de déplacements d’objets avec la pince Axon-Hook et la pince Greifer chez des sujets amputés transradio-ulnaire utilisateurs d’une prothèse myoélectrique. |
| Objectifs Secondaires | Comparer le pourcentage de temps passé avec une abduction d’épaule supérieure à 60° lors du test Box and Blocks, la dextérité manuelle et la satisfaction avec la pince Axon-Hook et la pince Greifer chez des sujets amputés transradio ulnaire utilisateurs d’une prothèse myoélectrique. |
| Critère de Jugement Principal | - Mesure de l’angle moyen d’abduction de l’épaule lors du test Box and Blocks.   Une réduction de l’angle moyen d’abduction lors de la manipulation des objets est une amélioration du service rendu par le dispositif car la personne diminue les mouvements de compensation lors de l’utilisation de la prothèse. |
| Critères de Jugement Secondaires | - Pourcentage de temps passé avec une abduction moyenne de l’épaule supérieure ou égale à 60° lors du test Box and Blocks - Dextérité manuelle : score au test Box and Blocks - Satisfaction : score obtenu à l’auto-questionnaire ESAT (Evaluation de la Satisfaction envers une Aide Technique) - Préférence du patient entre la pince Greifer et la pince AxonHook |
| Plan expérimental | Etude monocentrique, comparative, randomisée, croisée, réalisée en ouvert |
| Critères d’Inclusion des Sujets | - personnes amputées du membre supérieur au niveau transradio ulnaire - personnes dont l’amputation est acquise ou congénitale - personnes utilisatrices régulières d’une prothèse myoélectrique et en ayant la maîtrise - personnes dont le membre résiduel est stabilisé et qui sont au minimum à six mois de l’amputation - personnes dont l’activité professionnelle ou le projet de vie justifie ou pourrait justifier de l’utilisation d’une pince myoélectrique. - personnes ayant donné par écrit leur consentement libre et éclairé |
| Critères de Non-Inclusion des Sujets | - personnes de moins de 18 ans - femmes enceintes - personnes en situation d’urgence - personnes incapables de donner personnellement leur consentement - personnes dans l’incapacité psychique ou linguistique de comprendre les instructions de passation des tests de la recherche - personnes non disponibles pour respecter la totalité du protocole d’étude |
| Dispositifs | - AXON-HOOK : Axon-Hook est une pince myoélectrique du système Axon-Bus pour prothèse externe du membre supérieur. Elle dispose d’une tige latérale fixe et d’une tige médiane mobile ainsi que d’un poignet flexible orientable et verrouillable en flexion / extension. Les prises se font selon un mouvement angulaire.   La pince Axon-Hook est un dispositif terminal non morphologique pour prothèse myoélectrique du membre supérieur. Ses caractéristiques techniques en font un outil qui allie force, précision et résistance aux contraintes environnementales pour la réalisation d’activités manuelles à titre professionnel ou de loisirs. La pince Axon-Hook s’inscrit en alternative de la pince myoélectrique Greifer.   - GREIFER : Greifer est une pince myoélectrique du système myobock pour prothèse externe du membre supérieur. Elle dispose de deux mâchoires mobiles et d’un poignet orientable et verrouillable en inclinaison médio-latérale. Les prises se font selon un mouvement parallèle. Ce dispositif fabriqué et distribué par Otto Bock figure à la LPPR sous nom de marque. Il s’agit du comparateur de l’étude. |
| Déroulement pratique de l’essai | Pendant la période d’inclusion, l’investigateur propose à tous ses patients suivis par le centre et répondant aux critères d’inclusion de participer à l’essai. L’ordre d’évaluation des dispositifs est randomisé. Celle-ci se fait par l’intermédiaire d’enveloppes scellées, préparées par le promoteur et données à l’investigateur avant le début de l’étude. Chaque enveloppe contient chacune un ordre d’évaluation des dispositifs : 4 enveloppes mentionnent l’ordre d’essai « Axon-Hook puis Greifer » et 4 autres enveloppes mentionnent l’ordre d’essai « Greifer puis Axon-Hook ». Lors de la visite d’inclusion et après obtention de l’accord de participation du patient, l’investigateur tire au sort une enveloppe afin de définir l’ordre d’essai des dispositifs.    Au début de chaque période d’essai, le patient bénéficie d’une séance de rééducation minimum afin de s’assurer qu’il maitrise l’utilisation de la pince. Après deux semaines, le patient est évalué avec le dispositif d’essai (tolérance de + 2 semaines). |
| Nombre de patients | Le calcul du nombre de patients à inclure est basé sur la différence attendue sur le critère principal, l’abduction moyenne de l’épaule lors de la passation du test Box and Blocks. Avec l’hypothèse d’une différence d’abduction moyenne de 30 ± 25 degrés il est nécessaire d’inclure 8 patients pour un essai randomisé réalisé en cross-over. |
| Calendrier prévisionnel | Autorisation CPP et ANSM : 31 juillet 2016  Début des inclusions et de l’étude : 01 août 2016  Fin des inclusions : 30 septembre 2016  Fin de l’étude : 30 novembre 2016 |
| méthode statistique | L'étude étant croisée, l'effet carry-over sera testé afin de tester l’interaction entre les deux prothèses en utilisant un test de Student apparié. Si l'effet est significatif, seule la première période sera analysée en utilisant un test de Student ou le test non paramétrique de Wilcoxon si les hypothèses de normalité et d'égalité des variances ne sont pas vérifiées. Si l'effet est non significatif, l'effet période et l'effet prothèse pourront ensuite être testés en utilisant un test de Student apparié. |
| Retombées attendues | Les résultats devraient mettre en évidence une diminution de l’abduction d’épaule, une diminution du temps passé avec une abduction d’épaule supérieure à 60° et une augmentation de la satisfaction des personnes appareillées avec une pince Axon-Hook, en comparaison avec la pince Greifer.  Il n’est pas attendu de différence significative sur la dextérité manuelle. |

# Dispositif médical à l’essai

## Description générale du dispositif

Axon-Hook est une pince myoélectrique composée d’une tige latérale fixe et d’une tige médiane mobile, toutes deux en forme de crochet pour assurer la préhension des objets selon un mouvement angulaire. Le fait qu’une seule des deux tiges soit mobile apporte de la précision à la préhension puisque l’utilisateur va positionner la tige fixe contre l’objet à saisir puis simplement effectuer le mouvement de commande de la fermeture pour que la pince se referme. Les tiges sont en titane et sont recouvertes d’un revêtement en polyuréthane pour assurer une bonne adhérence des prises. Leur faible encombrement permet à l’utilisateur de bien voir ses prises.

Axon-Hook dispose également d’un poignet flexible orientable et verrouillable en flexion / extension afin de positionner la pince de façon optimale par rapport à l’objet à saisir. Il est recouvert d’une manchette de protection. Le positionnement et le verrouillage / déverrouillage est assuré par le membre controlatéral. En fonction des souhaits et capacités de commande du patient, la rotation du poignet peut être passive ou motorisée.

La commande de la pince Axon-Hook est myoélectrique et l’alimentation en énergie du produit est assurée par la batterie intégrée dans l’emboiture. Ce dispositif de préhension fait partie du système de prothèse modulaire Axon-Bus d’Otto Bock.

## Destination prévue du dispositif

Axon-hook est exclusivement destiné à l’appareillage exoprothétique des membres supérieurs.

## Objectif prévu dans l'investigation clinique

Axon-hook doit permettre à son utilisateur de limiter les mouvements d’abduction de l’épaule lors de déplacement d’objets, d’améliorer sa dextérité manuelle et d’augmenter sa satisfaction.

## Population et indications

**Population**

Selon les données du PMSI pour l’année 2014, il y a eu 147 cas d’amputation du membre supérieur pour les niveaux compris entre l’amputation trans-radiale et la désarticulation d’épaule.

Une pince est une adjonction que le patient peut fixer sur sa prothèse à la place de sa main prothétique pour réaliser des activités professionnelles ou de loisir difficilement ou non réalisable avec une main morphologique. Axon-Hook utilise un système électronique Axon-Bus, compatible avec l’électronique de la main morphologique Michelangelo. Selon l’avis de la CNEDiMTS du 12 janvier 2016, un maximum de 260 prothèses myoélectriques Michelangelo pourraient être adaptées par an en France, en première mise, deuxième mise ou renouvellement. On peut estimer la population cible pour la pince Axon-Hook à 20% de la population cible de Michelangelo, soit environ 50 cas par an.

**Indications**

Axon-hook peut être utilisé dans le cas d’amputations unilatérales ou bilatérales à partir d’une hauteur d’amputation de niveau transradial ou transhuméral ou en cas de dysmélie pour des appareillages de l’avant-bras ou du bras.

## Instructions de mise en place et d’utilisation du dispositif

Instructions de mise en place

Les réglages de la pince Axon-Hook sont personnalisés à l’aide d’un ordinateur et du logiciel Axon-Soft II en fonction du profil et des capacités de commande du patient. Seul un orthoprothésiste ayant suivi une formation délivrée par Otto Bock sur ce dispositif est habilité à le mettre en place et à procéder aux réglages de la prothèse. L’attribution d’une pince Axon-hook doit se faire dans le cadre d’un suivi par un médecin responsable d’appareillage disposant de moyens techniques et humains permettant d’assurer un apprentissage et un suivi médicotechnique approprié pour ce type d’appareillage.

Instructions d’utilisation

La prothèse munie de la pince Axon-Hook a été conçu pour les activités de la vie quotidienne. Elle est indiquée pour la réalisation d’activités manuelles à titre professionnel ou de loisirs. Elle doit être rechargée quotidiennement. Les limites d’utilisation de la prothèse sont décrites dans la notice d’utilisation destinée à l’utilisateur.

## Contre-indications

Aucune contre-indication.

## Fabricant et distribution

Axon-hook est fabriqué par le groupe Otto Bock Healthcare et commercialisé en France par Otto Bock France. Ce dispositif est vendu exclusivement aux orthoprothésistes et aux centres de rééducation spécialisés en appareillage.

## Identification et traçabilité

Identification et traçabilité du dispositif

Chaque pince Axon-hook est identifiée par sa référence « 8E600 » ainsi que par un numéro de série unique. Ses réglages nécessitent l’utilisation du logiciel Axon-Soft 560X500.

Identification et traçabilité du dispositif dans le cadre de l’investigation clinique

Chaque dispositif Axon-hook utilisé dans le cadre de l’investigation clinique sera étiqueté « Etude Axon-Hook - Dispositif d’investigation ». Sa traçabilité sera assurée grâce à son numéro de série.

# Justification de la conception de l'investigation clinique

## Description de la prise en charge existante et de ses limites

Le dispositif Axon-Hook n’est actuellement pas pris en charge par la sécurité sociale.

## Résultats des essais disponibles et pertinents au regard de l’étude

Il n’existe aucune étude spécifique à la pince Axon-Hook.

# Risques et bénéfices du dispositif médical à l’essai et de l'investigation clinique

## Avantages cliniques attendus

Grâce aux spécificités techniques d’Axon-Hook et plus particulièrement à son poignet orientable et verrouillable en flexion/extension et au faible encombrement du dispositif, il est attendu que les mouvements d’abduction de l’épaule soient limités lors de la préhension d’objets et que celle-ci soit facilitée.

La diminution des mouvements de compensation lors de la manipulation d’objets devrait diminuer la fatigue et encourager l’utilisation de la prothèse au quotidien.

## Risques

Axon-Hook est un dispositif médical de classe I marqué CE. Son utilisation dans le cadre de l’investigation sera conforme aux conditions définies dans le cadre du marquage CE. Axon-Hook ne présente pas de risque particulier.

## Étapes entreprises pour maîtriser ou atténuer les risques.

Les sujets participant à l’étude seront informés sur les conditions d’utilisation du dispositif par oral et par écrit lors de la mise en place du dispositif. Le sujet bénéficiera d’au moins une séance de rééducation par un membre de l’équipe thérapeutique afin qu’il intègre les fonctionnalités de ce dispositif et qu’il en ait la maîtrise.

## Justification du rapport bénéfice/risque.

Au regard des faibles risques énoncés précédemment, l’étude pourrait permettre de mettre en évidence l’amélioration du service rendu avec la pince Axon-Hook et de justifier une prise en charge pour les patients.

# Objectifs et hypothèses de l'investigation clinique

## Objectifs principaux et secondaires

### Objectif principal

L’objectif principal est de comparer les mouvements d’abduction de l’épaule lors de déplacements d’objets avec la pince Axon-Hook et la pince Greifer chez des sujets amputés transradio-ulnaire utilisateurs d’une prothèse myoélectrique.

### Objectifs secondaires

Les objectifs secondaires sont de comparer le pourcentage de temps passé avec une abduction d’épaule supérieure à 60° lors du test Box and Blocks, la dextérité manuelle et la satisfaction avec la pince Axon-Hook et la pince Greifer chez des sujets amputés transradio-ulnaire utilisateurs d’une prothèse myoélectrique.

## Hypothèses principales et secondaires

### Hypothèse principale

L’hypothèse principale est que la pince Axon-Hook permet de diminuer les mouvements de compensation lors du déplacement d’objets, nota ment grâce à son poignet flexible et orientable. Ces mouvements de compensation sont mesurables en laboratoire d’analyse du mouvement au travers de l’abduction moyenne de l’épaule lors du déplacement des cubes du test Box and Blocks, en comparaison de la pince Greifer.

### Hypothèses secondaires

Les hypothèses secondaires sont que la pince Axon-Hook permet de diminuer le temps passé avec une abduction de l’épaule supérieure à 60° sur la durée du test Box and Blocks et d’augmenter la satisfaction, en comparaison avec la pince Greifer. Il n’est en revanche pas attendu de différence significative en termes de dextérité manuelle avec la pince Axon-hook et la pince Greifer.

# Conception de l'investigation clinique

## Critères d’évaluation principaux et secondaires

### Critère d’évaluation principal

Abduction moyenne de l’épaule lors du test Box and Blocks

Le critère d’évaluation principal est l’abduction moyenne de l’épaule lors de la passation du test de dextérité manuelle Bock and Blocks, soit sur une durée de 60 secondes. Cette mesure est obtenue à partir de l’analyse des données acquises dans un laboratoire du mouvement.

Le test Box and Blocks a été retenu car il est largement cité dans la littérature pour l’évaluation des sujets amputés de membre supérieur. Pour ce test, le sujet est assis à une table et fait face à une boite rectangulaire qui est divisée en deux compartiments d’égales dimensions par le moyen d’une séparation. Le test consiste à déplacer des cubes un par un d’un compartiment à l’autre de la boite. Plus le nombre de cubes déplacées est important, meilleure est la dextérité manuelle. Ce test de répétition de déplacement d’objets est cohérent avec les tâches qui peuvent être réalisées avec une pince myoélectrique et est compatible avec des acquisitions en laboratoire du mouvement.

Lors de la première acquisition en laboratoire du mouvement, le sujet est tout d’abord évalué côté controlatéral au côté sous investigation (c’est-à-dire côté sain en cas d’amputation unilatérale). Les acquisitions se font ensuite avec le dispositif Axon-Hook et le Greifer, selon l’ordre défini par la randomisation.

### Critères d’évaluation secondaires

Pourcentage de temps passé avec une abduction d’épaule supérieure ou égale à 60°

Un des critères d’évaluation secondaire est le pourcentage de temps passé avec une abduction d’épaule supérieure ou égale à 60° lors de la passation du test de dextérité manuelle Bock and Blocks, soit sur une durée de 60 secondes. Cette mesure est obtenue à partir de l’analyse des données acquises dans un laboratoire du mouvement.

Test Box and Blocks

Le critère d’évaluation de la dextérité manuelle est le nombre de cubes délacés par le sujet en une minute lors de la passation du test Box and Blocks.

Questionnaire de satisfaction ESAT

Le critère d’évaluation de la satisfaction de l’usager est le score obtenu à l’auto-questionnaire ESAT, version française du QUEST validé pour les amputés. Le questionnaire ESAT contient une partie d’évaluation de la technologie du dispositif sur 8 items et une partie d’évaluation des services autour du dispositif sur 4 items. Il permet au patient de s’exprimer sur les critères qui sont les plus importants pour lui. Il permet également de calculer une valeur moyenne de satisfaction sur une échelle de 0 à 5, et une répartition des patients « Pas satisfaits du tout », « Peu satisfaits », « plus ou moins satisfaits », « Assez satisfaits » ou « Très satisfaits ».

Le score final de l’ESAT est la moyenne obtenue sur la totalité des 12 items.

## Plan expérimental

Il s’agit d’un essai comparatif de supériorité, randomisé, croisé, réalisé en ouvert, où chaque patient est son propre témoin.

Schéma de l’étude

Inclusion

GREIFER

Appareillage et rééducation

Randomisation

AXON-HOOK

Appareillage et rééducation

GREIFER

Appareillage et rééducation

GREIFER

Evaluation

GREIFER

Evaluation

AXON-HOOK

Evaluation

T1 + 2 semaines

T0

T1

T1

AXON-HOOK

Appareillage et rééducation

AXON-HOOK

Evaluation

T1 + 2 semaines

T2

T2 + 2 semaines

T2

T2 + 2 semaines

Essai réalisé en ouvert

L’étude ne peut se faire ni en double aveugle ni en simple aveugle. Le dispositif étant intégré au niveau de l’extrémité distale de la prothèse et ayant un rôle de préhension, il n’est pas possible de le cacher au patient ou à l’investigateur tout au long de l’étude.

Durées de suivi

Compte tenu de la facilité d’utilisation des pinces, la durée de suivi est de deux semaines minimum avec chaque dispositif, avec une tolérance de deux semaines supplémentaires pour pouvoir fixer la date de visite d’évaluation. Lors du changement de dispositif, la ou les séances de rééducation feront également office de « wash out ».

Appareillage

Afin de n’évaluer que l’effet pince, pour un même patient, le choix d’une prono-supination passive ou motorisée devra être le même pour les deux dispositifs.

Rééducation

Les sujets inclus dans l’étude sont des utilisateurs réguliers d’une prothèse myoélectrique et qui en ont la maîtrise. L’objectif de la rééducation est donc de familiariser le sujet aux spécificités techniques et à l’utilisation du dispositif. Ainsi, la personne suit au minimum une séance de rééducation avec chaque dispositif. Le nombre de séances peut cependant être adapté en fonction des besoins individuels de chaque personne.

Evènements indésirables

Dans le cas d’une défaillance technique de l’appareillage ou d’un évènement empêchant l’utilisation du dispositif pendant plusieurs jours, la date de fin de période de suivi sera reportée d’autant de jours afin de respecter les deux semaines de suivi prévues.

En cas de défaillance technique du dispositif évalué, il sera remplacé par un dispositif strictement identique et possédant strictement les mêmes réglages de fonctionnement.

## Dispositif médical à l’essai et comparateur

Dispositif médical à l’essai : Axon-Hook Otto Bock

Comparateur : Greifer Otto Bock

### Description de l'exposition au dispositif médical d’essai et au comparateur.

Les pinces Axon-Hook et Greifer sont des outils non-morphologiques utilisés en alternative de la main myoélectrique pour réaliser certaines activités. La fréquence et durée d’utilisation des dispositifs est donc variable d’un sujet à l’autre, en fonction du projet professionnel ou du projet de vie du sujet.

### Justification du choix du comparateur.

Le comparateur est la pince Greifer, qui est inscrite à la LPPR sous nom de marque dans le système Myobock d’Otto Bock depuis 2007. Ce système, largement prescrit en France, induit logiquement la pince Greifer comme comparateur de l’étude.

## Population étudiée

### Critères d'inclusion

- personnes amputées du membre supérieur au niveau transradio ulnaire
- personnes dont l’amputation est acquise ou congénitale
- personnes utilisatrices régulières d’une prothèse myoélectrique et en ayant la maîtrise
- personnes dont le membre résiduel est stabilisé et qui sont au minimum à six mois de l’amputation
- personnes dont l’activité professionnelle ou le projet de vie justifie ou pourrait justifier de l’utilisation d’une pince myoélectrique.
- personnes ayant donné par écrit leur consentement libre et éclairé

### Critères de non inclusion

- personnes de moins de 18 ans
- femmes enceintes
- personnes en situation d’urgence
- personnes incapables de donner personnellement leur consentement
- personnes dans l’incapacité psychique ou linguistique de comprendre les instructions de passation des tests de la recherche
- personnes non disponibles pour respecter la totalité du protocole d’étude

## Déroulement pratique de l’investigation clinique

### Modalités de recrutement

Le recrutement des patients se fera sur une période d’inclusion au cours de laquelle tous les patients suivis par le centre et répondant aux critères d’inclusion se verront proposer de participer à l’étude.

### Modalités d’information et de recueil du consentement

Lors de la visite d’inclusion, le médecin investigateur délivre au patient une information orale et écrite (annexe 1) expliquant l’objet, le déroulement et les risques liés à l’étude. Il s’assure que la personne répond bien aux critères d’inclusion et il s’assure également de la bonne compréhension de ces informations de la part de son patient.

Le patient peut bénéficier d’un délai de réflexion avant de donner son consentement. Dans le cas où il souhaite bénéficier de ce délai de réflexion, le médecin planifiera une nouvelle visite d’inclusion pour recueillir le consentement du patient par écrit à l’aide du formulaire (annexe 2). Il conserve un original dans le dossier patient et remet une copie au patient.

Toute personne ayant effectuée la visite d’inclusion, y compris les sujets non-inclus, feront l’objet d’une description.

### Méthodes de randomisation

La randomisation est faite par l’intermédiaire d’enveloppes scellées contenant chacune un ordre d’évaluation des dispositifs : 4 enveloppes mentionnent l’ordre d’essai « Axon-Hook puis Greifer » et 4 autres enveloppes mentionnent l’ordre d’essai « Greifer puis Axon-Hook ». Lors de la visite d’inclusion et après obtention de l’accord de participation du patient, l’investigateur tir au sort une enveloppe afin de définir l’ordre d’essai des dispositifs.

### Appareillage et rééducation

Appareillage

L’appareillage est réalisé par un orthoprothésiste habilité par Otto Bock à la mise en place du dispositif Axon-Hook. Celui-ci s’assure du confort et de la bonne adaptation des prothèses à l’essai et procède aux réglages personnalisés pour le patient. Afin de n’évaluer que l’effet pince, pour un même patient, le choix d’une prono-supination passive ou motorisée est le même pour les deux dispositifs. Les emboitures sont identiques pour chaque prothèse à l’essai.

Rééducation

Dans la semaine qui suit chaque appareillage, le patient suit au minimum une séance de rééducation. Celle-ci a pour objectif de s’assurer qu’il connaît les fonctionnalités de chaque dispositif et qu’il en a une parfaite maîtrise. Si nécessaire, des séances additionnelles sont proposées. Le nombre total de séances effectuées est reporté par l’investigateur sur le cahier d’observation.

### Équipement nécessaire à l’investigation clinique

Appareillage

L’orthoprothésiste doit disposer des logiciels Paula et AxonSoft 2 pour procéder aux réglages des prothèses avec les pinces Greifer et Axon-Hook.

Rééducation

Aucun équipement spécifique en dehors de celui habituellement utilisé par les équipes de rééducation n’est nécessaire dans le cadre de cette investigation clinique.

Analyse du mouvement

L’analyse des amplitudes d’abduction de l’épaule s’effectue dans un laboratoire d’analyse du mouvement. Le système retenu pour cette exploration fonctionnelle est le système d’analyse du mouvement en 3 dimensions VICON® (© 2007 Vicon Motion Systems Limited) qui comprend 2 caméras Basler  pour la capture de vidéos selon 2 plans différents et de 9 caméras opto-électroniques qui utilisent la lumière infra-rouge pour enregistrer les trajectoires de marqueurs positionnés sur des points suivants :

| Label | Nom du marqueur | Positionnement |
| --- | --- | --- |
| Marqueurs sur le tronc | | |
| LFHD / RFHD | Tête avant | A l’avant de la tête, au niveau des tempes |
| LBHD / RBHD | Tête arrière | A l’arrière de la tête |
| C7 | C7 | Apophyse épineuse de la 7^ème^ vertèbre cervicale |
| RBAK | Arrière droit | omoplate droite |
| T10 | T10 | Apophyse épineuse de la 10^ème^ vertèbre thoracique |
| CLAV | Clavicule | Echancrure jugulaire : point de rencontre entre la clavicule et le sternum |
| STRN | Sternum | Appendice xiphoïde |
| LASI / RASI | EIAS | Epines Iliaques Antéro-Supérieures |
| SACR | Sacrum | Au regard des fossettes au niveau du sacrum |
| Marqueurs sur chaque membre supérieur (L=Gauche ; R=Droite) | | |
| LSHO / RSHO | Epaule | Jonction acromio-claviculaire |
| LUPA / RUPA | Marqueur A du bras | Partie latérale du bras |
| LELB / RELB | Coude | Epicondyle latéral du coude, à proximité de l’axe articulaire |
| LFRM / RFRM | Avant-bras | Partie latérale de l’avant-bras |
| LWRA / RWRA | Marqueur A du poignet | Styloïde radiale, à proximité de l’axe articulaire |
| LWRB / RWRB | Marqueur B du poignet | Styloïde cubitale, à proximité de l’axe articulaire |
| LFIN / RFIN | Doigt | En-dessous du troisième métacarpien |
| Marqueurs au niveau du mobilier | | |
| TABLE 1 / TABLE 2 | Table | 4 marqueurs : un à chaque coin de la table |
| FR 1 / FR 2 | Tabouret | 2 marqueurs à l’arrière du tabouret, au niveau de l’assise |
| BOITE 1 / BOITE 2 / BOITE 3 | Box and Blocks | 3 marqueurs : deux sur les coins avant droit et gauche du Box and Blocks (BOITE 1 & 3), un sur le bord avant de la séparation des deux compartiments (BOITE 2) |

Le logiciel NEXUS®, développé par la société VICON®, analyse les données des caméras infrarouges et reconstruit la position de chaque marqueur dans les trois dimensions de l’espace. Il permet d’extraire les données enregistrées sous forme de tableur à raison de 100 images par secondes (100Hz). Les valeurs d’abduction de l’épaule correspondent à des mesures anatomiques calculées selon le modèle Plug-In Gait®.

Test Box and blocks

L’analyse du mouvement et l’évaluation de la dextérité manuelle nécessitent d’avoir l’outil Box and Blocks qui est un test normalisé. Ce test consiste à déplacer un maximum de cubes en une minute d’un compartiment à l’autre d’une boite positionnée sur le bord d’une table de hauteur standard, le sujet étant assis sur une chaise de hauteur standard, face à la boite. Les conditions d’évaluation sont similaires quel que soit le dispositif évalué.

### Sortie d’étude ou arrêt prématuré du suivi

Les sujets peuvent retirer leur consentement et demander à sortir de l’étude a n’importe quel moment et quelle qu’en soit la raison. En cas de sortie prématurée, l’investigateur documente les raisons de façon aussi complète que possible. L’investigateur peut interrompre temporairement ou définitivement la participation d’un sujet à l’étude pour toute raison qui servirait au mieux les intérêts du sujet, en particulier en cas d’évènements indésirables graves. En cas de sujet perdu de vue, l’investigateur doit mettre tout en œuvre pour reprendre contact avec la personne. Apres trois relances téléphoniques hebdomadaires, le sujet est enregistré comme perdu de vue. L’investigateur renseigne dans le cahier d’observation électronique la raison de sortie d’étude ou d’arrêt prématuré du suivi.

### Procédures de remplacement des sujets, le cas échéant

En cas de sortie d’étude ou d’arrêt prématuré du suivi d’un grand nombre de sujets, le promoteur peut demander à ce que de nouveaux sujets répondant aux critères d’inclusion soient inclus dans l’étude.

### Chronologie des visites

**Visite 1**

Inclusion du patient

- Information au patient

- Recueil du consentement

- Allocation du traitement :

Groupe Axon-Hook / Greifer

ou

Groupe Greifer / Axon-Hook

**Visite 2**

Appareillage Axon-Hook

Rééducation

PINCE AXON-HOOK

(2 semaines)

PINCE GREIFER

(2 semaines)

PINCE GREIFER

(2 semaines)

PINCE AXON-HOOK

(2 semaines)

**Visite 3**

**(2 semaines après la mise en place du DM)**

Evaluation Axon-Hook

- Test Box and Blocks au laboratoire du mouvement

- auto-questionnaire ESAT

**Visite 4**

Appareillage Greifer

Rééducation

**Visite 5**

**(2 semaines après la mise en place du DM)**

Evaluation Greifer

- test Box and Blocks au laboratoire du mouvement

- auto-questionnaire ESAT

- préférence du patient

**Visite 2**

Appareillage Greifer

Rééducation

**Visite 3**

**(2 semaines après la la mise en place du DM)**

Evaluation Greifer

- Test Box and Blocks au laboratoire du mouvement

- auto-questionnaire ESAT

**Visite 4**

Appareillage Axon-Hook

Rééducation

**Visite 4**

**(2 semaines après la mise en place du DM)**

Evaluation Axon-Hook

- test Box and Blocks au laboratoire du mouvement

- auto-questionnaire ESAT

- préférence du patient

### Durée de participation pour chaque sujet et durée totale de l'investigation clinique

Si l’on considère environ deux semaines d’essai pour chaque dispositif, la durée de participation pour chaque sujet est d’environ 4 semaines. La durée totale de l’investigation clinique est estimée à cinq mois.

## Gestion des évènements indésirables

### Définitions

- **Evénement indésirable (EvI)** : toute manifestation nocive survenant chez une personne qui se prête à une recherche biomédicale que cette manifestation soit liée ou non à la recherche ou au dispositif expérimental sur lequel porte cette recherche.
- **Evénement indésirable grave (EvIG)** : la gravité est définie par l’une des constatations suivantes :
  - Décès
  - Mise en jeu du pronostic vital (menace vitale immédiate, au moment de l’évènement, et ce, indépendamment des conséquences qu’aurait une thérapeutique correctrice ou palliative)
  - Incapacité ou handicap important ou durable
  - Hospitalisation
  - Prolongation d’hospitalisation
  - Malformation/anomalie congénitale
  - Evènement potentiellement grave (évènement clinique indésirable ou résultat de laboratoire à caractère grave ou considéré comme tel par l’investigateur)
- **Effet indésirable (EI)** : toute réaction nocive et non désirée liée à un dispositif expérimental ou tout incident qui aurait pu entraîner cette réaction si une action appropriée n’avait pas été effectuée, chez une personne qui se prête à la recherche ou chez l’utilisateur du dispositif médical.
- **Effet indésirable grave (EIG)** : évènement indésirable grave imputable à un dispositif expérimental.
- **Effet indésirable inattendu** : effet indésirable dont la nature, la sévérité, l’intensité ou l’évolution ne concorde pas avec les informations figurant dans la notice d’instruction ou la notice d’utilisation lorsqu’il fait l’objet d’un marquage CE, et dans le protocole ou la brochure pour l’investigateur lorsqu’il ne fait pas l’objet d’un tel marquage.
- **Imputabilité** : relation entre l’EvI et le dispositif de l’étude. L’EvI lié au dispositif expérimental deviendra un EI. Les facteurs à prendre en compte pour la détermination de l’imputabilité sont :
  - la chronologie des évènements,
  - la disparition de l’EvI lors du retrait du dispositif et/ou la réapparition en cas de nouvelle pose,
  - la notion d’antécédent d'évènement similaire lors de l'utilisation du dispositif ou d’un dispositif de la même classe,
  - l’existence d’une autre étiologie.
- **Intensité**: l’intensité des EvI est évaluée par l’investigateur selon la classification suivante :
  - léger de grade 1 : EvI généralement transitoire et sans retentissement sur les activités normales,
  - modéré de grade 2 : EvI suffisamment gênant pour retentir sur les activités normales,
  - sévère de grade 3 : EvI modifiant considérablement le cours normal des activités du patient, ou invalidant, ou constituant une menace pour la vie du patient.

Remarque : le critère d’intensité ne doit pas être confondu avec le critère de gravité qui sert de guide pour définir les obligations de déclaration.

### Liste des événements indésirables prévisibles et des effets indésirables attendus du dispositif, leur incidence probable, le moyen d'atténuation ou le traitement

Evènements indésirables prévisibles :

- Blessure
- Maladie
- Hospitalisation
- Problème d’adaptation ou de réglages de la prothèse
- Dysfonctionnement de la prothèse
- Panne de la prothèse
- Casse de la prothèse

Effets indésirables attendus du dispositif :

Aucun effet indésirable attendu du dispositif

### Rôle de l’investigateur

Pendant toute la durée de suivi des patients, tout EvI qui surviendrait pour une personne dans le cadre de l’essai doit être suivi jusqu’à sa résolution, ou jusqu’à ce qu’il soit jugé permanent. La survenue d’un EvI doit être recherchée par l’investigateur à chaque visite de suivi.

Tout EvI, lié ou non à l’essai, doit être documenté dans la fiche prévue à cet effet du cahier d’observation. Tout changement dans la sévérité, la relation avec le dispositif médical, les interventions requises pour traiter l’évènement ou l’évolution devra être surveillée et documentée.

Tout EvIG, quelle que soit sa relation de causalité avec le dispositif de l’essai doit être impérativement déclaré auprès du promoteur dans la fiche prévue à cet effet du cahier d’observation dans les 24 heures de sa survenue (ou dès que le médecin investigateur en a connaissance). La déclaration initiale peut être suivie de compléments d’informations pertinentes dans les 8 jours en cas d’évènement fatal ou menaçant le pronostic vital et dans les 15 jours dans les autres cas.

### Rôle du promoteur

Le promoteur évalue la causalité des EvI (tous les évènements indésirables, pour lesquels l'investigateur ou le promoteur estime qu'une relation de causalité avec le dispositif expérimental peut être raisonnablement envisagée, sont considérés comme des suspicions d'effets indésirables. En cas d'évaluation différente du promoteur et de l'investigateur, les deux avis sont mentionnés sur la déclaration adressée à l'autorité compétente si cette déclaration est nécessaire),

Le promoteur déclare à l’ANSM et au CPP :

- tous les EIG et inattendus susceptibles d’être dus à un DM faisant l’objet de la recherche
- tous les EvIG pouvant être liés au geste de mise en œuvre du dispositif médical.

La déclaration réglementaire est faite dans un délai maximum de :

- 7 jours calendaires dans le cas des EIG inattendus et d’EvIG pouvant être lié au geste de mise en œuvre et ayant entraîné la mort ou la mise en danger. Dans ces cas, des informations complémentaires pertinentes doivent être recherchées et transmises dans un nouveau délai de 8 jours.

- 15 jours calendaires pour tous les autres EG inattendus et EvIG pouvant être liés au geste de mise en œuvre du dispositif médical. De même des informations complémentaires pertinentes doivent être recherchées et transmises dans un nouveau délai de 15 jours.

Les cahiers d’observation des évènements indésirables de tous les EvI notifiés par les investigateurs pourront être transmis à l’ANSM, à sa demande.

## Plan de surveillance

# Statistiques

## Taille de l'échantillon

Le calcul du nombre de patients à inclure est basé sur la différence attendu sur le critère principal, l’abduction moyenne de l’épaule lors de la passation du test Box and Blocks. Avec l’hypothèse d’une différence d’abduction moyenne de 30 ± 25 degrés il est nécessaire d’inclure 8 patients pour un essai randomisé réalisé en cross-over. Il est attendu une amélioration des critères secondaires mais pas une différence significative. Le nombre de patients est donc uniquement calculé sur la base du critère principal.

La formule de calcul utilisé dans le cas d’un essai randomisé en cross-over est la suivante :

n = (1-ρ) * 2 * (σ²/Δ²) * (z(α/2)-z(1-β))²

où,

α, = 0.05

1-β = puissance recherchée

σ = écart-type

Δ = différence attendue

ρ = coefficient de corrélation entre les réponses de chaque patient aux deux prothèses (si non connu, généralement estimé à 0.5)

Le nombre de patients nécessaire est basé sur différentes hypothèses de réduction des mouvements de compensation attendus avec la pince Axon-Hook en comparaison avec le Greifer :

| Hypothèses de différence attendue  (angle de réduction de l’abduction moyenne de l’épaule) | Ecart type commun | Nb. de patients requis |
| --- | --- | --- |
| 35 | 25 | 6 |
| 30 | 25 | 8 |
| 25 | 20 | 7 |

Compte tenu de la très faible prévalence de la population cible, il n’est pas envisageable d’inclure plus de 8 patients sur cette étude mono-centrique.

Il sera prévu de pouvoir inclure 8 patients.

### Traitement des données manquantes, inutilisées ou erronées

Aucun remplacement des données manquantes ne sera réalisé. En cas de données manquantes, illisibles ou incohérentes, des demandes d’informations complémentaires seront envoyées à l’investigateur concerné. ; les modifications seront ensuite intégrées au cahier d’observation par le médecin investigateur, avec mention de la date d’amendement de l’information et signature de l’investigateur dans la marge.

Si toutefois une donnée est manquante, le sujet concerné sera retiré de l’analyse du paramètre concerné.

### Populations d’analyse

Une analyse sur la population en intention de traiter (ITT) sera réalisée, puis sur la population per-protocole (PP). La population ITT est définie par tous les patients randomisés dans leur groupe de randomisation, quel que soit le traitement qu'ils ont effectivement reçu ou quel que soit leur devenir dans l'étude.

La population PP est définie par tous les patients de la population ITT sans déviation définie comme majeure.

### Analyses statistiques

Caractéristiques des patients

Les variables quantitatives seront résumées par les statistiques descriptives usuelles (moyenne, écart-type, médiane, minimum et maximum, 1er et 3ème quartile). Les variables qualitatives seront décrites par les effectifs et les fréquences. Les intervalles de confiance à 95% seront présentés lorsque cela sera pertinent. Les caractéristiques des patients à l'inclusion seront présentées et comparées dans chaque groupe afin de vérifier la comparabilité initiale des groupes. Les caractéristiques des patients non inclus et celles des perdus de vue seront étudiées.

Analyse des critères principal et secondaires

L'étude étant croisée, l'effet carry-over sera testé afin de tester l’interaction entre les deux prothèses en utilisant un test de Student apparié. Si l'effet est significatif, seule la première période sera analysée en utilisant un test de Student ou le test non paramétrique de Wilcoxon si les hypothèses de normalité et d'égalité des variances ne sont pas vérifiées.

Si l'effet est non significatif, l'effet période et l'effet prothèse pourront ensuite être testés en utilisant un test de Student apparié.

Analyse de la tolérance

L'ensemble des événements indésirables sera décrit dans les populations d'analyse et pour chacune des deux prothèses Axon-Hook et Greifer. De même, seront présentés un résumé des évènements indésirables possiblement liés à la prothèse et un résumé des évènements indésirables graves.

# Contrôle et assurance qualité

### Promoteur

La société Otto Bock est certifiée ISO 9001 et 13485. Les documents joints à l’étude rentrent dans le système qualité de l’entreprise.

### Collecte des données

La collecte des données sur le cahier d’observation est assurée par le médecin investigateur. A la fin du suivi de chaque patient, le médecin investigateur date, signe et appose le cachet du centre sur le cahier d’observation afin d’attester de l’exactitude des informations qui y sont mentionnées. Il transmet ensuite une copie de cahier d’observation au promoteur pour analyse des résultats et conserve l’original dans le dossier d’investigation de l’étude.

# Gestion des données

## CNIL

Le traitement des données se fera en conformité avec la Méthodologie de Référence n° MR-001 dont le formulaire de demande sera adressé à la Commission Nationale de l’Informatique et des libertés (CNIL) en France pour accord.

## Données recueillies

Les données sont recueillies au travers d’un cahier d’observation papier.

| Données recueillies par l’investigateur | Visite d’inclusion | Visite d’évaluation avec  Axon-Hook | Visite d’évaluation avec  Greifer |
| --- | --- | --- | --- |
| Caractéristiques du patient | X |  |  |
| Consentement éclairé du patient | X |  |  |
| Abduction moyenne de l’épaule sur la séquence du Box and Blocks |  | X | X |
| Pourcentage de temps passé avec une abduction d’épaule supérieure ou égale à 60° sur la séquence du Box and blocks |  | X | X |
| Test Box and Blocks |  | X | X |
| Questionnaire ESAT |  | X | X |
| Préférence du patient (après évaluation du dernier dispositif) |  | X | X |

Données recueillies par l’investigateur lors de la visite d’inclusion

- Initiales du patient
- Date de la visite d’inclusion
- Sexe : Homme / Femme
- Age
- Taille (cm)
- Poids (Kg)
- Activité professionnelle : étudiant / retraité / sans activité professionnelle / en activité professionnelle (préciser)
- Loisirs : aucun / (préciser)
- Côté amputé évalué : droit / gauche
- Niveau d’amputation : 1/3 supérieur d’avant-bras / 1/3 moyen d’avant-bras / 1/3 inférieur d’avant-bras
- Latéralité : droitier / gaucher
- Amputé depuis (date)
- Etiologie : traumatique / congénitale / vasculaire / tumorale / autre (préciser)
- Handicaps associés : aucun / amputation du membre supérieur controlatéral (préciser niveau) / autre (préciser)
- Appareillé en myoélectrique depuis (date)
- Main myoélectrique actuelle : Sensor speed / Digital twin / DMC Plus / Vari plus / Autre (précisez)
- Le patient a une pince Greifer : oui / non

Données recueillies par l’investigateur lors des visites d’évaluation et saisies sur le cahier d’observation

- Dispositif évalué : Greifer / Axon-Hook
- Date du début de la période d’essai
- Date de la visite d’évaluation
- Nombre de séances de rééducation réalisées (1 séance minimum : 1 / 2 / autre (préciser)
- Pronosupination : passive / motorisée
- Réglages de la pince choisi par le patient (degrés : inclinaison radiale (Greifer) / inclinaison cubitale (Greifer) / flexion (Axon-Hook) / extension (Axon-hook)
- Nombre de cubes déplacés en une minute au test Box and Blocks (côté évalué et côté opposé)
- Abduction moyenne de l’épaule sur la séquence du test Box and Blocks côté opposé (côté évalué et côté opposé)
- Pourcentage de temps passé avec une abduction d’épaule supérieure ou égale à 60° sur la séquence du test Box and Blocks (côté évalué et côté opposé)
- Questionnaire ESAT
- Préférence du patient : Greifer / Axon-Hook (lors de la dernière évaluation seulement)

Données recueillies par l’investigateur lors de la clôture de l’étude

- Date de la clôture
- Motif de la clôture : évaluation à terme / patient perdu de vue / autre (préciser)

## Enregistrement des données

Les données recueillies lors de la visite d’inclusion et des visites d’évaluation sont saisies par l’investigateur dans le cahier d’observation.

Pour le questionnaire ESAT, l’investigateur reporte sur le questionnaire son nom, les initiales du patient et mentionne si l’évaluation concerne la pince Axon-Hook ou la pince Greifer. Il remet ensuite au patient le questionnaire en lui demandant de répondre à toutes les questions. L’investigateur joint ensuite le questionnaire au cahier d’observation du patient.

### Archivage des données

A la fin de l’étude, une copie des données collectées par le médecin investigateur sont envoyées au promoteur pour analyse puis archivage pendant 15 ans. Le médecin investigateur conserve l’original de tous les cahiers d’observation.

# Considérations éthiques

### Comité de protection des personnes

Conformément à la réglementation en vigueur en France, le protocole sera soumis pour avis au Comité de Protection des Personnes (CPP) et l’essai clinique fera l’objet d’une demande d’autorisation auprès de l’Agence Nationale de Sécurité du Médicament et des produits de santé (ANSM). Toute modification substantielle du protocole fera également l’objet d’une demande d’avis contraignant auprès du Comité de protection des personnes (CPP) et/ou à une demande d’autorisation auprès de l’ANSM. Aucune inclusion n’aura lieu avant l’obtention de l’autorisation de l’ANSM et de l’avis favorable du CPP, ainsi que de la visite de mise en place de l’étude par le promoteur.

### Information et consentement du patient

Les patients seront informés de façon complète et loyale, en des termes compréhensibles, des objectifs et des contraintes de l'étude, des risques éventuels encourus, des mesures de surveillance et de sécurité nécessaires, de leurs droits de refuser de participer à l'étude ou de la possibilité de se rétracter à tout moment.

Toutes ces informations figurent sur une note d’information et un formulaire de consentement remis au patient. Le consentement libre, éclairé et écrit du patient sera recueilli par l’investigateur avant l’inclusion dans l’étude. Une copie du formulaire d'information et de consentement signé par les deux parties sera remise au patient, l’investigateur en conservera l’original. Une copie sera placée en fin d’étude dans une enveloppe inviolable scellée regroupant l’ensemble des formulaires de consentement, celle-ci sera archivée par le promoteur.

### Indemnisation des sujets

La participation des sujets à l’étude ne donnera lieu à aucune indemnisation. En revanche, les frais de déplacements induits par la participation du sujet à l’étude seront couverts par le promoteur. Il faut alors utiliser le « formulaire de remboursement des frais de déplacement » qui est disponible auprès du médecin investigateur.

# Financement et assurances

### Financement

L’essai clinique est financé par le promoteur. Une convention tripartite entre Otto Bock France, l’investigateur et l’établissement de santé définit les conditions de prise en charge des prestations de l’investigation.

Les pinces Axon-Hook et Greifer nécessaires pour réaliser cette étude seront mis à disposition par le promoteur pour la durée de l’essai.

### Assurance

La société Otto Bock souscrira pour toute la durée de l'étude une assurance garantissant sa propre responsabilité civile ainsi que celle de tout médecin impliqué dans la réalisation de l'étude. Elle assurera également l'indemnisation intégrale des conséquences dommageables à la recherche pour la personne qui s'y prête et ses ayants droit, sauf preuve à sa charge que le dommage n'est pas imputable à sa faute ou à celle de tout intervenant, sans que puisse être opposé le fait d'un tiers ou le retrait volontaire de la personne qui avait initialement consenti à se prêter à la recherche.

### Amendements du plan d'investigation clinique

Le plan d’investigation clinique pourra faire l’objet d’un amendement si nécessaire.

Le promoteur devra informer le médecin investigateur que le protocole va être amendé. S’il le juge nécessaire, le promoteur peut suspendre l’investigation clinique le temps que le protocole ait été amendé. Cet amendement ne pourra être effectif qu’après approbation du CPP et de l’ANSM.

# Décompte des dispositifs

Le promoteur assurera la mise à disposition des dispositifs sous investigation, leur identification et leur attribution au centre investigateur. A l’issue de l’investigation clinique, il s’assurera que tous les dispositifs sous investigation lui ont été retournés.

# Arrêt prématuré ou suspension de l'investigation clinique

L’investigation clinique pourra être suspendue ou arrêtée prématurément pour l’ensemble de l’investigation sur demande expresse du promoteur.

Les critères justifiant cette décision sont les suivants:

- Le promoteur a constaté des écarts majeurs au protocole de la part de l’investigateur
- Le promoteur a identifié un risque potentiellement grave pour l’utilisateur, pour son environnement ou pour l’équipe d’investigation

En cas de suspension ou d’arrêt prématuré de l’investigation clinique, le promoteur informera l’investigateur dans les plus brefs délais et lui communiquera les dispositions à prendre. Il informera les autorités compétentes –CPP et ANSM- de la situation de l’investigation clinique.

# Confidentialité et publication des résultats

L’investigateur ainsi que toute personne amenée à collaborer à l’essai sont tenues au secret professionnel en ce qui concerne le dispositif médical utilisé, le protocole d’essai et les résultats.

Les données et résultats de l’essai sont la propriété d’Otto Bock. Les communications et rapports scientifiques correspondant à cette étude devront obligatoirement faire l’objet d’un accord préalable du promoteur.

# Calendrier prévisionnel de l’étude

Autorisation CPP et ANSM : 31 juillet 2016

Début des inclusions et de l’étude : 01 août 2016

Fin des inclusions : 30 septembre 2016

Fin de l’étude : 30 novembre 2016

# Bibliographie

*[1] The Psychosocial and Biomechanical Assessment of Amputees Fitted with Commercial Multi-grip Prosthetic Hands. A.G. Cutti, I. Parel, M. Luchetti, E. Gruppioni, N.C. Rossi c and G. Verni. Grasping the Future: Advances in Powered Upper Limb Prosthetics, 2012, 59-77.*

*[2]* [*Major MJ*](http://www.ncbi.nlm.nih.gov/pubmed/?term=Major%20MJ%5BAuthor%5D&cauthor=true&cauthor_uid=25192744)*1,* [*Stine RL*](http://www.ncbi.nlm.nih.gov/pubmed/?term=Stine%20RL%5BAuthor%5D&cauthor=true&cauthor_uid=25192744)*,* [*Heckathorne CW*](http://www.ncbi.nlm.nih.gov/pubmed/?term=Heckathorne%20CW%5BAuthor%5D&cauthor=true&cauthor_uid=25192744)*,* [*Fatone S*](http://www.ncbi.nlm.nih.gov/pubmed/?term=Fatone%20S%5BAuthor%5D&cauthor=true&cauthor_uid=25192744)*,* [*Gard SA*](http://www.ncbi.nlm.nih.gov/pubmed/?term=Gard%20SA%5BAuthor%5D&cauthor=true&cauthor_uid=25192744)*: Comparison of range-of-motion and variability in upper body movements between transradial prosthesis users and able-bodied controls when executing goal-oriented tasks.*[*J Neuroeng Rehabil.*](http://www.ncbi.nlm.nih.gov/pubmed/25192744) *2014 Sep 6;11:132.*

*[3]* *Hebert JS, Lewicke JJ. Case report of modified Box and Blocks test with motion capture to measure prosthetic function. J Rehabil Res Dev. 2012;49(8):1163–74.*

*[4] Hebert JS, Lewicke JJ. Normative data for modified Box and Blocks test measuring upper-limb function via motion capture. J Rehabil Res Dev. 2014;51(6):919-932*

*[5] Ostlie K, Franklin RJ, Skjeldal OH, Skrondal A, Magnus P: Musculoskeletal pain and overuse syndromes in adult acquired major upper-limb amputees. Arch Phys Med Rehabil 2011, 92:1967–1973 e1961.*

*[6] Marisol A. Hanley, PhD, Dawn M. Ehde, PhD, Mark Jensen, PhD, Joseph Czerniecki, MD,Douglas G. Smith, MD, and Lawrence R. Robinson, MD. Chronic Pain Associated with Upper-Limb Loss. Am J Phys Med Rehabil. 2009 September ; 88(9): 742–779. doi:10.1097/PHM.0b013e3181b306ec*

*[7] Kontaxis A, Cutti AG, Johnson GR, Veeger HE (2009). A framework for the definition of standardized protocols for measuring upper-extremity kinematics. Clin Biomech (Bristol, Avon) 24(3):246-253.*

*[8] Wu G, van der Helm FC, Veeger HE, Makhsous M, Van Roy P, Anglin C, Nagels J, Karduna AR, McQuade K, Wang X, Werner FW, Buchholz B (2005) ISB recommendation on definitions of joint coordinate systems of various joints for the reporting of human joint motion–Part II: shoulder, elbow, wrist and hand. J Biomech 38(5):981–992*

*[9] Parel I, Cutti AG, Kraszewski, Verni G, Hillstrom H, Kontaxis A. Intra-protocol repeatability and inter-protocol agreement for the analysis of scapula-humeral coordination. Med Biol Eng Comput (2014) 52:271-282*

*[10] Demers, L., Weiss-Lambrou, R., & Ska, B. (1996). Development of the Quebec User Evaluation of Satisfaction with Assistive Technology (QUEST). Assistive Technology, 8, 3-13.*

*[11] Mathiowetz V, Volland G, Kashman N, Weber K. Adult norms for the Box and Block Test of manual dexterity. Am J Occup Ther. 1985;39(6):386–91.*

# Annexes

### Lettre d’information au sujet participant à l’étude


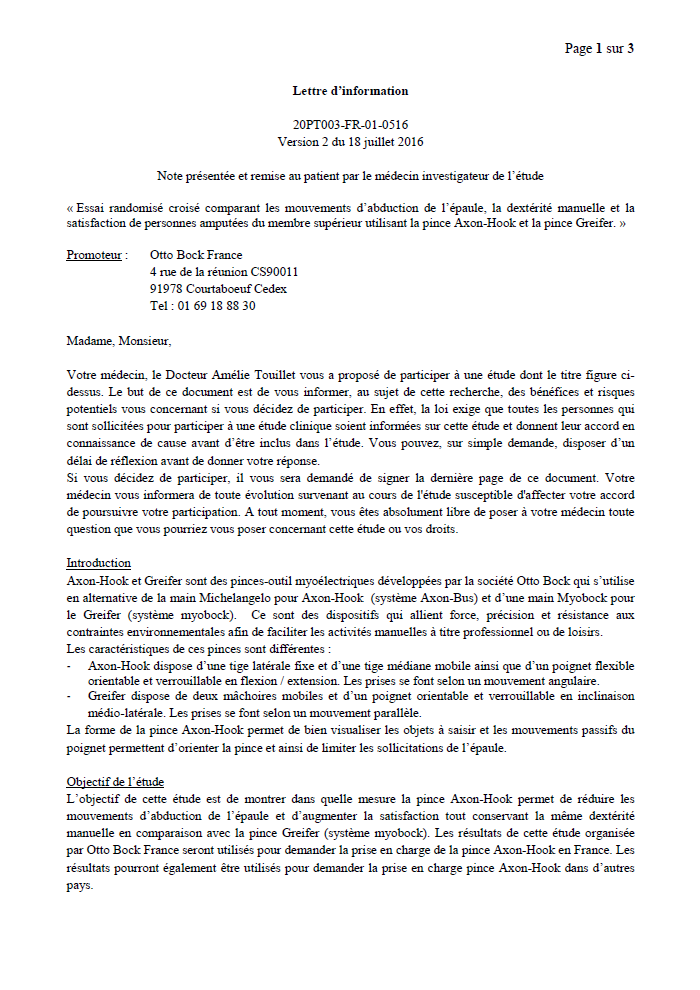


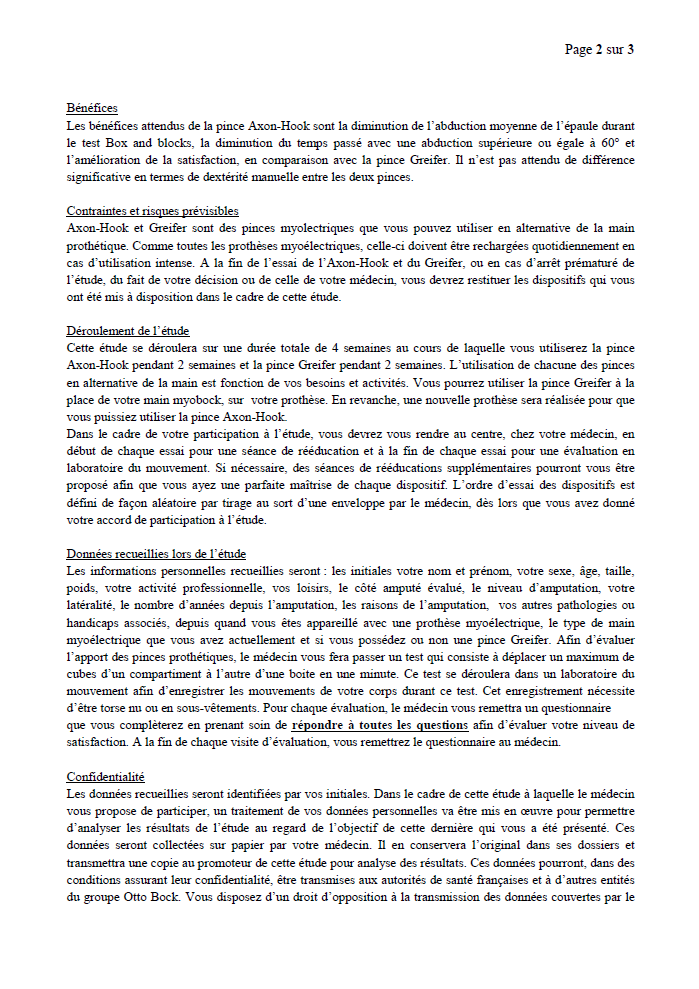


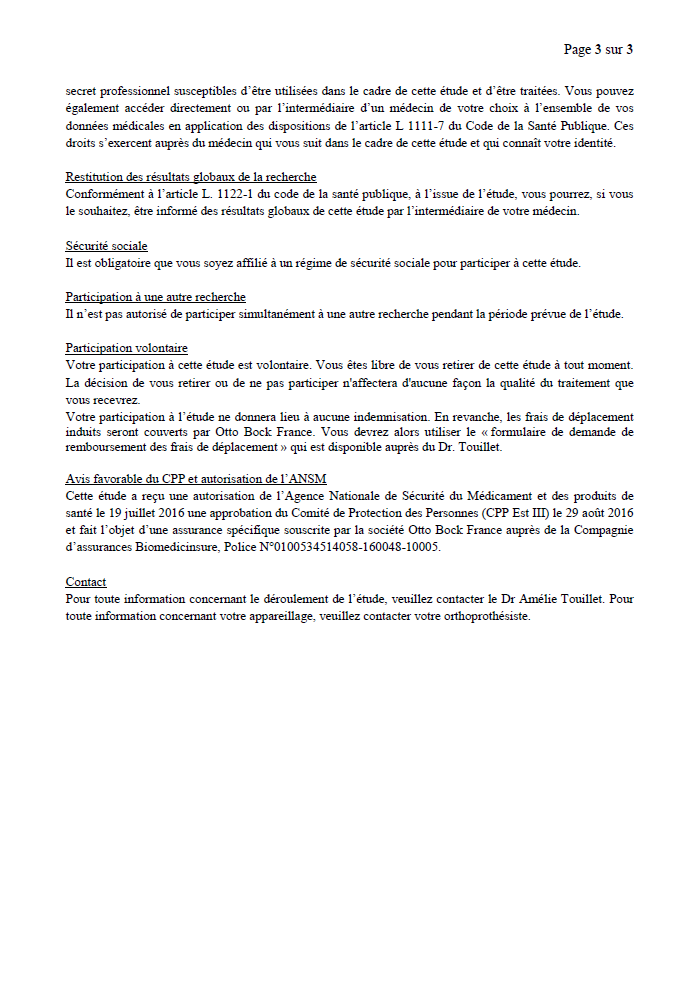


### Formulaire de consentement de la personne


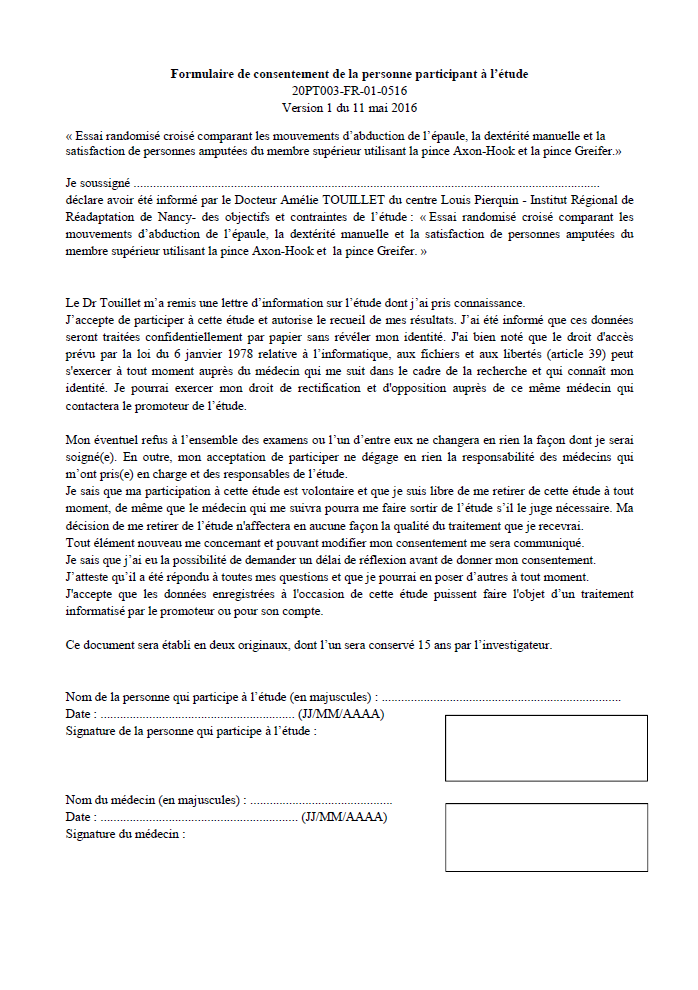


### Cahier d’observation


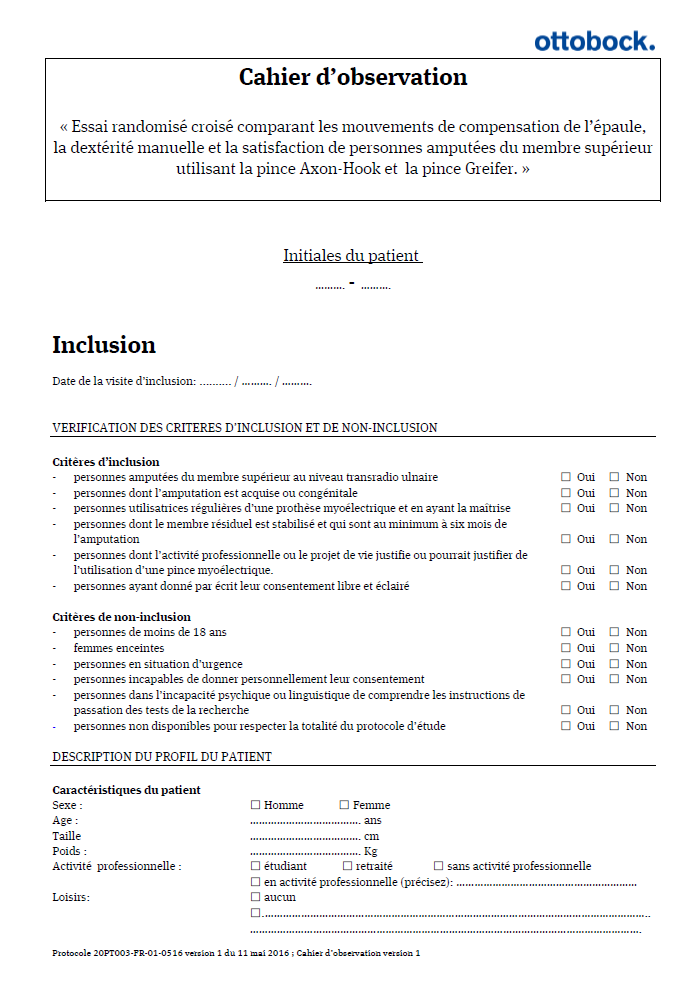


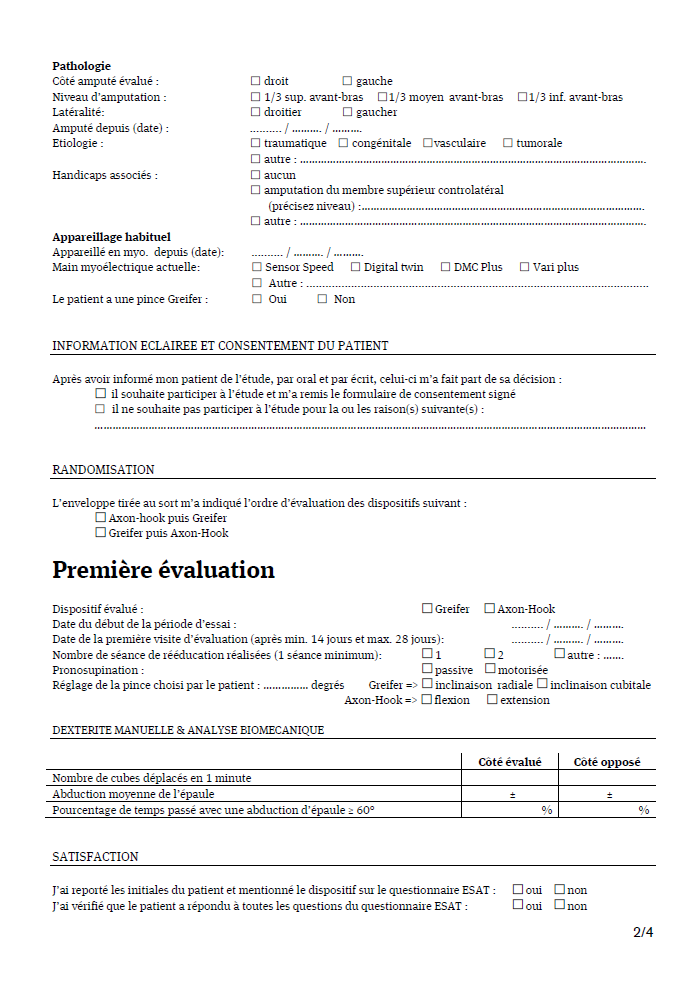


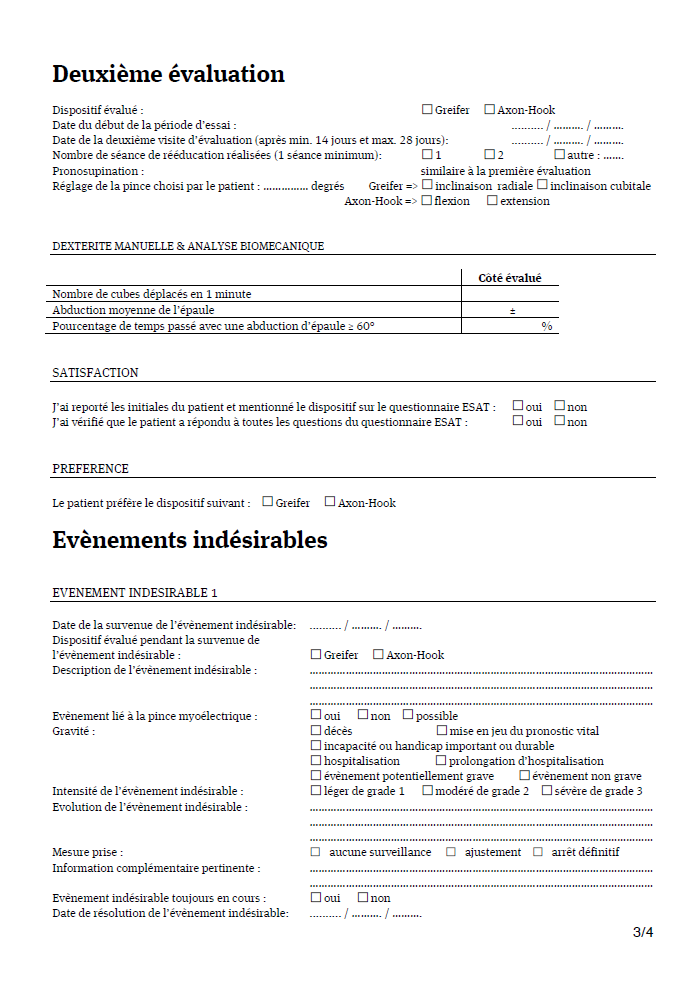


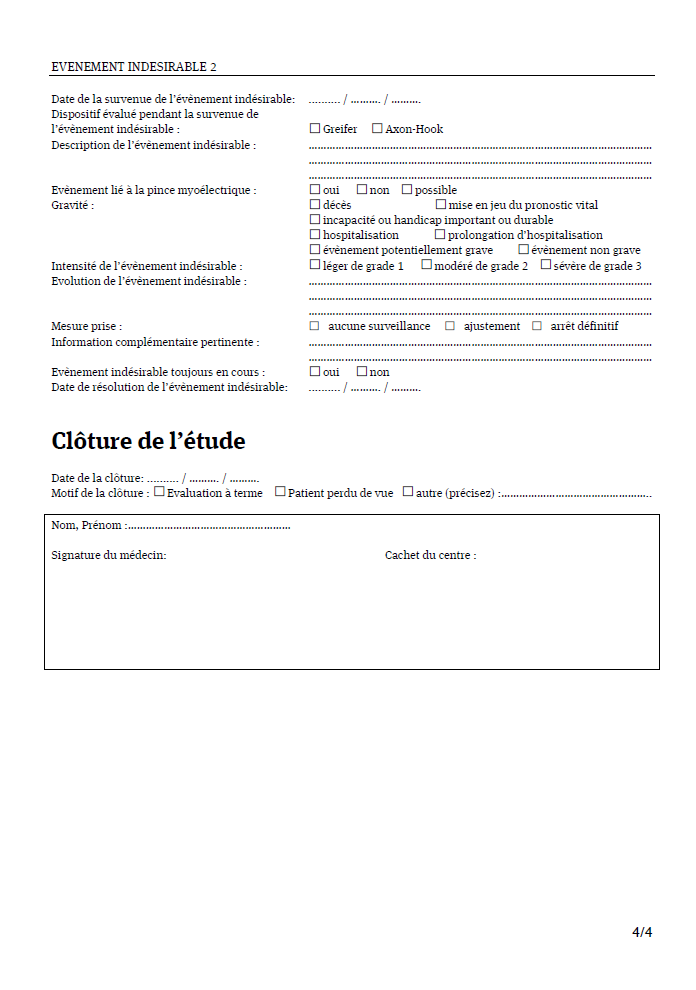


### Questionnaire de satisfaction du patient ESAT


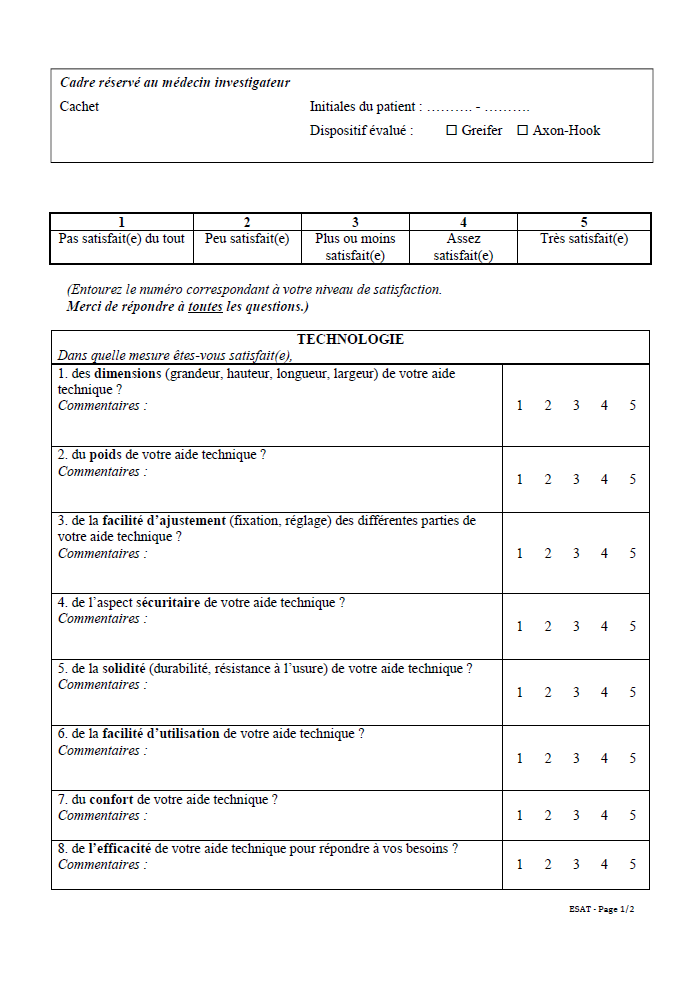


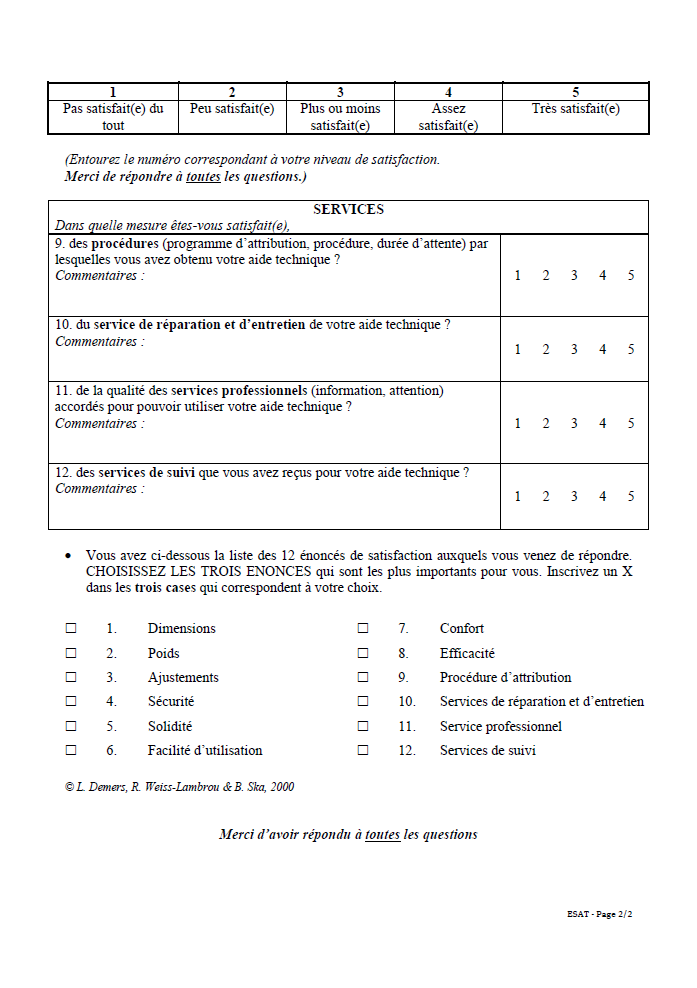


### Déclaration de conformité à la CNIL


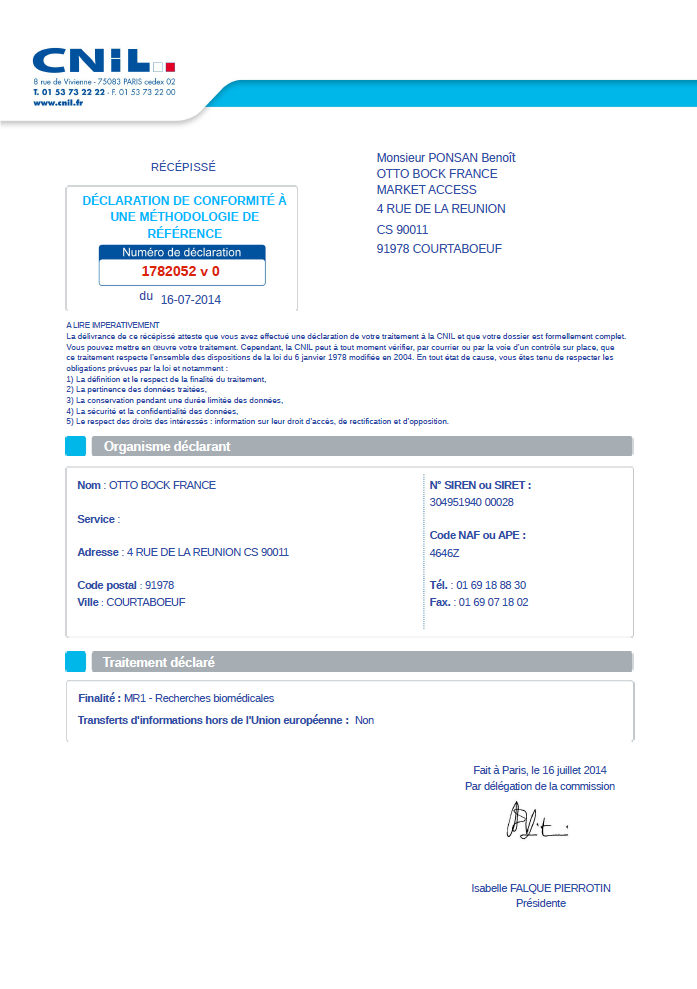

Supplement: S2 Protocol — (DOCX) [file pone.0272855.s003.docx]
