# Supplementary material for: Comparison of compensatory shoulder movements, functionality and satisfaction in transradial amputees fitted with two prosthetic myoelectric hooks
Source: PLoS One. 2023 Feb 2;18(2):e0272855. doi: 10.1371/journal.pone.0272855 (PMC9894487; doi:10.1371/journal.pone.0272855)
Supplement: Attachment — Submitted filename: Response to Reviewers_1.docx [file pone.0272855.s004.docx]

|   Centre Louis Pierquin  **75, Boulevard Lobau**  **54042 NANCY CEDEX**  Tél. : 03 83 52 67 50  Fax : 03 83 52 67 69  Direction Médicale  **Pr. J. PAYSANT, PU-PH**  Dr. M. BADIN, CCA  Dr. N. BERTONI, psychiatre  Centre de Nancy  **Dr. N. MARTINET**  Dr. B. CLAUDON  Dr. A. DATIE  Dr. A. FOISNEAU-LOTTIN  Dr. C. FRENAY  Dr. S. HENRY-CATALA  Dr. B. LASSALLE-KINIC  Dr. I. LOIRET  Dr. A. TOUILLET  Laboratoire du Mouvement  et Explorations  Pr. C. BEYAERT, PU-PH  Centre de Lay-Saint-Christophe  Dr. J-M. BEIS  Dr. M. BADIN  Dr. A. FRASER  Dr. H. GUESDON  Dr. N. KHALIL  Dr. L. LE CHAPELAIN  **Centre de Scy-Chazelles**  **Dr. I. DEDENON-MAYER**  Dr. S. DAGTEKIN  Dr. A. WALTER-KULL  Centre de Flavigny  **Dr. B. GIRARD**  Dr. J. BASTIEN  Dr. F. DALMONT  Dr. S. REGEF  CEM de Flavigny  E.R.E.A. de Flavigny  **D.I.M.**  Dr. M. DEGASPERI  Dr. L. LE CHAPELAIN  Pharmacie  Mme A-S. LAUSCH  Mme E. CONRARD  Mme H. OTTOMANI-MOUZE  **Cellule Recherche et Innovation**  J. PIERRET  **Réédoc**  C. GRANDIDIER | Constance Billon, PhD.  Service Explorations Fonctionnelles, Laboratoire du Mouvement  Centre Louis Pierquin, Institut Régional de Réadaptation, UGECAM  75 Boulevard Lobau, CS 34209 ; 54052 Nancy Cedex, France  Tel: +033383529811  [constance.billon@ugecam.assurance-maladie.fr](mailto:constance.billon@ugecam.assurance-maladie.fr)  Bijan Najafi  Academic Editor  PlosOne Journal  December 4, 2021  Editor’s suggestions:   1- Please ensure that your manuscript meets PLOS ONE's style requirements, including those for file naming.   *We have renamed figures at Fig1.tif.*  *We have placed the asterisk on the correct corresponding author.*   2- In your Methods section, please provide additional information about the participant recruitment method and the demographic details of your participants. Please ensure you have provided sufficient details to replicate the analyses such as a description of any exclusion criteria that were applied to participant recruitment.   *In [Methods/Participants] we have provided more details regarding the criteria for recruiting patients.*   3- Please ensure you have clearly identified primary, secondary, and any exploratory outcomes.   *In our opinion, the different types of results have been clearly specified.*   4- Please ensure your patient flow diagram is included as Figure 1 in the main manuscript. *We have changed Figure 1 so that it integrates the patients flow diagram.*   a. FZB(EO)19Febr21: Registration done retrospectively (after enrollment of participants)  b. FZB(EO)19Febr21: borderline ethics statement (IRB not named)  a. Thank you for submitting your clinical trial to PLOS ONE and for providing the name of the registry and the registration number. The information in the registry entry suggests that your trial was registered after patient recruitment began. PLOS ONE strongly encourages authors to register all trials before recruiting the first participant in a study.  As per the journal’s editorial policy, please include in the Methods section of your paper:  i) your reasons for your delay in registering this study (after enrolment of participants started);  *Registration on clinical trial is not a compulsory procedure. In France, the monitoring of studies is well supervised (CPP, CNIL, ANSM).* *As this study included few patients, it did not seem necessary to us to make this recording.* *We were not aware at the time of the importance of this clinical trial recording process.*  ii) confirmation that all related trials are registered by stating: “The authors confirm that all ongoing and related trials for this drug/intervention are registered”.  *We declared that.*  b. Thank you for including your ethics statement: "The study protocol was registered with the French National Agency for Medicines and Health Products (ANSM) and approved by an independent ethics committee (CPP Est-III) on August 29, 2016 (N°16.07.02). Data collection complied with the reference methodology for interventional trials (MR-001) issued by the French Data Protection Agency (CNIL).  The subjects were recruited during prosthetic medical appointments usually carried out at the rehabilitation center. During the inclusion period, all persons who met the inclusion criteria were invited to participate in the study. They received all the required oral and written information. All the subjects included in the trial signed the informed consent forms".  Please amend your current ethics statement to include the full name of the ethics committee/institutional review board(s) that approved your specific study.  Once you have amended this/these statement(s) in the Methods section of the manuscript, please add the same text to the “Ethics Statement” field of the submission form (via “Edit Submission”).  *We regularized that by writing the name of the committee in full.*   5- We note that you have stated that you will provide repository information for your data at acceptance. Should your manuscript be accepted for publication, we will hold it until you provide the relevant accession numbers or DOIs necessary to access your data. If you wish to make changes to your Data Availability statement, please describe these changes in your cover letter and we will update your Data Availability statement to reflect the information you provide.   *We will provide you with access to all data only after acceptance of the manuscript for publication.*   6- Please upload a new copy of Figure 3 as the detail is not clear. Please follow the link for more information.   *The quality of Figure 3 has been improved.*   7- Please include captions for your Supporting Information files at the end of your manuscript, and update any in-text citations to match accordingly. Please see our Supporting Information guidelines for more information.   *We edited these captions after [Conclusion].*  **Reviewer #1:**  Major revision: Testing the primary objectives with an all-inclusive linear mixed model would be superior to the analysis approach which has been taken. Mixed models offer the ability to test for differences in the effect of Greifer, Axon-Hook, and non-affected side. T-tests should not be needed. Additionally, specify the underlying covariance structure used in the mixed models and the criteria for selecting it.  *We changed the statistical treatment as you advised it. We used a mixed design analysis of variance for repeated measures, with a “Group” factor in order to test if the order of the use of the prosthesis had an impact, and a “Hand” factor as within-subject factor. We have inserted the sentences below in the relevant parts and modified the figures accordingly.*  Minor revisions:  1- Abstract: *We replaced the rho symbol with r which represents the estimate of rho. We also made the changes in the manuscript.*  2- Sample size justification:  a. Lines 137-8: *“average” was replaced with the standard statistical terminology “mean”.*  b. *We specified that 25 represent the standard deviation.*  *The statistical method initially used to determine the number of participants was based on the hypothesis of a comparison of means with a student test for a power of 90% with an alpha threshold of 0.05. Since we have changed the statistical design as you advised, we have chosen to remove this part of the manuscript*  3- Line 238-9: *The “customary descriptive statistics” were mean and standard deviation.*  4- Line 239: *Indeed we made a typographical error. We rewrote: “Quantitative variable was described by numbers. Qualitative variables were described by percentages and frequencies”.*  5- Line 247: *We replaced the phrase “The alpha-risk was set at 5%” with “P-values less than 0.05 were considered statistically significant.”*  6- *Throughout the manuscript "average" was replaced by "mean".*  7- The p-value associated with a correlation is a test of the null hypothesis: correlation equal to zero; however, the absolute magnitude of the coefficient indicates the strength of the linear relationship between two variables. In general, the strength or correlation coefficient is the more important statistic to focus on.  Below is a table for interpreting correlation coefficients:  Coefficient (absolute value) Interpretation  0.90 - 1.0 Very Strong  0.70 - 0.89 Strong  0.40 - 0.69 Moderate  0.10 - 0.39 Weak  less than 0.10 Negligible correlation  *We rewrote the paragraph concerning correlation between shoulder abduction and wrist settings (p.317 à 320) by interpreting correlation coefficients:*  *“When assessing the effect of wrist settings, a significant strong negative correlation was found between shoulder abduction and flexion with the Axon-Hook (r =-0.86075; p=0.0061). The correlation between shoulder abduction and radial deviation with the Greifer was weak and not significant (r=0.38557; p=0.3455)”.*  We are returning our edited manuscript to you to conform to the plosone submission requirements stiplulated in your letter of February 19, 2021. Please accept our apologies for these omissions.  Nous vous renvoyons notre manuscrit édité pour vous conformer aux exigences de soumission de plosone stipulées dans votre lettre du 19 février 2021. Veuillez accepter nos excuses pour ces omissions.  We refer our manuscript amended to make it consistent with PlosOne stiplulées submission requirements in your letter of February 19, 2021. Please accept our apologies for these omissions.  Nous renvoyons notre manuscrit amendé pour le rendre conforme aux exigences de soumission de PlosOne stiplulées dans votre lettre du 19 février 2021. Veuillez accepter nos excuses pour ces omissions.  Impossible de charger les résultats complets  Réessayer  Nouvel essai…  Nouvel essai…  **Reviewer #2:**  1) In the paper it is not clear, why the authors have limit themselves just for 2 myoelectric prosthetic devices. Also it seems that conducting this research study limiting just for 2 devices will not provide substantial research outcome.  *We have effectively limited this study to two myoelectric hooks. Greifer and Axon Hook were the two most prescribed hooks in France. The Greifer was the most widely used hook because it was the only one covered by the French healthcare reimbursement system. We wanted to compare it with another hook that had different features, which the Axon-Hook responded to.*  2) It is acceptable for having eight participants for this study. But as mentioned in the paper, majority (or some) participants had already being used one of the prosthetic devices that have been considered in this study. So it brings biased experimental output and decrease the quality of research outcome.  *Yes indeed it would have been better than no patient has ever used Greifer but:*  *- We had to include people whose professional or life project would require the use of a myoelectric hook. As stipulated on page 8 of the paragraph [Materials and Methods/Participants] of the manuscript: “their professional activity or their life project justified the use of a myoelectric hook”. So as the Greifer is the most used myoelectric hook in France, some of the participants were already using this device. Axon-Hook was more recent and presented different features than Greifer.*  *-If it is suggested that experience has a bearing on the compensations, they should therefore have been lower among previous users of Greifer. However, they are more important with the Greifer than with the Axon Hook. This is indeed a methodological bias, but it is not the prior use of Greifer that can explain our results. On the contrary, there is a risk of underestimating the effect.*  3) In my point of view, for comparisons like theses, just one experimental test is not enough (i.e. BBT). You can obtain results from multiple test and then thoroughly analyzed. This will bring good research outcome for the audience.  *The patients only performed the BBT. Indeed, for technical reasons induced by the handover to the Laboratory (biomechanical model, placement of markers, use of data, etc.), it is difficult to adapt and analyze the results of complex functional motor tests/tasks existing ones. The BBT is the most used one in the literature regarding motion analysis (Kontson and al. 2017(a), 2017(b), 2020); its cyclical nature makes it ideal for motion capture. BBT is a validated timed measure of upper limb performance. Performed in a seated position, it lends itself very well to the evaluation of shoulder abduction.*  4) Lastly, it is good to consider several performance metrics, without being stuck into one performance metric (i.e. number of blocks moved within given time) or performance metric within the same aspect (i.e. performance at shoulder movements)  *Thank you! Precisely, the Box and Blocks test is the one that best met our objective of simultaneously evaluating these two types of performance metrics.*  Constance Billon |
| --- | --- |
|  |  |
