# Supplementary material for: Comparison of compensatory shoulder movements, functionality and satisfaction in transradial amputees fitted with two prosthetic myoelectric hooks
Source: PLoS One. 2023 Feb 2;18(2):e0272855. doi: 10.1371/journal.pone.0272855 (PMC9894487; doi:10.1371/journal.pone.0272855)
Supplement: Attachment — Submitted filename: Response to Reviewer.docx [file pone.0272855.s005.docx]

|   Centre Louis Pierquin  **75, Boulevard Lobau**  **54042 NANCY CEDEX**  Tél. : 03 83 52 67 50  Fax : 03 83 52 67 69  Direction Médicale  **Pr. J. PAYSANT, PU-PH**  Dr. M. BADIN, CCA  Dr. N. BERTONI, psychiatre  Centre de Nancy  **Dr. N. MARTINET**  Dr. B. CLAUDON  Dr. A. DATIE  Dr. A. FOISNEAU-LOTTIN  Dr. C. FRENAY  Dr. S. HENRY-CATALA  Dr. B. LASSALLE-KINIC  Dr. I. LOIRET  Dr. A. TOUILLET  Laboratoire du Mouvement  et Explorations  Pr. C. BEYAERT, PU-PH  Centre de Lay-Saint-Christophe  Dr. J-M. BEIS  Dr. M. BADIN  Dr. A. FRASER  Dr. H. GUESDON  Dr. N. KHALIL  Dr. L. LE CHAPELAIN  **Centre de Scy-Chazelles**  **Dr. I. DEDENON-MAYER**  Dr. S. DAGTEKIN  Dr. A. WALTER-KULL  Centre de Flavigny  Dr. J. BASTIEN  Dr. F. DALMONT  Dr. S. REGEF  CEM de Flavigny  E.R.E.A. de Flavigny  **D.I.M.**  Dr. M. DEGASPERI  Dr. L. LE CHAPELAIN  Pharmacie  Mme A-S. LAUSCH  Mme E. CONRARD  Mme H. OTTOMANI-MOUZE  **Cellule Recherche et Innovation**  J. PIERRET  **Réédoc**  C. GRANDIDIER | Constance Billon, PhD.  Service Explorations Fonctionnelles, Laboratoire du Mouvement  Centre Louis Pierquin, Institut Régional de Réadaptation, UGECAM  75 Boulevard Lobau, CS 34209 ; 54052 Nancy Cedex, France  Tel: +033383529811  [constance.billon@ugecam.assurance-maladie.fr](mailto:constance.billon@ugecam.assurance-maladie.fr)  Bijan Najafi  Academic Editor  PlosOne Journal  June 14, 2022,  Dear Mr. Najafi,  Thank you for taking the time to review our manuscript untitled “Comparison of compensatory shoulder movements, functionality and satisfaction in transradial amputees fitted with two prosthetic myoelectric hooks” and associated response letter.  We have submitted our revised manuscript in order to make it conform to the additional requirements stipulated in your letter of May 5, 2022.  As per your request, we submit this review by the agreed deadline, June 18, 2022.  We appreciate the time and details provided by the single reviewer and yourself and we have incorporated the suggested changes into the manuscript to the best of our ability. The manuscript certainly benefited from these insightful editing suggestions. We hope that this version of the manuscript is now close to its publication in Plos One.  All authors have agreed to add a new author to the list of authors (the signed consent of all authors is inserted at the end of this letter). Indeed, the active participation in the elaboration of our work of Jonathan Pierret was recognized by all. Thus, the initial list of authors has changed since the last submission.  We have responded specifically to each suggestion below, starting with yours.  Editor’s suggestion: Please review your reference list to ensure that it is complete and correct. If you have cited papers that have been retracted, please include the rationale for doing so in the manuscript text, or remove these references and replace them with relevant current references. Any changes to the reference list should be mentioned in the rebuttal letter that accompanies your revised manuscript. If you need to cite a retracted article, indicate the article’s retracted status in the References list and also include a citation and full reference for the retraction notice.  *We have checked our list of references and related citations, everything is correct.*  **Reviewer #1:**Minor revisions:  1- Express the correlation coefficients and large p-values to two significant digits: *In the abstract and in all the manuscript, we expressed the correlation coefficients and p-values to two significant digits.*  2- Line 250: Remove the redundant statement, “The threshold for statistical significance (error type I rate) was set to α = 0.05”.  *We removed the redundancy in brackets: (error type I rate).*  1  3- Line 244: Specify the underlying covariance structure used in the mixed models and the criteria for selecting it.  *We had freed ourselves from the "age" and "time to amputation" covariates by ensuring that the patients had no shoulder pathologies. We had already ruled out the*  *"Greifer user" covariate. We don’t see other elements that could be covariance factors.*  4- Since no statistical power calculation has been provided, indicate if the study was a pilot or exploratory trial. In brief, provide the rationale for why no formal statistical power justification is needed*.*  *We added in the paragraph 'study design': Based on observations made during recordings in routine clinical practice, the difference between the two mean shoulder abduction was estimated at 25°. To be able to detect such difference with a power of 90 using a threshold for statistical significance of 0.05, we needed to include 8 patients.*  5- If the interaction effect is significant, provide an interpretation of the results, but do not test main effects because the tests for main effects are uninteresting in light of significant interactions. If interaction effects are non-significant, drop the interaction effects from the model and test the main effects. Determining which results to present when testing interactions is often a multi-step process.  *Thank you for this comment which has helped us to improve the quality of our statistical analysis. As you requested, we excluded the interaction effect from the analysis when it was not significant. We have specified this in the text as follows: "When the interaction effect was not significant, it was dropped from the analysis and only the main effects were tested."*  *The result section has been updated to match the new analysis. The discussion has been adapted.*  6- Lines 323 and 326: Provide more precise p-values, instead of p<0.05.  *We replaced the first p<0.05 with p=0.017 and the second with p=0.014.*  7- In the figures, replace "average" with "mean."  *In Figures 5 et 7 "average" was replaced by "mean".*  Constance Billon |
| --- | --- |
|  |  |

2

**Consent to modification by all authors, done at Nancy on 14 June, 2022**

Amélie Touillet certifies here to consent to the modification of the author list:

Constance Billon-Grumillier certifies here to consent to the modification of the author list:

Jonathan Pierret certifies here to accept that his name be added to the author list:

Pierrick Herbe certifies here to consent to the modification of the author list:

Noël Martinet certifies here to consent to the modification of the author list:

3

Isabelle Loiret certifies here to consent to the modification of the author list:

Jean Paysant certifies here to consent to the modification of the author list:

4
